# Combination therapy with T cell engager and PD-L1 blockade enhances the antitumor potency of T cells as predicted by a QSP model

**DOI:** 10.1136/jitc-2020-001141

**Published:** 2020-08-27

**Authors:** Huilin Ma, Hanwen Wang, Richard J Sové, Jun Wang, Craig Giragossian, Aleksander S Popel

**Affiliations:** 1 Department of Biomedical Engineering, Johns Hopkins University School of Medicine, Baltimore, Maryland, USA; 2 Biotherapeutics Discovery Research, Boehringer Ingelheim Pharmaceuticals Inc, Ridgefield, Connecticut, USA; 3 Department of Oncology and Sidney Kimmel Comprehensive Cancer Center, Johns Hopkins Medicine, Baltimore, Maryland, USA

**Keywords:** biomarkers, tumor, B7-H1 antigen, CD8-positive T-Lymphocytes, computational biology, immunotherapy

## Abstract

**Background:**

T cells have been recognized as core effectors for cancer immunotherapy. How to restore the anti-tumor ability of suppressed T cells or improve the lethality of cytotoxic T cells has become the main focus in immunotherapy. Bispecific antibodies, especially bispecific T cell engagers (TCEs), have shown their unique ability to enhance the patient’s immune response to tumors by stimulating T cell activation and cytokine production in an MHC-independent manner. Antibodies targeting the checkpoint inhibitory molecules such as programmed cell death protein 1 (PD-1), PD-ligand 1 (PD-L1) and cytotoxic lymphocyte activated antigen 4 are able to restore the cytotoxic effect of immune suppressed T cells and have also shown durable responses in patients with malignancies. However, both types have their own limitations in treating certain cancers. Preclinical and clinical results have emphasized the potential of combining these two antibodies to improve tumor response and patients’ survival. However, the selection and evaluation of combination partners clinically is a costly endeavor. In addition, despite advances made in immunotherapy, there are subsets of patients who are non-responders, and reliable biomarkers for different immunotherapies are urgently needed to improve the ability to prospectively predict patients’ response and improve clinical study design. Therefore, mathematical and computational models are essential to optimize patient benefit, and guide combination approaches with lower cost and in a faster manner.

**Method:**

In this study, we continued to extend the quantitative systems pharmacology (QSP) model we developed for a bispecific TCE to explore efficacy of combination therapy with an anti-PD-L1 monoclonal antibody in patients with colorectal cancer.

**Results:**

Patient-specific response to TCE monotherapy, anti-PD-L1 monotherapy and the combination therapy were predicted using this model according to each patient’s individual characteristics.

**Conclusions:**

Individual biomarkers for TCE monotherapy, anti-PD-L1 monotherapy and their combination have been determined based on the QSP model. Best treatment options for specific patients could be suggested based on their own characteristics to improve clinical trial efficiency. The model can be further used to assess plausible combination strategies for different TCEs and immune checkpoint inhibitors in different types of cancer.

## Background

Colorectal cancer (CRC), especially metastatic CRC (mCRC) with proficient mismatch repair (pMMR) or microsatellite stable (MSS) tumors, is one of the leading causes of cancer-associated deaths in USA.^1^ In recent years, immune checkpoint inhibitors (ICIs) have achieved a durable clinical response in patients with melanoma, non-small-cell lung cancer (NSCLC) and other cancer types.^2^ However, the results of testing these drugs in mCRC patients with pMMR or MSS tumors were disappointing.^3^ Novel therapeutic agents or combination strategies are being tested and accurate biomarker-guided patient selection is needed to determine patients who will most likely benefit from the treatment.^4 5^ Note that all abbreviations are described in online supplementary table S1.

10.1136/jitc-2020-001141.supp1 Supplementary data

During the past few years, the US Food and Drug Administration has approved the application of anti-programmed cell death protein (PD-1) inhibitors, including nivolumab (OPDIVO, Bristol-Myers Squibb) and pembrolizumab (KEYTRUDA, Merck) and the anti-PD-ligand 1 (L1) inhibitor atezolizumab (TECENTRIQ, Genentech). They have shown great potential for targeting PD-1/PD-L1 interaction in the treatment of patients with different types of cancer including melanoma, advanced NSCLC, renal cell carcinoma (RCC), head and neck squamous cell cancer, triple-negative breast cancer and microsatellite instability-high mCRC (MSI-H mCRC).^2 6^ Typically, high PD-L1 expression in cancer cells is associated with favorable prognosis and better disease-free survival in response to PD-1/PD-L1 inhibitors.^7 8^ However, CRC cells express less PD-L1 and it has been reported by Valentini *et al* that the expression of PD-L1 in MSS CRC is mainly restricted to tumor-infiltrating immune cells.^9^ This could explain why MSS CRC patients failed to respond to anti-PD-1/PD-L1 therapy.

Despite the recent failure of anti-PD-1/PD-L1 therapy in MSS CRC patients, novel bispecific T cell engagers (TCEs) have been developed and tested in treating CRC both in vitro and in vivo. Gonzalez-Exposito *et al* developed patient derived CRC organoids to explore the mechanism of T cell bispecific antibody cibisatamab (CEA-TCB) sensitivity.^10^ Bacac *et al* reported the antitumor activity of CEA-TCB in 110 cell lines and the mode of CEA-TCB mediated CRC cell lysis in a mouse tumor model.^11^ Waaijer *et al* developed a T cell-engaging bispecific antibody (BsAb) to target cell surface A33 antigen (huA33-BsAb), which is expressed in more than 95% of human colon cancers.^12^ An ongoing phase I clinical trial of MGD007 (Clinicaltrials.gov, NCT02248805), a gpA33 x CD3-BsAb, will provide more valuable information on the clinical safety of this approach.^13^

Even though there are over 500 publications listed in PubMed reporting the preclinical and clinical investigations of BsAb,^14^ treating solid malignancies, which make up 90% of all cancers, remains extremely challenging using BsAb because of their poor permeability.^15^ Although the clinical outcome of BsAb is more satisfactory in hematologic malignancies, some ongoing clinical trials have shown promising outcomes in solid tumors.^16^ A phase I study (NCT02324257, NCT02650713) led by Hoffmann-La Roche has shown the potential of CEA-TCB (RO6958688; RG7802) monotherapy in treating patients with MSS mCRC, and 45% of the patients showed either partial response or stable disease. In a combination study of CEA-TCB with atezolizumab, an anti-PD-L1 inhibitor, 82% of the patients showed either partial response or stable disease, which was an exciting breakthrough for a BsAb in a solid tumor.^17^

Although great achievements have been made by the combination therapy of BsAb with anti-PD-L1 inhibitors in solid tumors, possible disadvantages may arise such as difficulties of determining the source of side effects, drug–drug interactions, cumulative side effects and higher cost.^18 19^ In order to avoid possible side effects and risks associated with combination therapy, it is important to prospectively determine whether individual patients will derive additional benefit from combination therapy.^20 21^ Patients need to be differentiated and given the most appropriate treatment options to improve the therapeutic outcome. The establishment of predictive biomarkers is, therefore, important to maximize therapeutic benefit and guide selection of the best therapeutic approach for oncologists.^8 22^

Previous studies have demonstrated the performance and ability of quantitative systems pharmacology (QSP) modeling in determining predictive biomarkers.^23–28^ Norton *et al* developed a multiscale agent-based model of the tumor immune microenvironment, providing information for personalized treatment for individual patients.^29^ Jafarnejad *et al* built a QSP model to represent the antitumor immune response in human NSCLC and identified biomarkers for checkpoint inhibitor-based immunotherapy.^30^ Wang *et al* proposed a QSP model to determined potential predictive biomarkers to improve the antitumor response in HER2-negative breast cancer.^31^

In this work, we have extended our QSP model to include our previously developed TCE module and newly updated anti-PD-L1 module to study the efficacy of anti-PD-L1 monotherapy and the combination with TCE therapy for MSS CRC patients. We studied individual biomarkers for the three therapeutic approaches—TCE monotherapy, anti-PD-L1 monotherapy and their combination. In silico virtual clinical trials (VCTs) have been conducted to compare the response to different treatments for the same cohort of virtual patients (VPs), and to discover predictive biomarkers. Our novel QSP model enables development of biomarker-guided patient selection to improve clinical trial efficiency by providing the distributions of different biomarkers, recommend rational therapeutic regimen and alleviate the rising demand for personalized treatment.

## Methods

### Model structure

**Figure 1:**
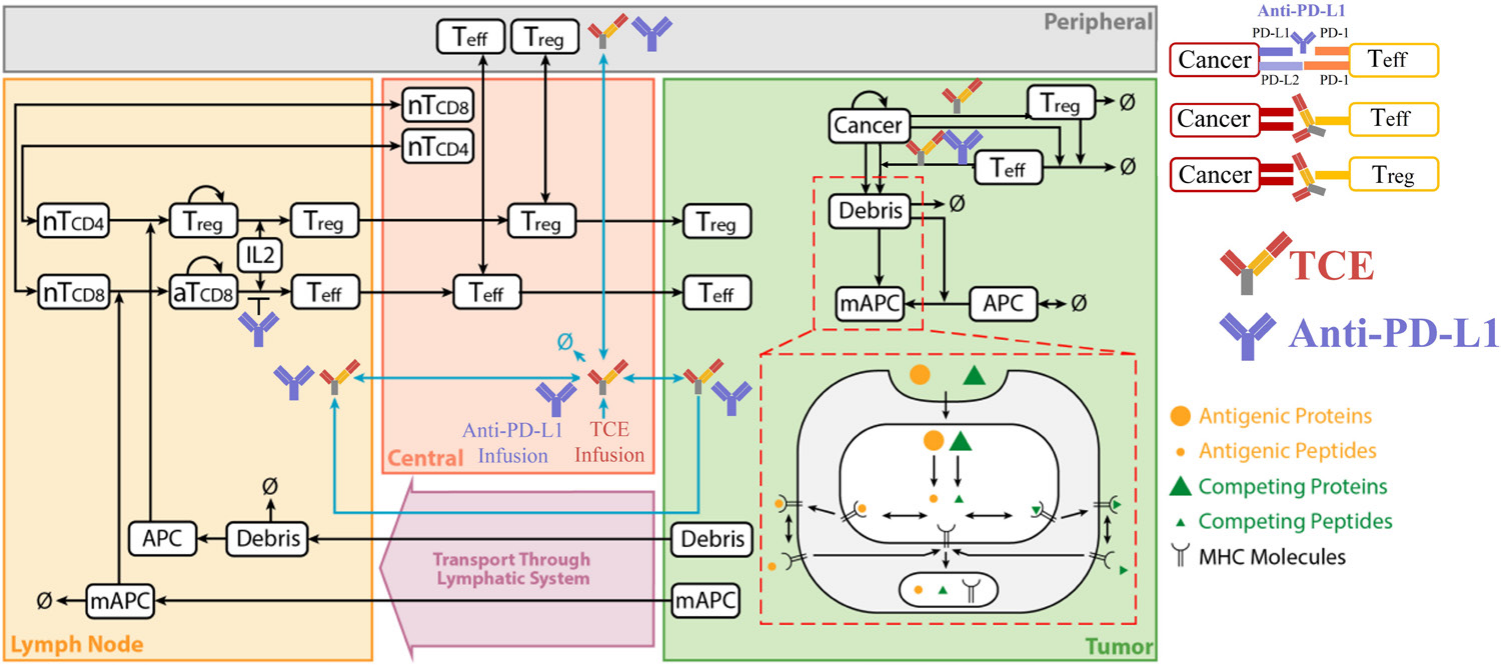
Diagram of the main cellular and molecular interactions implemented in the model (modified from^30 33^). APC, antigen-presenting cell; IL2, interleukin-2; PD-L1, programmed cell death ligand 1; TCE, T cell engager.

The QSP model used in this study was based on our previous models developed for NSCLC and TCE.^30 32 33^ The model structure includes central (blood), peripheral (other tissues and organs), tumor and tumor-draining lymph node compartments. The model is composed of several individual but interconnected modules such as cancer cell, T cell, immune checkpoint, antibody pharmacokinetics (PK), antigen presentation and TCE modules (figure 1). The dynamics of major species in each module have been described in our previous publications including tumor growth, antigen processing and presentation, T cell activation and proliferation, T cell distribution, Treg dynamics, and TCE and immune checkpoint blockade PK and pharmacodynamics (PD). All governing equations for the immune checkpoint and TCE modules have been explained in detail in the online supplementary information provided by Jafarnejad *et al*^30^ and Ma *et al*,^33^ respectively. The modular design of the model makes it readily extensible to other therapeutic agents and their corresponding PK/PD or other newly discovered physiological processes. 73 ordinary differential equations and 105 algebraic equations were used to model all biological processes involved in the model. In this work, the monotherapy of atezolizumab (MPDL3280A, RO5541267, TECENTRIQ) and the combination therapy with atezolizumab and cibisatamab (RO6958688, RG7802) were studied and compared. PK parameters of cibisatamab have been reported and PK parameters of atezolizumab were fitted to experimental data. Observed and simulated serum concentrations of atezolizumab following an intravenous dose of 1, 3, 10, 15 mg/kg and 1200 mg are provided in the supplement (online supplementary figure S1). Dynamics of cibisatamab have been calibrated and described in our previous publication.^33^ This model can be applied to most TCEs and ICIs with minor modifications. All simulations and sensitivity analyzes were performed using the SimBiology platform in MATLAB R2018b (MathWorks, Natick, Massachusetts, USA).

### Functional expression of PD-L1 on cancer and immune cells

In a previous model, Jafarnejad *et al* incorporated the dynamics of immune checkpoint blockade and demonstrated the general applicability of that module to any anti-PD-1/PD-L1 inhibitor. The model was then used to study anti-PD-1 therapy in NSCLC using nivolumab.^30^ A numberof PD-1, PD-L1, PD-L2 and other parameters involved have been carefully chosen or fitted to experimental measurements. Baseline parameters were chosen to fit a Hill function to in vitro dose-response measurements of IFNγ by Jafarnejad *et al* and thus can be applied for atezolizumab. However, our previous model only considered the expression of PD-L1 on cancer cells. The expression of PD-L1 on antigen-presenting cells (APCs) is also an important factor leading to tumor immune evasion and has been reported to have a significant effect on the outcome of immunotherapy.^34 35^

Upregulation of PD-L1 on cancer cells is believed to be the major mechanism for tumor immune evasion.^36^ However, it has been reported that dendritic cells (DCs), a major APCs, express cell-surface PD-L1 on activation by toll-like receptor ligands.^37^ DCs are responsible for initiating rapid proliferation of antitumor CD8 +T cells; however, PD-L1 signaling induced by DCs restricts the proliferative capacity of CD8 +T cells during activation, and a previous study demonstrated that DCs lacking PD-L1 expression resulted in significantly increased numbers of antigen-specific CD8+T cells.^38^ We, therefore, extended the current model with PD-L1 expression in APCs, which limits the proliferation of Teff in TdLN compartment. Blockade of PD-L1 signaling during the priming phase in the TdLN compartment will restore the normal proliferative capacity of Teff. CRC express less PD-L1 than some other types of cancer, but APCs express similar levels of PD-L1 among different cancer types. Detailed parameters used for ICIs expression level are provided in the online supplementary information as well as governing equations, species, parameters, reactions, rules, events and descriptions related to the newly added mechanism.

### Parameter sensitivity analysis

Parameter sensitivity analysis (PSA) was performed to assess the sensitivity of the QSP model to a set of parameters. Latin hypercube sampling (LHS) was used to assign the values for this set of parameters with uniform transformation such as tumor volume, density of Teff and Treg, Teff/Treg cell ratio in tumor compartment, and CD8 +T cell clonality in blood. Partial rank correlation coefficient (PRCC) analysis was performed to identify the most influential factors from the simulation results and was implemented by using the MATLAB Global Optimization Toolbox.

### Statistical analysis

Statistical analysis was performed for VPs’ subcohorts. Wilcoxon test was used to analyze the differences between responders and non-responders (NRs) under the atezolizumab/cibisatamab monotherapy and combination therapy using the ggpubr package embedded in RStudio V.1.2. The impact of sensitive parameters on the overall response rate (ORR) was also studied with 95% Agresti-Coull CI.

## Results

A virtual cohort of 2000 patients was created by LHS method. Each VP was generated with a random sample of parameter values based on the list of parameters in the PSA. The baseline number and ranges of all parameters listed in the PSA were based on clinical and experimental evidence (online supplementary tables S2 and S3), the baseline values are based on experimental measurements.^8 30 33 39 40^ Note that the ranges for parameters were chosen to be physiologically reasonable if experimental measurements are unavailable. To avoid generating implausible patients due to uncertainty in parameter ranges, several physiological parameters were used to screen VPs such as tumor diameter, T cell density in the blood, activated T cell density in the tumor and Teff to Treg ratio. A lower and upper bound of these parameters have been set based on clinical measurements.^33^ VPs who did not develop tumors or with implausible parameter values that were outside the normal physiological range were regarded as non-patients and excluded from the virtual trial. Plausible VPs were used for estimating ORR.

### In silico VCT outcomes

The ORR of atezolizumab monotherapy, cibisatamab monotherapy and combination therapy were investigated by simulating plausible VPs in each trial. In accordance with NCT02324257 and NCT02650713 trials, MSS CRC VPs in atezolizumab monotherapy were treated with atezolizumab 1200 mg Q3W. The same VPs were used in cibisatamab monotherapy and combination therapy in order to compare their responses to different therapies. They were treated with cibisatamab 60 mg QW for cibisatamab monotherapy and cibisatamab 60 mg QW with atezolizumab 1200 mg Q3W for combination therapy. Based on the screening rules, there were 1312, 1325 and 1299 VPs left in atezolizumab monotherapy, cibisatamab monotherapy and combination therapy, respectively. The simulated time-dependent percent tumor size changes are shown in online supplementary figure S2 (spider plot) following RECIST V.1.1. After 400 days, most patients who had PR/CR and SD reached convergence, where their tumor size no longer changed. Although the tumor size of some patients was still changing, the tumor size was getting smaller and did not affect the calculation of ORR. Then we calculated ORR at this time point (400 days). Among the patients in atezolizumab monotherapy, 107/1312 had PR/CR (8.2%), 91/1312 had SD (6.9%) and 1114/1750 had PD (84.9%). In cibisatamab monotherapy, 69/1325 had PR/CR (5.2%), 107/1325 had SD (8.1%) and 1149/1325 had PD (86.7%). In combination therapy, 145/1299 had PR/CR (11.2%), 114/1299 had SD (8.8%) and 1040/1299 had PD (80.0%). The ORR of cibisatamab monotherapy (5.2%) and combination therapy (11.2%) showed agreement with NCT02324257 and NCT02650713 trials (6% in cibisatamab monotherapy and 12% in combination therapy) (online supplementary table S4). To more closely mimic real patient populations, we applied these simulation results to the actual clinical trial in NCT02324257 (31 patients, cibisatamab monotherapy) and NCT02650713 (25 patients, combination therapy) by randomly sampling 31 VPs 10 000 times in cibisatamab monotherapy and 25 VPs 10 000 times in combination therapy. Although there was no atezolizumab monotherapy in these two trials, we sampled 31 VPs 10 000 times in our simulated atezolizumab monotherapy. This allowed us to obtain a 95% percentile bootstrap confidence interval (95% CI) of the ORR for the three treatments (online supplementary table S4, figure S3) and the ORR in each interval (online supplementary table S5). There has been several reports demonstrating little activity of ICIs such as atezolizumab in most MSS CRC patients,^41 42^ which was reflected in the lower limit of our estimated 95% CI for atezolizumab monotherapy.

### Statistical analysis for NRs and responders to determine potential biomarkers

**Figure 2:**
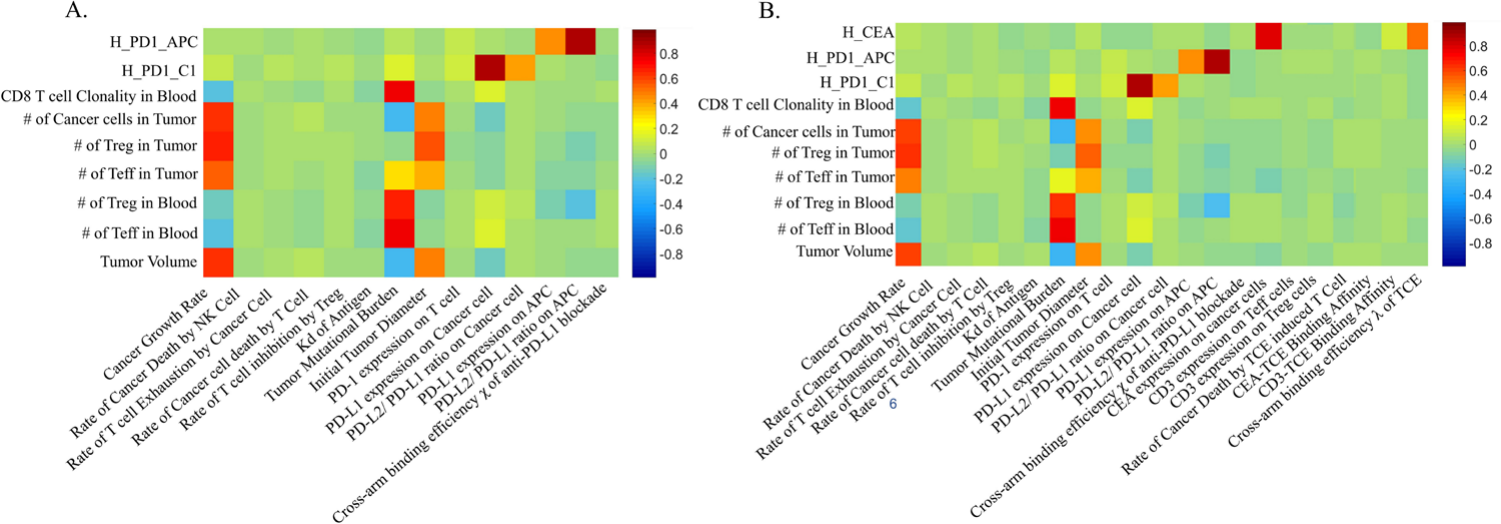
The partial rank correlation coefficient, PRCC, for individual parameters. (A) Atezolizumab monotherapy. (B) Combination therapy. APC, antigen-presenting cell; PD1, programmed cell death protein 1; PD-L1, PD-ligand 1; PRCC, Partial rank correlation coefficient; TCE, T cell engager.

**Figure 3:**
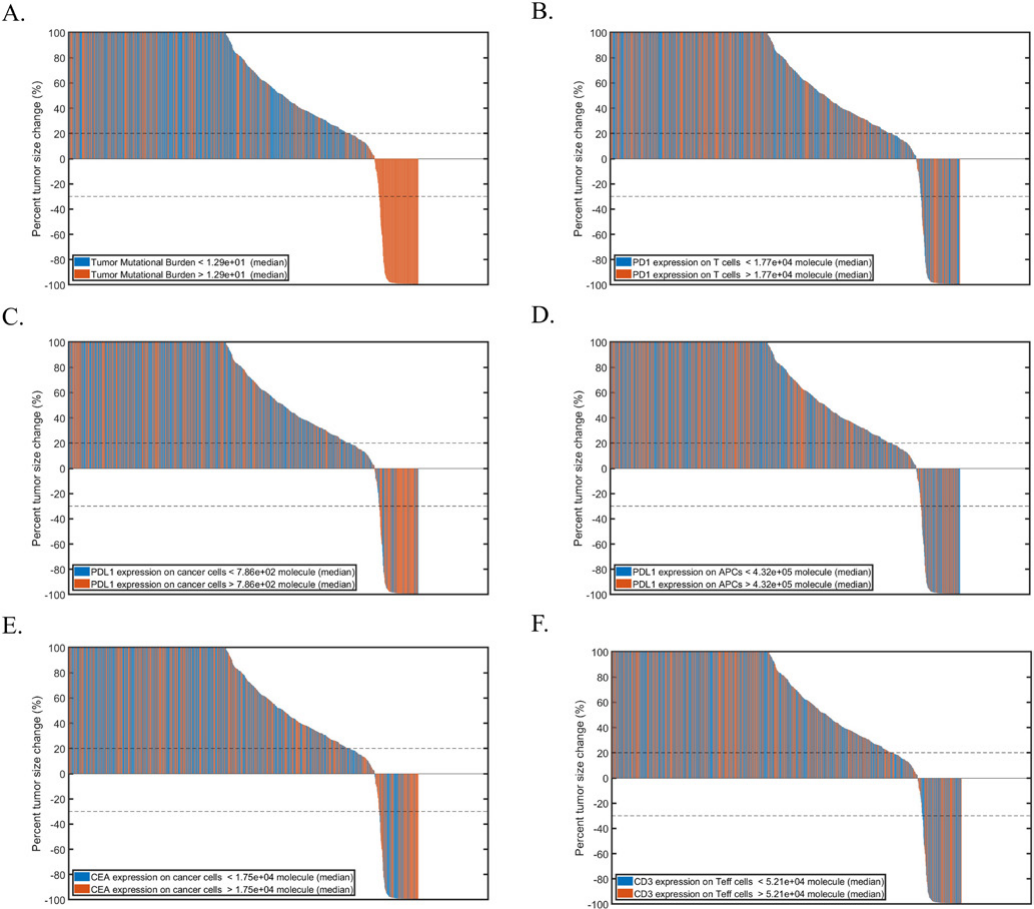
Waterfall plots of combination therapy while varying. (A) TMB; (B) PD-1 expression; (C) PD-L1 expression in cancer cells; (D) PD-L1 expression in APCs; (E) CEA expression; (F) CD3 expression in teff cells. APCs, antigen-presenting cells; CEA, carcinoembryonic antigen; PD1, programmed cell death protein 1; PD-L1, PD-ligand 1; TMB, tumor mutational burden.

PRCC was used for performing global uncertainty and sensitivity analysis to measure the degree of association between parameters and the tumor volume. In atezolizumab monotherapy and combination therapy, tumor growth rate and initial tumor diameter were significantly positively correlated to the tumor volume. Tumor mutational burden (TMB) defined as the number of clones of T cells that are activated^30 33^ and PD-L1 expression in cancer cells were negatively correlated to the tumor volume (figure 2A). In addition, CEA expression in cancer cells was also negatively correlated to the tumor volume in combination therapy (figure 2B). Waterfall plots were used to present each individual patient's response to atezolizumab (online supplementary figure S4) or combination therapy (figure 3). Obviously, higher TMB and PD-L1 expression in cancer cells corresponded to smaller tumor volume based on RECIST criteria (figure 3).

**Figure 4:**
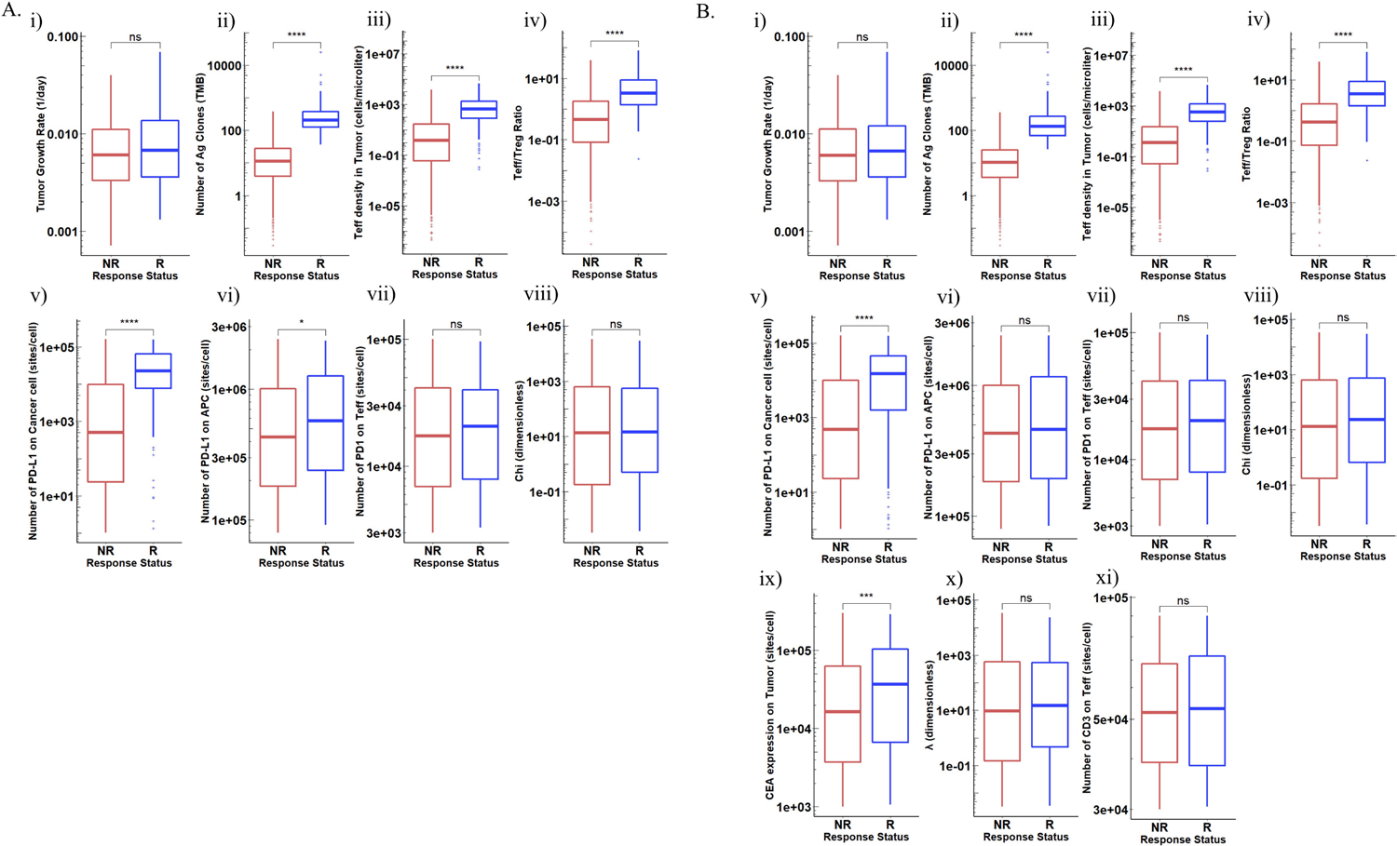
Distributions of potential biomarkers in NR and R in (A). Atezolizumab monotherapy (B). Combination therapy. (i) Tumor growth rate; (ii) TMB; (iii) ieff density in tumor; (iv) Teff/Treg ratio in tumor; (v) PD-L1 expression in cancer cells; (vi). PD-L1 expression in APCs; (vii) PD-1 expression in teff; (viii) Cross-arm binding efficiency χ of atezolizumab; (ix) CEA expression in cancer cells; (x) Cross-arm binding efficiency λ of cibisatamab; (xi) CD3 expression in teff; (xii) CD3-cibisatamab binding affinity. APCs, antigen-presenting cells; CEA, carcinoembryonic antigen; NS, not significant; PD1, programmed cell death protein 1; PD-L1, PD-ligand 1; TMB, tumor mutational burden. * P ≤ 0.05, ** P ≤ 0.01, *** P ≤ 0.001, **** P ≤ 0.0001.

Distribution of parameters of interest between R and NR are shown in figure 4. In atezolizumab monotherapy, TMB, PD-L1 on both cancer cells and APCs, Teff density as well as Teff/Treg ratio in tumor were significantly higher in responders, whereas T cell PD-1 expression and atezolizumab cross-arm binding efficiency (χ) were not significantly different between responders and NRs (figure 4A). Note that the tumor growth rate, although significantly positively correlated with tumor volume, cannot be used as an indicator to predict patients’ response. The results of cibisatamab monotherapy were completely consistent with our previous reports and will not be repeated here. Similar conclusions were observed for combination therapy. Most of the parameters that showed significant differences between R and NR in monotherapies also had significant differences between patients in combination therapy. However, in combination therapy, PD-L1 expression in APCs was not significantly different between R and NR, indicating the addition of combination therapy with cibisatamab may compensate for the loss of activated CD8 +T cells due to the PD-L1 on APCs (figure 4B).

### Comparison of Atezolizumab Monotherapy/Cibisatamab monotherapy and combination therapy

In clinical trials, it is difficult to know in advance which patients may benefit from combination therapy, particularly when only a small portion of patients are likely to derive benefits from such treatment. Prospectively being able predict patient response to combination therapies, especially for those patients who fail to respond or respond poorly to monotherapy, can lead to enhanced clinical trial design. VCTs make it easier to determine different responses to different therapies using the exact same cohort of VPs.

**Figure 5:**
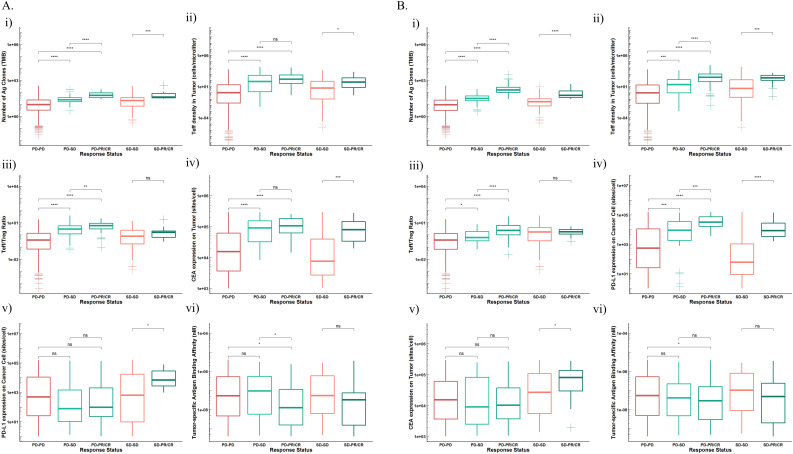
Distributions of potential biomarkers in VPS subgroups (PD-PD, PD-SD, PD-PR/CR, SD-SD, SD-PR/CR) receiving (A). Atezolizumab monotherapy versus combination therapy. (i) TMB; (ii). teff density in tumor; (iii). Teff/Treg ratio in tumor; (iv). CEA expression in cancer cells; (v). PD-L1 expression in cancer cells; (vi). tumor-specific antigen binding affinity. (B) Cibisatamab monotherapy versus combination therapy. (i). TMB; (ii) teff density in tumor; (iii) Teff/Treg ratio in tumor; (iv) PD-L1 expression in cancer cells; (v) CEA expression in cancer cells; (vi). Tumor-specific antigen binding affinity. CEA, carcinoembryonic antigen; NS, not significant; PD1, programmed cell death protein 1; PD-L1, PD-ligand 1; TMB, tumor mutational burden; VPS, virtual patients. * P ≤ 0.05, ** P ≤ 0.01, *** P ≤ 0.001, **** P ≤ 0.0001.

Here, we focused on those VPs who had PD in atezolizumab or cibisatamab monotherapy but had SD (PD-SD) or PR/CR (PD-PR/CR) in combination therapy or had SD in atezolizumab or cibisatamab monotherapy but PR/CR (SD-PR/CR) in combination therapy. Other patients who had PD or SD in both monotherapy and combination therapy without any improvement (PD-PD, SD-SD) were grouped as reference. Then, we compared the differences between patients in these groups (figure 5).

When comparing atezolizumab monotherapy and combination therapy, patients who benefited from combination therapy including PD-PR/CR, PD-SD and SD-PR/CR had significantly higher TMB, Teff density in tumor and CEA expression in cancer cells, which demonstrated their important role in predicting treatment outcomes for combination therapy. Moreover, patients in PD-PR/CR and PD-SD groups also had higher Teff/Treg ratio and cibisatamab cross-arm binding efficiency (λ) than the PD-PD group. Parameters such as TMB, Teff density and Teff/Treg ratio in tumor and CEA expression in cancer cells of patients in the PD-PR/CR group were also significantly higher than those in the PD-SD group. The distribution of these parameters can not only estimate whether patients would benefit from receiving combination therapy, but also to determine how much benefit they might receive. In addition, patients in SD-PR/CR group had higher PD-L1 expression in cancer cells than the other four groups, and these patients had stable disease after receiving atezolizumab monotherapy. With the treatment of combination therapy, they were more likely to benefit and showed better treatment outcome (PR/CR) (figure 5A).

For cibisatamab monotherapy and combination therapy, TMB and Teff density in tumor once again proved their ability to predict therapeutic effect and potential as predictive biomarkers. PD-L1 expression in cancer cells was higher in those three groups of patients with improved therapeutic effects. CEA expression was also higher in patients in group SD-PR/CR than SD-SD and their condition was improved after receiving combination therapy (figure 5B).

### Biomarker-guided patient selection

In another perspective, if we can prospectively predict which treatment is most likely to be effective for patients prior to the start of clinical trials, we would be able to select subjects for inclusion in randomized clinical trials and potentially reduce the number of subjects to recruit to improve clinical trial efficiency. Based on our virtual trials’ results, we divided all patients into five groups based on their responses to three treatments: responder to atezolizumab monotherapy only (ROA), responder to cibisatamab monotherapy only (ROC), responder to both monotherapies (ROB), responder to combination therapy only (ROCMB) and NR to any therapies.

**Figure 6:**
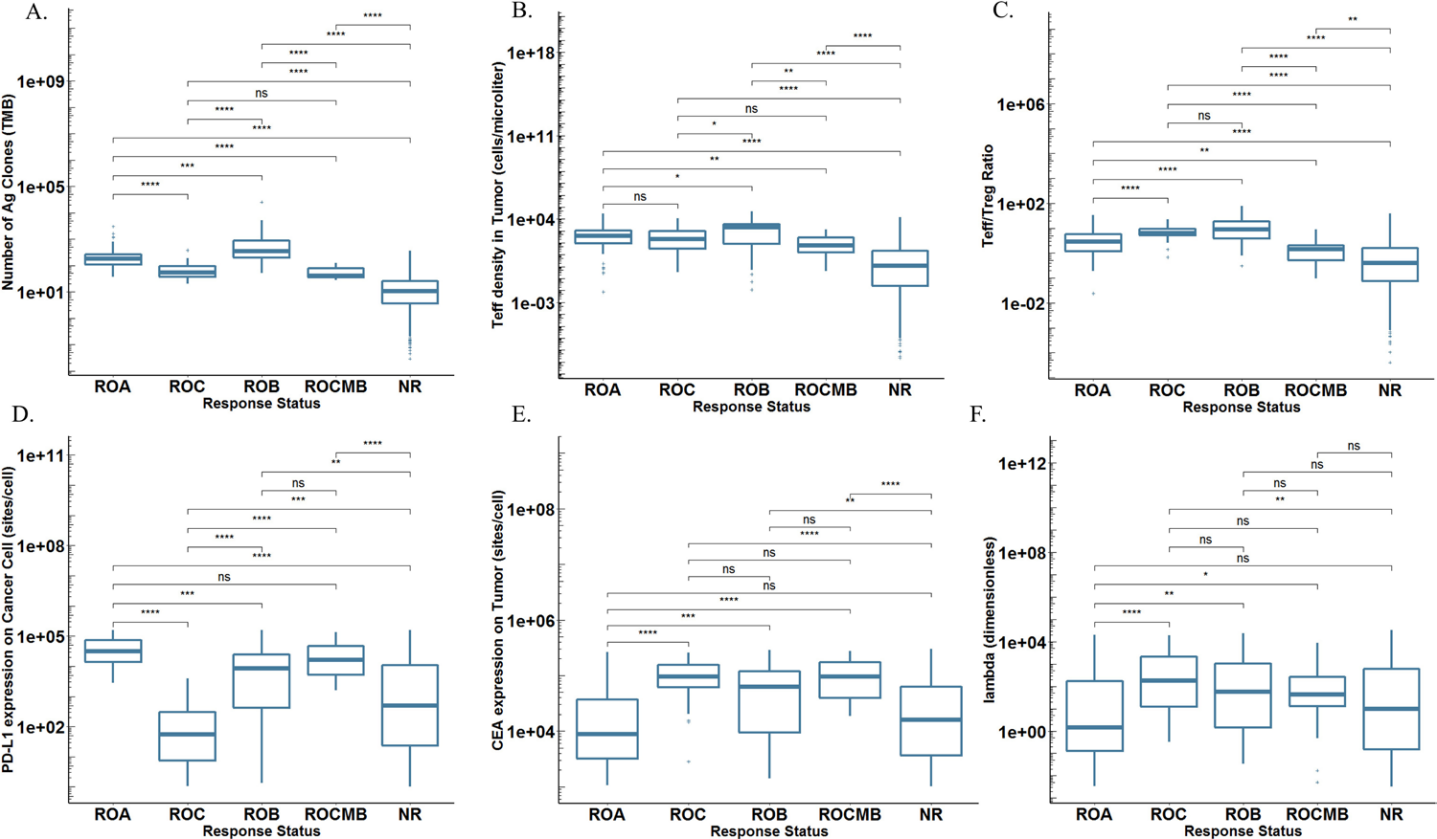
Distributions of potential biomarkers in ROA, ROB, ROC, ROCMB and NR. (A) TMB; (B) teff density in tumor; (C) Teff/Treg ratio in tumor; (D) PD-L1 expression in cancer cells; (E) CEA expression in cancer cells; (F) Cross-arm binding efficiency λ of cibisatamab. CEA, carcinoembryonic antigen; NR, non-responder; NS, not significant; PD-L1, programmed cell death ligand 1; ROA, responder to atezolizumab monotherapy; ROB, responder to both monotherapies; ROC, responder to cibisatamab monotherapy; ROCMB, responder to combination therapy; TMB, tumor mutational burden. * P ≤ 0.05, ** P ≤ 0.01, *** P ≤ 0.001, **** P ≤ 0.0001.

We computed the distribution of several potential biomarkers in these five groups, respectively (figure 6). In terms of TMB, Teff density and Teff/Treg ratio in tumor, their distributions in ROB were highest compared with any other groups. This revealed why this group of patients was able to respond to any treatments (figure 6A–C). Group ROA had highest PD-L1 expression in cancer cells around 1E4–1E5 sites/cell and the range was relatively narrower than other groups (figure 6D). Similarly, group ROC had high values of CEA expression in cancer cells and cibisatamab cross-arm binding efficiency (λ) (figure 6E, F). Group NR had the broadest distribution of all parameters, and most patients in this group had relatively low parameter values. Note that patients in group ROCMB had ideal PD-L1 and CEA expression level, however, their TMB, Teff density and Teff/Treg ratio were lower than patients in group ROA, ROC and ROB and they were the best candidates for receiving combination therapy. Ideally, if these parameters were known in advance for a real patient, we would be able to predict which group the patient might belong to, based on our predicted parameter distribution, and then determine the most suitable therapy.

### Predictive performance of different biomarkers

It is unlikely that a single biomarker will be sufficient to predict clinical outcomes in response to immune-targeted therapy. The effects of multiple factors need to be comprehensively considered to accurately predict outcomes. Nevertheless, a single biomarker can be used to predict the ORR, but the predictive performance of biomarkers is different. To determine the most predictive biomarkers, we computed and compared the predictive ability of each parameter and how its value affected the ORR of subcohorts of all VPs.

**Figure 7:**
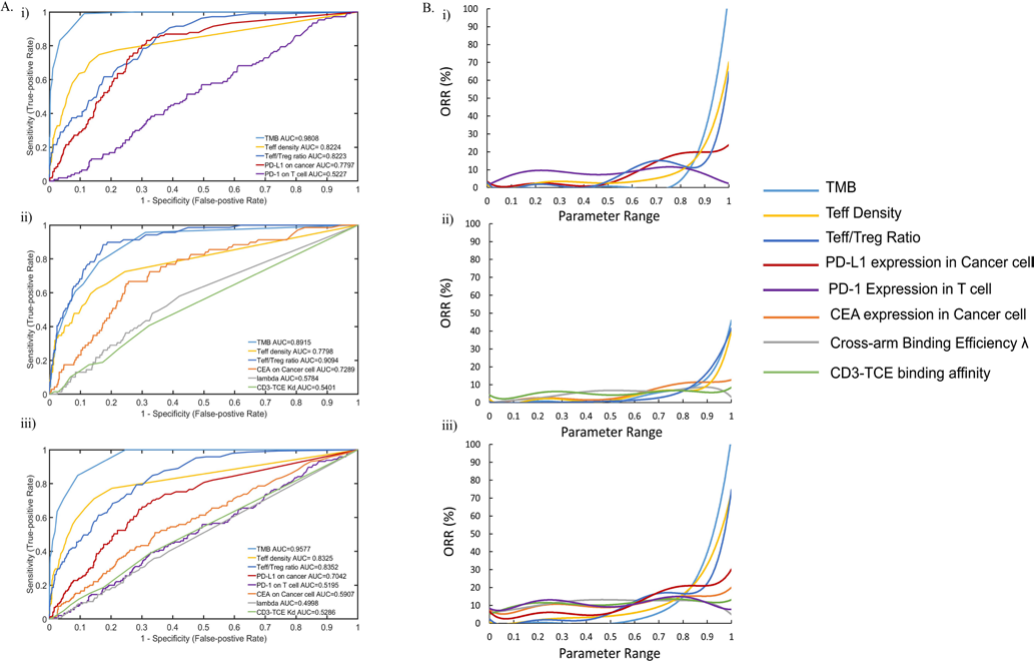
(A) ROC analysis of potential predictive biomarkers in (i) Atezolizumab monotherapy; (ii) Cibisatamab monotherapy; (iii) combination therapy. (B) Preditive ability of potential biomarkers in (i) Atezolizumab monotherapy; (ii) Cibisatamab monotherapy; (iii) combination therapy. ORR, overall response rate; PD-1, programmed cell death protein 1; PD-L1, PD-ligand 1; ROC, receiver operating characteristic; TCE, T cell engager; TMB, tumor mutational burden.

We investigated the performance of preidentified biomarkers. The results were plotted as a receiver operating characteristic (ROC) curve. TMB, Teff density and Teff/Treg ratio in tumor had high area under the curve (AUC) in all treatments, which indicated their great potential as good predictive biomarkers (figure 7). PD-L1 and CEA expression in cancer cells had high AUCs for atezolizumab monotherapy and cibisatamab monotherapy, respectively. They also had intermediate AUCs in combination therapy, however, higher AUC of PD-L1 expression demonstrated its ability to be a better biomarker than CEA expression in combination therapy (figure 7A).

Then we examined the relationship between the ORR and each parameter by calculating the ORR of patient subcohorts. First, we sorted all patients in ascending order according to a certain parameter, then every 20 patients were assigned into a group from the first patient, and the ORR of each group was calculated separately (figure 7B). All parameters were normalized to between 0 and 1 according to their range for direct comparison of the influence on the ORR. Clearly, the ORR of patients was very significantly affected by TMB. The ORR of patients with low TMB was close to 0% until the normalized parameter range was greater than 0.8 in x axis, which roughly corresponds to TMB greater than 76. When x was close to 1, the ORR of patient subcohort was about 100% and their TMB was greater than 200. Similarly, Teff density and Teff/Treg ratio were both positively correlated to patients’ ORR, and patients with ideal Teff infiltration in tumor may have higher ORR. Although the ORR increased to 20% with the increase of PD-L1 expression in patients, the effect of it on ORR was less compared with the TMB and Teff infiltration. Many patients with high PD-L1 expression were still unable to respond, most likely due to their relatively low TMB and Teff infiltration. We repeated the analysis for all three treatments; TMB showed the best potential to predict the outcomes of all treatments followed by Teff density and Teff/Treg ratio in tumor. These results are consistent with the ROC curves. According to this analysis, we were able to determine the best predictive biomarkers and therefore estimate the ORR interval that these parameter levels may correspond to.

### Function and impact of PD-L2

PD-L2 has been widely reported as a second ligand for PD-1 and inhibitor of T cell activation. Effects of PD-L2 were also included in the current QSP model. The dynamics of PD-L2 has been reported and described in the online supplementary information section 1.1.^30^ However, its role is not completely understood and expression of PD-L2 on cancer cells and APCs showed some correlation with PD-L1 expression. Taube *et al* have reported that tumor and APCs can both express PD-L2 in patients with advanced, treatment-refractory solid tumors including NSCLC, melanoma, kidney, castration-resistant prostate cancer) and CRC.^39^ They assessed PD-L2 expression in 38 tumor specimens and found that PD-L2 was less frequently expressed than PD-L1 and was almost geographically associated with PD-L1 expression. More importantly, only 1/38 tumor specimen expressed PD-L2 only without PD-L1 but 14/38 specimens expressed PD-L1 without PD-L2 and 7/38 specimens expressed both PD-L1 and PD-L2, the remaining 16/38 specimens were PD-L1 and PD-L2 negative. This indicated that PD-L2 is unlikely to be expressed alone without PD-L1 and the expression level of PD-L2 was also much lower than PD-L1 based on Cheng *et al*.^43^ Thus, in the model, we assumed that PD-L2 expression was correlated with PD-L1 and the amount of PD-L2 cannot exceed a certain threshold by defining a ratio between PD-L2 expression and PD-L1. Based on the aforementioned report, we set the lower limit of this ratio (r_PD-L2) to 0, meaning that PD-L1 can be expressed alone and set the upper limit of this ratio (r_PD-L2) to 0.07 based on the measurement of PD-L1/PD-L2 in mature DCs by Cheng *et al*.^43^ When generating VPs, each patient was assigned a certain amount of PD-L1 expression and a r_PD-L2 between 0 and 0.07. This ensured that all patients would have a realistic concurrent PD-L1 and PD-L2 expression and avoid generating a large number of implausible patient population with unrealistic PD-L1/PD-L2 expression such as PD-L2 alone or more PD-L2 than PD-L1.

However, this setting caused an issue when calculating the distribution of PD-L2 in responders and non-responders, responders showed significantly higher PD-L2 both in cancer and APCs (online supplementary figure S5), which was contrary to our expectations. Since atezolizumab did not block the interactions between PD-1 and PD-L2, less PD-L2 should be more ideal due to their inhibitory effect. However, due to the ratio we assigned for PD-L1/PD-L2 expression, patients with higher PD-L1 expression were highly likely to have higher PD-L2 than patients with lower PD-L1 expression even though the ratio was randomly assigned for each patient between 0 and 0.07, which generated misleading results. To solve this issue, we introduced an additional parameter δ into the Hill function of ICIs (equations 10 and 11 in online supplementary information). By varying the value of δ between 0 and 1, we were able to explore how PD-L2 expression affects the Hill equation and then tumor growth. We repeated the atezolizumab monotherapy again with δ and computed its distribution in responders and non-responders. The ORR was 9.3%, which was a significant improvement compared with previous one (8.2%). In addition, we computed the distribution of δ in responders and non-responders and found no significant difference between them (p>0.05) (online supplementary figure S6). To conclude, because of the low expression of PD-L2 on cancer cells and APCs, they had relatively little impact on tumor growth and patient response.

## Discussion

According to recent reports, the majority of CRC patients have MSS tumors, which accounts for 80%–85% of all CRC patients.^44^ Only CRC patients with dMMR/MSI-H CRC showed response to immunotherapy due to their highly immunogenic nature. Previous studies revealed that most MSS tumors were ‘cold’ tumors with much less PD-L1 expression in cancer cells and low TMB compared with melanoma, NSCLC and RCC.^8 39^ Thus, targeting the PD-1/PD-L1 axis was ineffective in treating MSS CRC. Nevertheless, other studies revealed a small subset of MSS CRC patients who may still benefit from anti-PD-1/PD-L1 antibodies.^45^ Therefore, the identification of predictive biomarkers for MSS CRC patients is essential to improve patient outcome. Recent clinical trials are looking at novel ICIs, combination of immunotherapeutic agents and better patient selection for immunotherapy treatment to increase response of MSS CRC patients. However, there is a combinatorial explosion of drug candidates and therapies that make clinical assessment of plausible options highly expensive and less feasible. Implementations of QSP models have become an alternative method to study different drug combinations and their efficacy. Our QSP model incorporates dynamics of ICIs and TCEs that can be applied to any anti-PD-1/PD-L1 blockades and TCEs, which can be used as a tool to study drug candidates and combination strategies in silico.

Our model has successfully conducted in silico VCTs for atezolizumab monotherapy and combination therapy. The predicted ORR showed consistency with clinical trial results. Patients receiving monotherapy with PD-1 or PD-L1 agents typically well tolerate them based on previous studies, but combination therapies are always associated with elevated risk of immune-related adverse events. The identification of predictive biomarkers is critical to optimize patient benefit and reduce risk of toxicities. Important parameters such as TMB, amount of Teff in tumor microenvironment, and PD-L1/CEA expression in cancer cells have shown potential to be predictive biomarkers. PD-L1 expression in APCs could potentially be a biomarker for atezolizumab monotherapy, but it showed no correlation with patients’ response in combination therapy. Other parameters such as cross-arm binding efficiency λ and CD3-cibisatamab binding affinity could affect the efficacy of cibisatamab monotherapy, which should be noted and carefully selected in the design of TCE.

We then explored if patients who failed to respond to monotherapies can benefit from combination therapy by studying patients in PD-PD, PD-SD, PD-PR/CR, SD-SD and SD-PR/CR groups. This analysis would help guide treatment recommendations by assessing if combination therapy may work for specific patients. We have shown how patients with different parameters responded differently. Although patients in PD-PR/CR and PD-SD groups can both benefit from combination therapy, patients in PD-PR/CR group had higher TMB, Teff density and Teff/Treg ratio in tumor, and PD-L1 or CEA expression in cancer cells than patients in PD-SD groups. However, if we only compare one parameter, the distribution of this parameter in patients of PD-PR/CR, PD-SD and PD-PD groups may overlap with other groups, resulting in false positive results, that is, high TMB patients in PD-PD group were predicted in PD-PR/CR or PD-SD groups. However, this can be avoided by comparing more parameters.

Moreover, we computed the distribution of potential biomarkers in our VPs and studied how their distribution affected patients’ response, by grouping all patients into ROA, ROC, ROB, ROCMB and NR groups. This might solve the problem of unnecessary trials and help determine the best treatment option for patients and prospectively assign them to the right group. As we mentioned before, a small subset of MSS CRC patients can still respond to anti-PD-1/PD-L1 antibodies or TCE monotherapy. These patients need to be identified instead of being potentially excluded from a therapy that could be suitable for them. In addition, some patients are unlikely to respond to monotherapies, and should receive combination therapy. There is also a subset of patients who may respond to any treatment, for them the best treatment can be chosen based on the clinicians’ experience and other factors such as potential toxicity and patients’ preferred dosing regimen. Finally, patients who may not respond to any treatment can be identified and considered for other treatment options in a timely manner. Therefore, it is essential to implement novel biomarker-guided patient selection to improve the overall efficiency of clinical trials design. Ideally, if all these potential biomarkers can be measured, the best treatment can be identified for a specific patient based on the reference ranges obtained from a virtual population. However, realistically, in the most cases, not all parameters can be measured, therefore, the performance of these biomarkers can be compared with predict efficacy and a rough prediction of ORR can be made even when there are only a few biomarkers available.

PD-L2 is commonly considered as an inhibitor of T cell activation, but its actual role in the tumor microenvironment are still being elucidated. An in vivo study of PD-L2 KO mice has shown a potential function of PD-L2 for augmenting T helper 1 and CTL responses.^46^ Another report revealed that the aggregated form of PD-L2 on DCs may suppress the interaction between PD-1 and PD-L1.^47^ Despite these reports, therapy targeting both PD-1 ligands such as PD-1 blockade still provided clinical benefit.^48^ Since the functions of PD-L2 are still not completely understood and the primary ligand of PD-1 has been proven to be PD-L1 with presence of PD-L2, we, therefore, decided not to study the impact of PD-L2 on patients’ response in our model in depth. Fortunately, the expression of PD-L2 in CRC is very low and its effect on CRC is much lower than in other types of cancer. Although there is more PD-L2 on APCs, sensitivity analysis has shown limited impact on patients’ response. As more evidence emerges to clarify the role of PD-L2, we will add these mechanisms to the model in future work.

We have extended the ICIs module with PD-L1 expression in APCs. However, a higher level of PD-L1 expression in tumor-infiltrating immune cells especially tumor-associated macrophages (TAMs) and myeloid-derived suppressor cells (MDSCs) has been detected and proved to be associated with patients’ survival.^7 49^ Since these cells are important for tumor progression, efficacy of targeting PD-L1 on tumor-infiltrating immune cells should be further studied. In terms of TAMs, their polarization toward M1 or M2 subsets in the tumor microenvironment has attracted a lot of attention and ample evidence exists that TAMs appear and behave as M2 phenotype, which is an important factor in protumorigenesis.^50^ It is, therefore, necessary to include TAMs and MDSCs to have a better understanding of the roles of PD-1/PD-L1 in different cells and of the function of TAMs polarization in the tumor microenvironment. This addition will then make our QSP model more complete in determining biomarkers and providing guidance for future clinical trials.

## Conclusion

In summary, we performed three in silico VCTs using our QSP model with an expanded ICIs module. The model reproduced clinical trial outcomes and showed good consistency with previous publications. For each therapy, we were able to identify potential patient selection biomarkers. By comparing the predicted outcomes of monotherapy and combination therapy in the same set of VPs, the model was able to identify the best treatment options for patients based on their individual characteristics. In addition, the current model can be applied to other TCEs and ICIs in different types of cancer to help assess plausible combination strategies and reduce the effort of clinical assessment. Although the knowledge gap between clinical trials and QSP modeling hinders application of these models in many respects, including the optimal selection of combination therapies, predicting toxicity, duration of response (DOR) and progression free survival, this gap will be filled as greater emphasis is placed on the collection of patient-centric biomarkers in current and future clinical trials.

10.1136/jitc-2020-001141.supp2 Supplementary data
