# Supplementary material for: Combination therapy with T cell engager and PD-L1 blockade enhances the antitumor potency of T cells as predicted by a QSP model
Source: J Immunother Cancer. 2020 Aug 27;8(2):e001141. doi: 10.1136/jitc-2020-001141 (PMC7454244; doi:10.1136/jitc-2020-001141)
Supplement: Supplementary data [file jitc-2020-001141supp001.pdf]

Combination Therapy with T cell engager and PD-L1 Blockade Enhances the Antitumor Potency of T Cells as Predicted by a QSP Model

Huilin Ma[1*], Hanwen Wang[1], Richard J. Sove[1], Jun Wang[3], Craig Giragossian[3], Aleksander S. Popel[1,2]

[1]*Department of Biomedical Engineering, Johns Hopkins University School of Medicine, Baltimore, MD, USA*

[2]*Department of Oncology and Sidney Kimmel Comprehensive Cancer Center, Johns Hopkins University, Baltimore, MD, USA*

[3]*Biotherapeutics Discovery Research, Boehringer Ingelheim Pharmaceuticals, Inc, Ridgefield, CT, USA*

[*]Corresponding author email: hma24@jhmi.edu

Supplementary Information

1.1 Updated Immune checkpoint blockade dynamics for atezolizumab

Immune checkpoint blockade dynamics has been described by Jafarnejad et al. (1) and was expanded here for anti-PD-L1 blockades. PD-1 expressed in Teff interacts with PD-L1 and PD-L2 on cancer cell in the immunological synapse (Eqs. 1 and 2). The formation of PD1_PDLX (PD1_PDL1 + PD1_PDL2) (Eq. 6) will cause reduced cancer killing by Teff. The expression of PD-1 on T cells and PD-L1/PD-L2 on cancer cells or APCs were estimated based on measurements using quantitative flow cytometry using calibrated fluorescent beads (2, 3). Atezolizumab binding to the PD-L1 was modeled using a bivalent model of antibody receptor interaction on cell surface by introducing a cross-arm binding efficiency X (Eqs. 3,4 and 5). Relevant governing equations for the dynamics of the checkpoint molecules in the immune synapse are summarized below based on previous publications (1, 4, 5):

$$\frac{dPD1\_PDL1}{dt} = k_{on,PD1\_PDL1} \cdot PD1 \cdot PDL1 - k_{off,PD1\_PDL1} \cdot PD1\_PDL1 \quad (1)$$

$$\frac{dPD1\_PDL2}{dt} = k_{on,PD1\_PDL2} \cdot PD1 \cdot PDL2 - k_{off,PD1\_PDL2} \cdot PD1\_PDL2 \quad (2)$$

$$\frac{dPDL1\_Atezo}{dt} = 2k_{on,PDL1\_Atezo} \cdot PDL1 \cdot Atezo/f_{tum} - k_{off,PDL1\_Atezo} \cdot PDL1\_Atezo \quad (3)$$

$$\frac{dPDL1\_Atezo\_PDL1}{dt} = X\left(\frac{k_{on,PDL1\_Atezo}}{A_{syn}d_{syn}N_A}\right) \cdot PDL1_{Atezo} \cdot PDL1 - 2 * k_{off,PDL1\_Atezo} \cdot PDL1\_Atezo\_PDL1 \quad (4)$$

$$\frac{dPDL1}{dt} = -k_{on,PD1\_PDL1} \cdot PD1 \cdot PDL1 + k_{off,PD1\_PDL1} \cdot PD1_{PDL1} - 2k_{on,PD1\_Nivo} \cdot PD1 \cdot \frac{Nivo}{f_{tum}} + k_{off,PDL1\_Atezo} \cdot PDL1\_Atezo - X\left(\frac{k_{on,PDL1\_Atezo}}{A_{syn}d_{syn}N_A}\right) \cdot PDL1\_Atezo \cdot PDL1 + 2 * k_{off,PDL1\_Atezo} \cdot PDL1\_Atezo\_PDL1 \quad (5)$$

where $k_{on,PD1\_X}$ and $k_{off,PD1\_X}$ are the on and off rates for interactions between PD-1 and X (PD-L1, PD-L2, and atezolizumab), f_{tum} is the porosity in the tumor, X is the intrinsic antibody cross-arm binding efficiency, A_{syn} is surface area of the synapse, d_{syn} is the thickness of the confinement space between the two cells, and N_A is Avogadro's number. $k_{on,PDL1\_Atezo}$ was converted to units of $1/(molecule.s)$ using the synapse sizes ($A_{syn} * d_{syn}$) and Avogadro's number N_A. The number of bound PD-L1 molecules on cancer cells or APCs was translated to Teff exhaustion using a Hill equation.

$$PD1\_PDLX\_CC = PD1\_PDL1\_CC + PD1\_PDL2\_\mathrm{CC} \quad (6)$$

$$PD1\_PDLX\_APC = PD1\_PDL1\_APC + PD1\_PDL2\_\mathrm{APC} \quad (7)$$

Here PD-L1/PD-L2 can be either expressed on cancer cells or APCs. PD-1/PD-L1/PD-L2 dynamics is assumed to be similar in T cell - cancer cell and T cell - APC, thus only a single checkpoint module was used in this study to reduce the size of the model. However, to differentiate the differences of PD-L1/PD-L2 expression in cancer cell and APC, Eqs. 1-5 are used simultaneously for cancer cell and APC when the simulations are running, resulting in two species $PD1\_PDLX\_CC$ and $PD1\_PDLX\_APC$, which represent the number of bound PD-1 – PD-L1/PDL2 in T cell - cancer

cell and T cell – APC synapse. Then, the number was translated to Teff exhaustion using a Hill equation.

$$H\_PD1\_PDLX\_CC = \left(1 - \frac{PD1\_PDLX\_CC^2}{PD1\_PDLX\_CC^2 + K^2_{PD1\_PDLX\_CC}}\right) \quad (8)$$

$$H\_PD1\_PDLX\_APC = \left(1 - \frac{PD1\_PDLX\_APC^2}{PD1\_PDLX\_APC^2 + K^2_{PD1\_PDLX\_APC}}\right) \quad (9)$$

As we mentioned in the main paper in section 3.6, PD-L2 expression showed ambiguous results. To study the impact of PD-L2, we introduced another parameter δ in the Hill functions (6) and (7) and added it into the parameter sensitivity analysis and assigned its range between 0 and 1.

$$PD1\_PDLX\_CC = PD1\_PDL1\_CC \ + \delta \ * PD1\_PDL2\_\mathrm{CC} \quad (10)$$

$$PD1\_PDLX\_APC = PD1\_PDL1\_APC \ + \delta \ * PD1\_PDL2\_\mathrm{APC} \quad (11)$$

1.2 Updated T cell activation and proliferation

T cells activation in TdLN is based on a two-step priming model described by Jafarnejad et al [REF]. In the main paper, the function of PD-L1/PD-L2 expression in APCs has been discussed. After naïve T cells being activated by mAPCs in the first step, the PD-L1/PD-L2 expression in APCs is assumed to limit the proliferation of activated T cells into functional effector T cell by introducing an inverse Hill function (Eq. 9).

$$TCPR = \frac{k_{\mathrm{aTCD8,prolif}}}{n_{\mathrm{prolif}}} \cdot 2^{n_{\mathrm{prolif}}} \cdot T_{\mathrm{activated,CD8}} * H\_PD1\_PDLX\_APC \quad (12)$$

where $TCPR$ is T cell proliferation rate, $k_{\mathrm{aTCD8,prolif}}$ is doubling rate of activated T cells, n_{prolif} is the number of generations T cells proliferate, and $T_{\mathrm{activated,CD8}}$ is the number of activated T cells in the TdLN. The number of generations that activated T cells proliferate (division destiny) before leaving

the TdLN depends on TCR engagement, co-stimulation signal through CD28, and IL-2 receptor stimulation.

1.3 Updated Tumor growth

Teff killing rate (TKR) is expressed as an inverse Hill equation of immune checkpoint inhibitors and T cell engagers as follows, which remains the same as reported by Ma et al. (6).

$$H\_TCE = \frac{CEACEA\_TCE\_TeffCD3^3}{CEACEA\_TCE\_TeffCD3^3 + K^3_{CEACEA\_TCE\_TeffCD3}} \quad (13)$$

$$TKR = k_{C,death,TCET_{eff}} \frac{C \cdot T_{eff}}{C + T_{tot}} * H\_TCE + k_{C,death,T_{eff}} \frac{C \cdot T_{eff}}{C + T_{tot}} * H\_PD1\_PDLX\_CC \quad (14)$$

The number of bound CEACEA_TCE_TeffCD3 was translated to cancer cell killing rate by Teff cells using a Hill equation. The immune checkpoint blockade dynamics elaborated by Jafarnejad et al. (1). was combined with T cell engager dynamics in the Teff cell killing rate (TKR).

Here $k_{C,death,T_{eff}}$ is basal cancer killing rate by Teff and $k_{C,death,TCET_{eff}}$ is additional cancer killing rate by Teff activated by TCE, C is the total number of cancer cells in the tumor compartment, T_{eff} is total number of Teff in the tumor and T_{tot} is total number of T cells in the tumor, $CEACEA\_TCE\_TeffCD3$ is the total number of engaged CEA CD3 molecules bridged by TCE in the synapse, and $K_{CEACEA\_TCE\_TeffCD3}$ is sensitivity of TKR to $CEACEA\_TCE\_TeffCD3$. Formation of $CEACEA\_TCE\_TeffCD3$ will increase TKR according to the Hill equation (H_TCE), and formation of PD1_PDLX_CC will slow down TKR according to the inverse Hill equation. Details of TCE dynamics were provided by Ma et al. (6).

Supplementary Figures

Pharmacokinetics

Pharmacokinetic of atezolizumab was modelled following the same physiologically-based pharmacokinetic model as described by Jafarnejad et al. (1). The plasma concentration of atezolizumab in our model was fitted to standard pharmacokinetic two-compartment model (Fig. S1). PK parameters were fitted to the data reported and the simulated plasma concentration of atezolizumab together with the clinical measurements at dose levels of 1, 3, 10, 15 mg/kg and 1200 mg in the central compartment (7).

$Atezo_P$, $Atezo_C$, $Atezo_{LN}$, $Atezo_T$ indicate atezolizumab concentration in peripheral, central, TdLN and tumor compartment, respectively.

$$V_C \frac{dAtezo_C}{dt} = q_P(Atezo_P - Atezo_C) + q_{LN}(Atezo_{LN} - Atezo_C) + q_T(Atezo_T - Atezo_C) + q_{LD}Atezo_{LN} - CL * Atezo_C \quad (13)$$

$$V_P \frac{dAtezo_P}{dt} = q_P(Atezo_C - Atezo_P) \quad (14)$$

$$V_T \frac{dAtezo_T}{dt} = q_T(Atezo_C - Atezo_T) - q_{LD}Atezo_T \quad (15)$$

$$V_{LN} \frac{dAtezo_{LN}}{dt} = q_{LN}(Atezo_C - Atezo_{LN}) + q_{LD}Atezo_T - q_{LD}Atezo_{LN} \quad (16)$$

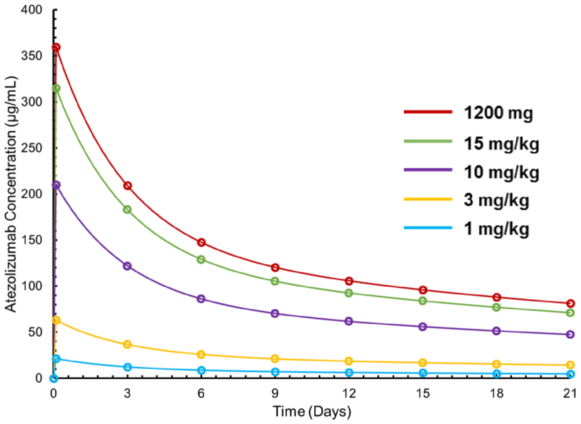

Figure S1. Simulated (solid lines) and measured (dots) atezolizumab plasma concentration.

A.

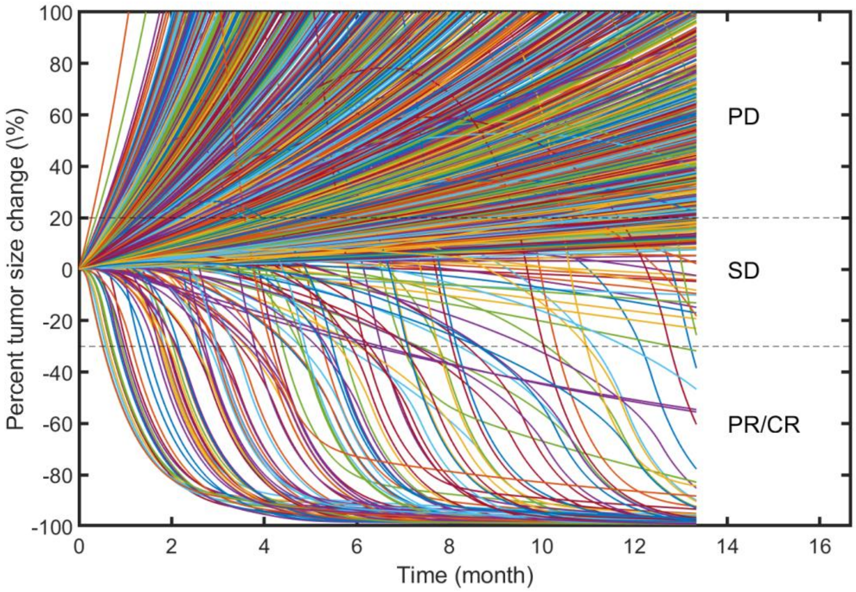

B.

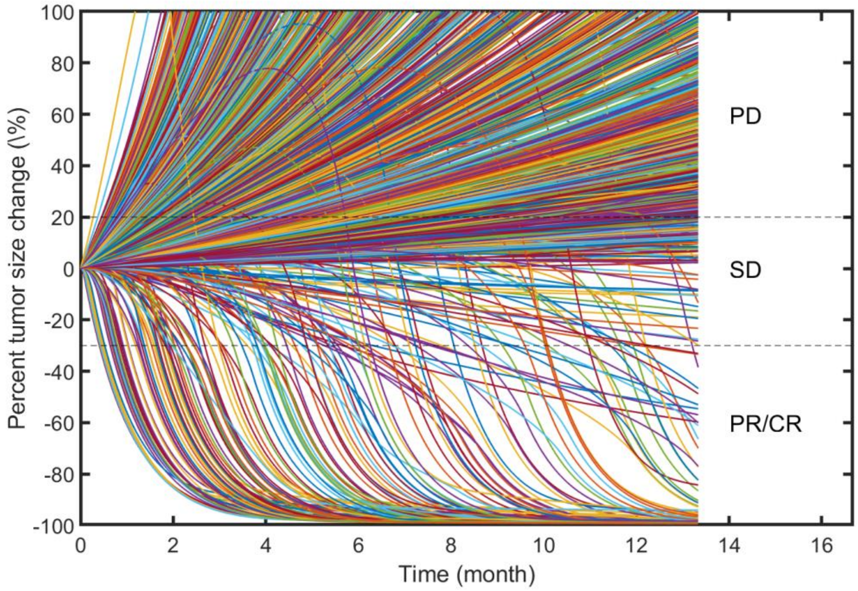

Figure S2. Percent change in tumor size represented using RECIST criteria (a "spider" plot). A. Atezolizumab monotherapy (1312 virtual patients). B. Combination therapy (1299 virtual patients).

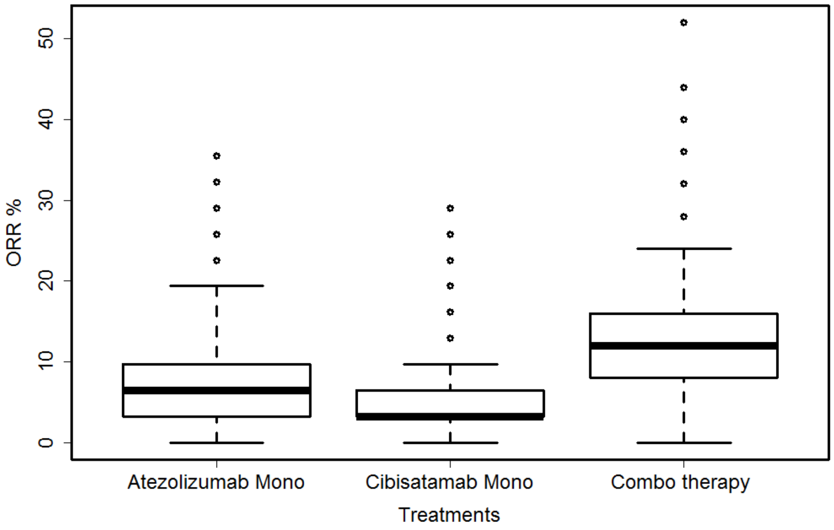

Figure S3. Bootstrapping results for atezolizumab monotherapy, cibisatamab monotherapy and combination therapy (10,000 bootstrap samples).

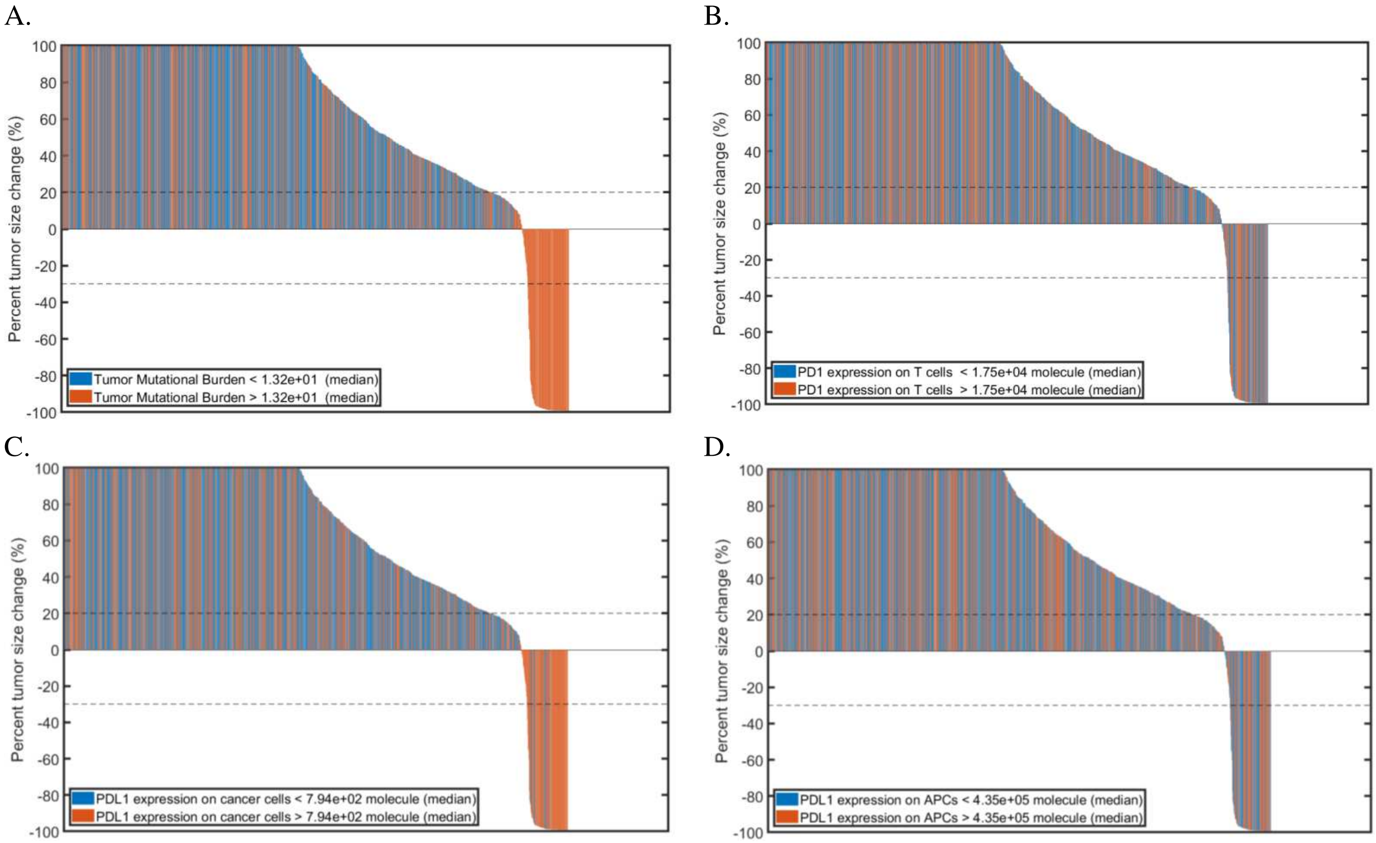

Figure S4. Waterfall plots for atezolizumab monotherapy while varying A. TMB; B. PD-1 expression; C. PD-L1 expression in cancer cell; D. PD-L1 expression in APCs.

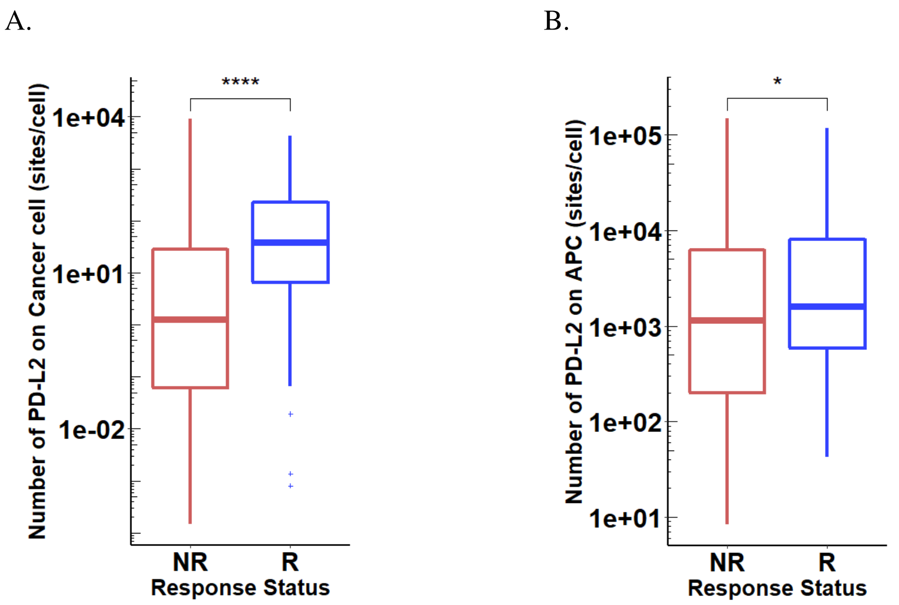

Figure S5. Distributions of potential biomarker in NR and R in atezolizumab monotherapy. A. PD-L2 expression in cancer cells; B. PD-L2 expression in APCs.

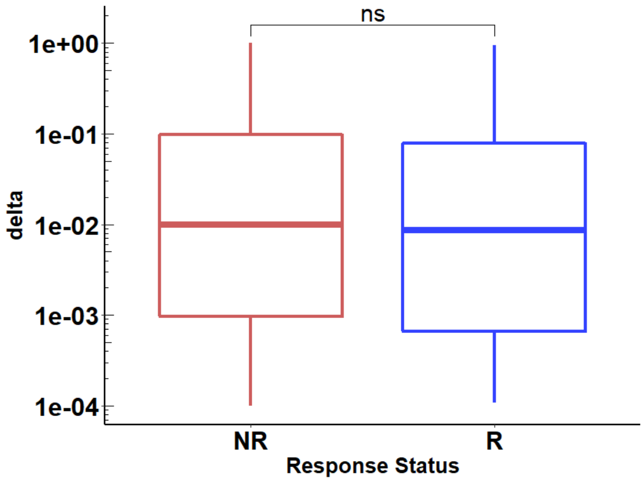

Figure S6. Distribution of δ in NR and R of atezolizumab monotherapy.

Supplementary Tables

Table. S1 Abbreviations

Abbreviation	Definition
AUC	Area under the curve
BsAb	Bispecific antibodies
CEA	Carcinoembryonic Antigen
CEA-TCB	Carcinoembryonic Antigen T-Cell Bispecific Antibody
CR	Complete Response
CTLA-4	Cytotoxic T-lymphocyte-associated protein 4
EpCAM	Epithelial cell adhesion molecule
HNSCC	Head and neck squamous cell cancer
LHS	Latin hypercube sampling
mAb	Monoclonal antibody
mCRC	Metastatic Colorectal Cancer
MDSCs	Myeloid-derived suppressor cells
MHC	Major Histocompatibility Complex
moTTC	Monovalent TCEs Ternary Complex
MSS	Microsatellite Stable
MSI-H	Microsatellite instability High
NSCLC	Non-small-cell Lung Carcinoma
ORR	Overall response rate
PD	Progressive Disease
PD-1	Programmed cell death protein 1
PD-L1	Programmed death-ligand 1
pMMR	Mismatch Repair proficient
PR	Partial Response
PRCC	Partial Rank Correlation Coefficient
PSA	Parameter Sensitivity Analysis
QSP	Quantitative Systems Pharmacology
RECIST	Response evaluation criteria in solid tumors
ROA	Responder of atezolizumab monotherapy only
ROB	Responder of both monotherapies
ROC	Responder of cibisatamab monotherapy only
ROCMB	Responder of combination therapy only
SBML	Systems Biology Markup Language
SD	Stable Disease
TAMs	Tumor-associated macrophages

Abbreviation	Definition
TCB	T Cell Bispecific
TCE	T cell engager
TCR	T-cell receptor
TdLN	Tumor-draining Lymph Nodes
Teff	Effector T cells
TILs	Tumor infiltrating T cells
TMB	Tumor Mutational Burden
TME	Tumor microenvironment
TNBC	Triple-negative breast cancer
Treg	Regulatory T cells
VCTs	Virtual clinical trials
VPs	Virtual patients

Table. S2 Atezolizumab-related Variables and Terms Used in Equations

Parameters	Definition	Unit	Value (ref)
C_PDL1_total	Total PD-L1 on tumor cell	sites/cell	160000 (3, 8)
APC_PDL1_total	Total PD-L1 on APC	sites/cell	1600000 (2, 3)
C_PDL2_total	Total PD-L2 on tumor cell	sites/cell	10400 (2, 9)
APC_PDL2_total	Total PD-L2 on APC	sites/cell	104000 (2, 9)
Teff_PD1_total	Total PD-1 on Teff cell	sites/cell	60000 (1)
Kd_PD1PDL1	Binding affinity of PD-1/PD-L1	μM	8.2 (1)
Kd_PD1PDL2	Binding affinity of PD-1/PD-L2	μM	2.3 (1)
Kd_PDL1_Atezo	Binding affinity of PD-L1/Atezolizumab	nM	0.4 (10)
χ	Intrinsic antibody cross-arm binding efficiency	dimensionless	100 (4)
n_PD1_PDLX	Hill coefficient for PD1_PDLX	Dimensionless	2 (1)
$K_{PD1\_PDLX\_CC}$	Number of PD-1/PD-L1 for half maximal inhibition of T cell killing	Molecule	250 (1)
$K_{PD1\_PDLX\_APC}$	Number of PD-1/PD-L1 for half maximal inhibition of T cell killing	Molecule	250 (1)

Species	Definition	Unit	
PD1_PDL1_CC	PD1_PDL1 complex in tumor comp	Molecule	-
PD1_PDL2_CC	PD1_PDL2 complex in tumor comp	Molecule	-
PDL1_Atezo_CC	PDL1_Atezo complex in tumor comp	Molecule	-
PDL1_Atezo_PDL1_CC	PDL1_Atezo_PDL1 complex in tumor comp	Molecule	-
PD1_PDL1_APC	PD1_PDL1 complex in LN comp	Molecule	-
PD1_PDL2_APC	PD1_PDL2 complex in LN comp	Molecule	-
PDL1_Atezo_APC	PDL1_Atezo complex in LN comp	Molecule	-
PDL1_Atezo_PDL1_APC	PDL1_Atezo_PDL1 complex in LN comp	Molecule	-
V_LN.T1	Total T cells in the LN comp	Cell	-
V_T.T1	Total T cells in the Tumor comp	Cell	-
V_C.T1	Total T cells in the central comp	Cell	-
V_P.T1	Total T cells in the Peripheral comp	Cell	-
V_LN.Atezo	Concentration of Atezolizumab in LN comp	M	-
V_T.Atezo	Concentration of Atezolizumab in tumor comp	M	-
V_C.Atezo	Concentration of Atezolizumab in central comp	M	-
V_P.Atezo	Concentration of Atezolizumab in Peripheral comp	M	-

Table. S3 Parameter Values and Ranges Used in the Sensitivity Analysis

Parameter	Baseline Value	Sensitivity test range	Unit
Tumor Growth Rate	0.005	0-0.05	1/day
Rate of cancer death by NK cell	0.00001	0.00001-0.001	1/day
Rate of T cell exhaustion by cancer cell	0.1	0.05-0.5	1/day
Rate of cancer death by T cell	3	1-8	1/day
Rate of Teff inhibition by Treg	1	0.1-1	1/day
K_D of Ag-MHC	4.0E-08	4E-10 - 4E-6	M
Number of Ag Clones (TMB)	10	0 – 2.5E4	dimensionless
Initial Tumor Diameter	3	0.5-5	cm
CEA expression in cancer cell	20000	1000-300000	molecule
CD3 expression in cancer cell	61000	30000-90000	molecule
CD3 expression in cancer cell	61000	30000-90000	molecule
Rate of Tumor Death by TCE activated Teff	1	1-10	molecule
Rate of TCE activated Treg Inhibition of Teff	2	0.1-10	1/day
koff of CEA TCE	0.00013	0.000001-0.001	1/s
koff of CD3 TCE	0.00075	0.00001-0.01	1/s
λ	1000	0.001-100000	dimensionless
Total PD-1 on tumor T cell	60000	3000-100000	molecule
Total PD-L1 on tumor cell	160000	1-160000	molecule
Ratio of PD-L2/PD-L1 on tumor cell	0.1	0-0.07	dimensionless
Total PD-L1 on APC	1600000	80000-2400000	molecule
Ratio of PD-L2/PD-L1 on APC	0.1	0-0.07	dimensionless
χ	100	0.001-100000	dimensionless

Table S4. Overall Response Rate

Treatments	Simulated ORR (%)	95% CI	Clinical ORR (%) (11)
Cibisatamab (TCE)	5.2	(0.0%, 19.4%)	6
Atezolizumab (aPD-L1)	8.2	(0.0%, 12.9%)	N/A
Combination Therapy	11.2	(0.0%, 24.0%)	12

Table S5. Distribution of Overall Response Rate in Bootstrapping samples

ORR range (%)	Atezolizumab	Cibisatamab	Combo
0-3	7%	18%	5%
3-6	20%	33%	17%
6-9	26%	27%	25%
9-12	23%	14%	24%
12-15	14%	5%	16%
15-18	6%	2%	8%
>18	4%	1%	5%

REFERENCES

1. Jafarnejad M, Gong C, Gabrielson E, Bartelink IH, Vicini P, Wang B, et al. A Computational Model of Neoadjuvant PD-1 Inhibition in Non-Small Cell Lung Cancer. Aaps Journal. 2019;21(5).
2. Cheng XX, Veverka V, Radhakrishnan A, Waters LC, Muskett FW, Morgan SH, et al. Structure and Interactions of the Human Programmed Cell Death 1 Receptor. J Biol Chem. 2013;288(17):11771-85.
3. Mkrtichyan M, Najjar YG, Raulfs EC, Liu L, Langerman S, Guittard G, et al. B7-DC-Ig enhances vaccine effect by a novel mechanism dependent on PD-1 expression level on T cell subsets. The Journal of Immunology. 2012;189(5):2338-47.
4. Harms BD, Kearns JD, Iadevaia S, Lugovskoy AA. Understanding the role of cross-arm binding efficiency in the activity of monoclonal and multispecific therapeutic antibodies. Methods. 2014;65(1):95-104.
5. Betts A, Haddish-Berhane N, Shah DK, van der Graaf PH, Barletta F, King L, et al. A Translational Quantitative Systems Pharmacology Model for CD3 Bispecific Molecules: Application to Quantify T Cell-Mediated Tumor Cell Killing by P-Cadherin LP DART((R)). Aaps Journal. 2019;21(4).
6. Ma H, Wang H, Sové RJ, Jafarnejad M, Tsai C-H, Wang J, et al. A Quantitative Systems Pharmacology Model of T Cell Engager Applied to Solid Tumor. Aaps Journal. 2020;22(4):85.
7. Stroh M, Winter H, Marchand M, Claret L, Eppler S, Ruppel J, et al. Clinical pharmacokinetics and pharmacodynamics of atezolizumab in metastatic urothelial carcinoma. Clin Pharmacol Ther. 2017;102(2):305-12.

8. Yarchoan M, Albacker LA, Hopkins AC, Montesion M, Murugesan K, Vithayathil TT, et al. PD-L1 expression and tumor mutational burden are independent biomarkers in most cancers. Jci Insight. 2019;4(6).
9. Taube JM, Klein A, Brahmer JR, Xu HY, Pan XY, Kim JH, et al. Association of PD-1, PD-1 Ligands, and Other Features of the Tumor Immune Microenvironment with Response to Anti-PD-1 Therapy. Clin Cancer Res. 2014;20(19):5064-74.
10. Herbst RS, Soria J-C, Kowanetz M, Fine GD, Hamid O, Gordon MS, et al. Predictive correlates of response to the anti-PD-L1 antibody MPDL3280A in cancer patients. Nature. 2014;515(7528):563-7.
11. Tabernero J, Melero I, Ros W, Argiles G, Marabelle A, Rodriguez-Ruiz ME, et al. Phase Ia and lb studies of the novel carcinoembryonic antigen (CEA) 1-cell bispecific (CEA CD3 TCB) antibody as a single agent and in combination with atezolizumab: Preliminary efficacy and safety in patients with metastatic colorectal cancer (mCRC). J Clin Oncol. 2017;35.
