# Supplementary material for: Combination therapy with T cell engager and PD-L1 blockade enhances the antitumor potency of T cells as predicted by a QSP model
Source: J Immunother Cancer. 2020 Aug 27;8(2):e001141. doi: 10.1136/jitc-2020-001141 (PMC7454244; doi:10.1136/jitc-2020-001141)
Supplement: Supplementary data [file jitc-2020-001141supp002.pdf]

BMJ Publishing Group Limited (BMJ) disclaims all liability and responsibility arising from any reliance placed on this supplemental material which has been supplied by the author(s)


```
<?xml version="1.0" encoding="UTF-8"?>
<sbml xmlns="http://www.sbml.org/sbml/level2/version4" level="2"
version="4">
  <annotation>
    <SimBiology xmlns="http://www.mathworks.com">
      <Version Major="5" Minor="8" Point="1"/>
    </SimBiology>
  </annotation>
  <model id="mw80ebaeaa_3ca6_4ada_b754_b7cf9aeece5a" name="Immune
Oncology Model">
    <listOfUnitDefinitions>
      <unitDefinition id="MWBUILTINUNIT_liter" name="liter">
        <listOfUnits>
          <unit kind="metre" exponent="3"/>
          <unit kind="dimensionless" multiplier="0.001"/>
        </listOfUnits>
      </unitDefinition>
      <unitDefinition id="MWBUILTINPREFIX_micro_MWBUILTINUNIT_liter"
name="microliter">
        <listOfUnits>
          <unit kind="metre" exponent="3"/>
          <unit kind="dimensionless" multiplier="1e-09"/>
        </listOfUnits>
      </unitDefinition>
      <unitDefinition id="MWDERIVEDUNIT_millimeter___3"
name="millimeter^3">
        <listOfUnits>
          <unit kind="metre" exponent="3"/>
          <unit kind="dimensionless" multiplier="1e-09"/>
        </listOfUnits>
      </unitDefinition>
      <unitDefinition id="MWDERIVEDUNIT_micrometer___2"
name="micrometer^2">
        <listOfUnits>
          <unit kind="metre" exponent="2"/>
          <unit kind="dimensionless" multiplier="1e-12"/>
        </listOfUnits>
      </unitDefinition>
      <unitDefinition id="MWUSERUNIT_cell" name="cell">
        <listOfUnits>
          <unit kind="mole" exponent="1"
multiplier="1.66053872801495e-24"/>
        </listOfUnits>
      </unitDefinition>
      <unitDefinition id="MWBUILTINUNIT_molecule" name="molecule">
        <listOfUnits>
          <unit kind="mole" exponent="1"
multiplier="1.66053872801495e-24"/>
        </listOfUnits>
      </unitDefinition>
      <unitDefinition id="MWDERIVEDUNIT_1__day" name="1/day">
        <listOfUnits>
```

```
          <unit kind="second" exponent="-1"/>
          <unit kind="dimensionless" multiplier="1.15740740740741e-
05"/>
        </listOfUnits>
      </unitDefinition>
      <unitDefinition id="MWBUILTINUNIT_day" name="day">
        <listOfUnits>
          <unit kind="second" exponent="1" multiplier="86400"/>
        </listOfUnits>
      </unitDefinition>
      <unitDefinition id="MWDERIVEDUNIT_micrometer___3__cell"
name="micrometer^3/cell">
        <listOfUnits>
          <unit kind="metre" exponent="3"/>
          <unit kind="mole" exponent="-1"/>
          <unit kind="dimensionless" multiplier="602214.199"/>
        </listOfUnits>
      </unitDefinition>
      <unitDefinition id="MWDERIVEDUNIT_cell__day" name="cell/day">
        <listOfUnits>
          <unit kind="mole" exponent="1"/>
          <unit kind="second" exponent="-1"/>
          <unit kind="dimensionless" multiplier="1.92191982409137e-
29"/>
        </listOfUnits>
      </unitDefinition>
      <unitDefinition id="MWBUILTINPREFIX_centi_MWBUILTINUNIT_meter"
name="centimeter">
        <listOfUnits>
          <unit kind="metre" exponent="1" multiplier="0.01"/>
        </listOfUnits>
      </unitDefinition>
      <unitDefinition id="MWDERIVEDUNIT_1__minute" name="1/minute">
        <listOfUnits>
          <unit kind="second" exponent="-1"/>
          <unit kind="dimensionless" multiplier="0.0166666666666667"/>
        </listOfUnits>
      </unitDefinition>
      <unitDefinition id="MWDERIVEDUNIT_1__centimeter___3_minute"
name="1/(centimeter^3*minute)">
        <listOfUnits>
          <unit kind="metre" exponent="-3"/>
          <unit kind="second" exponent="-1"/>
          <unit kind="dimensionless" multiplier="16666.6666666667"/>
        </listOfUnits>
      </unitDefinition>
      <unitDefinition id="MWDERIVEDUNIT_nanomole__cell__hour"
name="nanomole/cell/hour">
        <listOfUnits>
          <unit kind="second" exponent="-1"/>
          <unit kind="dimensionless" multiplier="167281721944.444"/>
        </listOfUnits>
```

```
      </unitDefinition>
      <unitDefinition id="MWBUILTINPREFIX_nano_MWBUILTINUNIT_molarity"
name="nanomolarity">
        <listOfUnits>
          <unit kind="metre" exponent="-3"/>
          <unit kind="mole" exponent="1"/>
          <unit kind="dimensionless" multiplier="1e-06"/>
        </listOfUnits>
      </unitDefinition>
      <unitDefinition id="MWDERIVEDUNIT_cell__milliliter"
name="cell/milliliter">
        <listOfUnits>
          <unit kind="metre" exponent="-3"/>
          <unit kind="mole" exponent="1"/>
          <unit kind="dimensionless" multiplier="1.66053872801495e-
18"/>
        </listOfUnits>
      </unitDefinition>
      <unitDefinition id="MWBUILTINUNIT_molarity" name="molarity">
        <listOfUnits>
          <unit kind="metre" exponent="-3"/>
          <unit kind="mole" exponent="1"/>
          <unit kind="dimensionless" multiplier="1000"/>
        </listOfUnits>
      </unitDefinition>
      <unitDefinition id="MWDERIVEDUNIT_mole__cell" name="mole/cell">
        <listOfUnits>
          <unit kind="dimensionless" exponent="1"
multiplier="6.02214199e+23"/>
        </listOfUnits>
      </unitDefinition>
      <unitDefinition id="MWDERIVEDUNIT_1__day__cell"
name="1/day/cell">
        <listOfUnits>
          <unit kind="mole" exponent="-1"/>
          <unit kind="second" exponent="-1"/>
          <unit kind="dimensionless"
multiplier="6.97007174768519e+18"/>
        </listOfUnits>
      </unitDefinition>
      <unitDefinition id="MWDERIVEDUNIT_1__day__molarity"
name="1/day/molarity">
        <listOfUnits>
          <unit kind="metre" exponent="3"/>
          <unit kind="mole" exponent="-1"/>
          <unit kind="second" exponent="-1"/>
          <unit kind="dimensionless" multiplier="1.15740740740741e-
08"/>
        </listOfUnits>
      </unitDefinition>
      <unitDefinition id="MWDERIVEDUNIT_molecule__micrometer___2"
name="molecule/micrometer^2">
```

BMJ Publishing Group Limited (BMJ) disclaims all liability and responsibility arising from any reliance placed on this supplemental material which has been supplied by the author(s)


```
        <listOfUnits>
          <unit kind="metre" exponent="-2"/>
          <unit kind="mole" exponent="1"/>
          <unit kind="dimensionless" multiplier="1.66053872801495e-
12"/>
        </listOfUnits>
      </unitDefinition>
      <unitDefinition id="MWDERIVEDUNIT_molarity__cell"
name="molarity/cell">
        <listOfUnits>
          <unit kind="metre" exponent="-3"/>
          <unit kind="dimensionless" multiplier="6.02214199e+26"/>
        </listOfUnits>
      </unitDefinition>
      <unitDefinition
id="MWDERIVEDUNIT_1__second_molecule__micrometer___2"
name="1/(second*molecule/micrometer^2)">
        <listOfUnits>
          <unit kind="metre" exponent="2"/>
          <unit kind="mole" exponent="-1"/>
          <unit kind="second" exponent="-1"/>
          <unit kind="dimensionless" multiplier="602214199000"/>
        </listOfUnits>
      </unitDefinition>
      <unitDefinition id="MWDERIVEDUNIT_1__second" name="1/second">
        <listOfUnits>
          <unit kind="second" exponent="-1"/>
          <unit kind="dimensionless" multiplier="1"/>
        </listOfUnits>
      </unitDefinition>
      <unitDefinition
id="MWDERIVEDUNIT_1__micromolarity_nanometer_second"
name="1/(micromolarity*nanometer*second)">
        <listOfUnits>
          <unit kind="metre" exponent="2"/>
          <unit kind="mole" exponent="-1"/>
          <unit kind="second" exponent="-1"/>
          <unit kind="dimensionless" multiplier="1000000000000"/>
        </listOfUnits>
      </unitDefinition>
      <unitDefinition id="MWDERIVEDUNIT_milliliter__second"
name="milliliter/second">
        <listOfUnits>
          <unit kind="metre" exponent="3"/>
          <unit kind="second" exponent="-1"/>
          <unit kind="dimensionless" multiplier="1e-06"/>
        </listOfUnits>
      </unitDefinition>
      <unitDefinition id="MWDERIVEDUNIT_1__hour" name="1/hour">
        <listOfUnits>
          <unit kind="second" exponent="-1"/>
```

```
          <unit kind="dimensionless"
multiplier="0.000277777777777778"/>
        </listOfUnits>
      </unitDefinition>
      <unitDefinition id="MWDERIVEDUNIT_1__molarity_second"
name="1/(molarity*second)">
        <listOfUnits>
          <unit kind="metre" exponent="3"/>
          <unit kind="mole" exponent="-1"/>
          <unit kind="second" exponent="-1"/>
          <unit kind="dimensionless" multiplier="0.001"/>
        </listOfUnits>
      </unitDefinition>
      <unitDefinition id="MWDERIVEDUNIT_1__nanometer"
name="1/nanometer">
        <listOfUnits>
          <unit kind="metre" exponent="-1"/>
          <unit kind="dimensionless" multiplier="1000000000"/>
        </listOfUnits>
      </unitDefinition>
      <unitDefinition id="MWBUILTINPREFIX_nano_MWBUILTINUNIT_meter"
name="nanometer">
        <listOfUnits>
          <unit kind="metre" exponent="1" multiplier="1e-09"/>
        </listOfUnits>
      </unitDefinition>
      <unitDefinition id="MWDERIVEDUNIT_1__molarity_nanometer_second"
name="1/(molarity*nanometer*second)">
        <listOfUnits>
          <unit kind="metre" exponent="2"/>
          <unit kind="mole" exponent="-1"/>
          <unit kind="second" exponent="-1"/>
          <unit kind="dimensionless" multiplier="1000000"/>
        </listOfUnits>
      </unitDefinition>
    </listOfUnitDefinitions>
    <listOfCompartments>
      <compartment id="mw8cfbefd5_8b10_411d_9d84_300692817ff2"
name="V_C" spatialDimensions="3" size="5" units="MWBUILTINUNIT_liter"
constant="true">
        <notes>
          <body xmlns="http://www.w3.org/1999/xhtml">Central
compartment (C) Central compartment volume</body>
        </notes>
      </compartment>
      <compartment id="mwfa3bc17c_3f2b_4900_afd9_70a9e6afc56c"
name="V_P" spatialDimensions="3" size="60" units="MWBUILTINUNIT_liter"
constant="true">
        <notes>
          <body xmlns="http://www.w3.org/1999/xhtml">Peripheral
compartment (P) Peripheral compartment volume</body>
        </notes>
```

```
      </compartment>
      <compartment id="mw175e8610_7578_45f5_80bb_7b61e6e241ab"
name="V_T" spatialDimensions="3" size="0.001"
units="MWBUILTINPREFIX_micro_MWBUILTINUNIT_liter" constant="false">
        <notes>
          <body xmlns="http://www.w3.org/1999/xhtml">Tumor compartment
(T) Cancer-free Tumour compartment volume</body>
        </notes>
      </compartment>
      <compartment id="mwb4901521_e24a_4f39_acff_107e3522faa9"
name="V_LN" spatialDimensions="3" size="1374.44678594553"
units="MWDERIVEDUNIT_millimeter___3" constant="true">
        <notes>
          <body xmlns="http://www.w3.org/1999/xhtml">Lymph node (LN)
compartment volume Lymph Node compartment volume</body>
        </notes>
      </compartment>
      <compartment id="mwf681189d_0fc1_4538_a15f_815606c049a6"
name="V_e" spatialDimensions="3" size="4e-16" units="litre"
constant="true">
        <notes>
          <body xmlns="http://www.w3.org/1999/xhtml">APC endosomal
comparment Endosomal Volume</body>
        </notes>
      </compartment>
      <compartment id="mwc9c2eea0_d90b_4a89_901f_6f5f9de09f35"
name="A_e" spatialDimensions="2" size="15"
units="MWDERIVEDUNIT_micrometer___2" constant="true">
        <notes>
          <body xmlns="http://www.w3.org/1999/xhtml">APC endosomal
surface comparment Endosomal Surface Area</body>
        </notes>
      </compartment>
      <compartment id="mwa56ed94a_0a50_45aa_b713_70c4c2665228"
name="A_s" spatialDimensions="2" size="900"
units="MWDERIVEDUNIT_micrometer___2" constant="true">
        <notes>
          <body xmlns="http://www.w3.org/1999/xhtml">APC surface
comparment APC Surface Area</body>
        </notes>
      </compartment>
      <compartment id="mw9a8fe99b_34f3_48b8_9432_7de459c65052"
name="syn_T1_C1" spatialDimensions="2" size="37.8"
units="MWDERIVEDUNIT_micrometer___2" constant="true">
        <notes>
          <body xmlns="http://www.w3.org/1999/xhtml">synapse
comparment between T1 and C1 Synapse Surface Area</body>
        </notes>
      </compartment>
      <compartment id="mwaad74090_4b37_47eb_b845_2f28d96c0e82"
name="syn_T1_APC" spatialDimensions="2" size="37.8"
units="MWDERIVEDUNIT_micrometer___2" constant="true">
```

```
        <notes>
          <body xmlns="http://www.w3.org/1999/xhtml">synapse
comparment between T1 and APC Synapse Surface Area</body>
        </notes>
      </compartment>
      <compartment id="mw0305c0ed_ac99_469b_8d32_3127d022edf9"
name="TCEsyn_T1_C1" spatialDimensions="2" size="75.6"
units="MWDERIVEDUNIT_micrometer___2" constant="true">
        <notes>
          <body xmlns="http://www.w3.org/1999/xhtml">synapse
comparment between T1 and C1 (Janssen 2007)</body>
        </notes>
      </compartment>
      <compartment id="mwfc416eeb_b169_4568_9e44_cab5996f5ee2"
name="TCEsyn_T0_C1" spatialDimensions="2" size="75.6"
units="MWDERIVEDUNIT_micrometer___2" constant="true">
        <notes>
          <body xmlns="http://www.w3.org/1999/xhtml">synapse
comparment between T0 and C1 (Janssen 2007)</body>
        </notes>
      </compartment>
    </listOfCompartments>
    <listOfSpecies>
      <species id="mwa04d3906_d300_4e70_bbc1_fb52680cd5d3" name="T1"
compartment="mw8cfbefd5_8b10_411d_9d84_300692817ff2" initialAmount="0"
substanceUnits="MWUSERUNIT_cell" hasOnlySubstanceUnits="true"
boundaryCondition="false" constant="false">
        <notes>
          <body xmlns="http://www.w3.org/1999/xhtml">Number of T1
cells in the central compartment</body>
        </notes>
      </species>
      <species id="mw2f881f74_14f8_45be_ac4a_bb4e1e3ede99" name="T0"
compartment="mw8cfbefd5_8b10_411d_9d84_300692817ff2" initialAmount="0"
substanceUnits="MWUSERUNIT_cell" hasOnlySubstanceUnits="true"
boundaryCondition="false" constant="false">
        <notes>
          <body xmlns="http://www.w3.org/1999/xhtml">Number of T0
cells in the central compartment</body>
        </notes>
      </species>
      <species id="mw700cf3ed_1735_4bbc_870e_8572cc73d50a"
name="atezolizumab"
compartment="mw8cfbefd5_8b10_411d_9d84_300692817ff2"
initialConcentration="0" substanceUnits="mole"
hasOnlySubstanceUnits="false" boundaryCondition="false"
constant="false">
        <notes>
          <body xmlns="http://www.w3.org/1999/xhtml">Concentration of
atezolizumab in central compartment</body>
        </notes>
        <annotation>
```

```
          <SimBiology xmlns="http://www.mathworks.com">
            <Unit Numerator="mole" Denominator="liter"/>
          </SimBiology>
        </annotation>
      </species>
      <species id="mw979043c1_63df_42b5_be8a_697b7f2b0604"
name="cibis" compartment="mw8cfbefd5_8b10_411d_9d84_300692817ff2"
initialConcentration="0" substanceUnits="mole"
hasOnlySubstanceUnits="false" boundaryCondition="false"
constant="false">
        <notes>
          <body xmlns="http://www.w3.org/1999/xhtml">Concentration of
cibis in central compartment</body>
        </notes>
        <annotation>
          <SimBiology xmlns="http://www.mathworks.com">
            <Unit Numerator="mole" Denominator="liter"/>
          </SimBiology>
        </annotation>
      </species>
      <species id="mw2c87cd24_8cfc_4eb0_8b60_5565ba85c240" name="T1"
compartment="mwfa3bc17c_3f2b_4900_afd9_70a9e6afc56c" initialAmount="0"
substanceUnits="MWUSERUNIT_cell" hasOnlySubstanceUnits="true"
boundaryCondition="false" constant="false">
        <notes>
          <body xmlns="http://www.w3.org/1999/xhtml">Number of T1
cells in the peripheral compartment</body>
        </notes>
      </species>
      <species id="mw56b94d8b_7702_49a1_a07a_62a53c209830" name="T0"
compartment="mwfa3bc17c_3f2b_4900_afd9_70a9e6afc56c" initialAmount="0"
substanceUnits="MWUSERUNIT_cell" hasOnlySubstanceUnits="true"
boundaryCondition="false" constant="false">
        <notes>
          <body xmlns="http://www.w3.org/1999/xhtml">Number of T0
cells in the peripheral compartment</body>
        </notes>
      </species>
      <species id="mw5581393f_3eaf_48a1_9e8b_e42b2557f0d9"
name="atezolizumab"
compartment="mwfa3bc17c_3f2b_4900_afd9_70a9e6afc56c"
initialConcentration="0" substanceUnits="mole"
hasOnlySubstanceUnits="false" boundaryCondition="false"
constant="false">
        <notes>
          <body xmlns="http://www.w3.org/1999/xhtml">Concentration of
atezolizumab in peripheral compartment</body>
        </notes>
        <annotation>
          <SimBiology xmlns="http://www.mathworks.com">
            <Unit Numerator="mole" Denominator="liter"/>
          </SimBiology>
```

```
        </annotation>
      </species>
      <species id="mwfa76d65a_154f_40ed_b9e1_0406e01c1120"
name="cibis" compartment="mwfa3bc17c_3f2b_4900_afd9_70a9e6afc56c"
initialConcentration="0" substanceUnits="mole"
hasOnlySubstanceUnits="false" boundaryCondition="false"
constant="false">
        <notes>
          <body xmlns="http://www.w3.org/1999/xhtml">Concentration of
cibis in peripheral compartment</body>
        </notes>
        <annotation>
          <SimBiology xmlns="http://www.mathworks.com">
            <Unit Numerator="mole" Denominator="liter"/>
          </SimBiology>
        </annotation>
      </species>
      <species id="mwdf5f1168_f5b1_4306_8f6d_418a6021e921" name="C_x"
compartment="mw175e8610_7578_45f5_80bb_7b61e6e241ab" initialAmount="0"
substanceUnits="MWUSERUNIT_cell" hasOnlySubstanceUnits="true"
boundaryCondition="false" constant="false">
        <notes>
          <body xmlns="http://www.w3.org/1999/xhtml">Dead cancer cells
in the tumour compartment</body>
        </notes>
      </species>
      <species id="mw088a157f_05c9_4257_a674_1b5bb4ee465b"
name="T_exh" compartment="mw175e8610_7578_45f5_80bb_7b61e6e241ab"
initialAmount="0" substanceUnits="MWUSERUNIT_cell"
hasOnlySubstanceUnits="true" boundaryCondition="false"
constant="false">
        <notes>
          <body xmlns="http://www.w3.org/1999/xhtml">Exhausted T
cells</body>
        </notes>
      </species>
      <species id="mw2aafd721_12db_4909_b572_82abf04c578d" name="C1"
compartment="mw175e8610_7578_45f5_80bb_7b61e6e241ab" initialAmount="1"
substanceUnits="MWUSERUNIT_cell" hasOnlySubstanceUnits="true"
boundaryCondition="false" constant="false">
        <notes>
          <body xmlns="http://www.w3.org/1999/xhtml">Number of cancer
cells in tumour</body>
        </notes>
      </species>
      <species id="mw66344d11_136c_4af3_9f9a_cc5c3035331c" name="T1"
compartment="mw175e8610_7578_45f5_80bb_7b61e6e241ab" initialAmount="0"
substanceUnits="MWUSERUNIT_cell" hasOnlySubstanceUnits="true"
boundaryCondition="false" constant="false">
        <notes>
          <body xmlns="http://www.w3.org/1999/xhtml">Number of T1
cells in the tumour compartment</body>
```

```
        </notes>
      </species>
      <species id="mwa5fd10b1_5a9e_41d2_a246_260c6fc1dbf5" name="T0"
compartment="mw175e8610_7578_45f5_80bb_7b61e6e241ab" initialAmount="0"
substanceUnits="MWUSERUNIT_cell" hasOnlySubstanceUnits="true"
boundaryCondition="false" constant="false">
        <notes>
          <body xmlns="http://www.w3.org/1999/xhtml">Number of T0
cells in the tumour compartment</body>
        </notes>
      </species>
      <species id="mwf53e0b71_eca1_413f_9604_8297474f789f" name="APC"
compartment="mw175e8610_7578_45f5_80bb_7b61e6e241ab" initialAmount="0"
substanceUnits="MWUSERUNIT_cell" hasOnlySubstanceUnits="true"
boundaryCondition="false" constant="false">
        <notes>
          <body xmlns="http://www.w3.org/1999/xhtml">Number of naive
antigen presenting cells in the tumour</body>
        </notes>
      </species>
      <species id="mw40d0c8ab_6e98_45f5_a2d7_4f4167cfc589" name="mAPC"
compartment="mw175e8610_7578_45f5_80bb_7b61e6e241ab" initialAmount="0"
substanceUnits="MWUSERUNIT_cell" hasOnlySubstanceUnits="true"
boundaryCondition="false" constant="false">
        <notes>
          <body xmlns="http://www.w3.org/1999/xhtml">Number of mature
antigen presenting cells in the tumour</body>
        </notes>
      </species>
      <species id="mw1a0b99a8_2ed5_48b7_9014_a1041ffeebb3" name="c"
compartment="mw175e8610_7578_45f5_80bb_7b61e6e241ab"
initialConcentration="0" substanceUnits="mole"
hasOnlySubstanceUnits="false" boundaryCondition="false"
constant="false">
        <notes>
          <body xmlns="http://www.w3.org/1999/xhtml">Concentration of
maturation cytokines in the tumour</body>
        </notes>
        <annotation>
          <SimBiology xmlns="http://www.mathworks.com">
            <Unit Numerator="mole" Denominator="liter"/>
          </SimBiology>
        </annotation>
      </species>
      <species id="mw59118247_974d_4558_88d8_f2e55f289d1a"
name="atezolizumab"
compartment="mw175e8610_7578_45f5_80bb_7b61e6e241ab"
initialConcentration="0" substanceUnits="mole"
hasOnlySubstanceUnits="false" boundaryCondition="false"
constant="false">
        <notes>
```

```
          <body xmlns="http://www.w3.org/1999/xhtml">Concentration of
atezolizumab in tumour compartment</body>
        </notes>
        <annotation>
          <SimBiology xmlns="http://www.mathworks.com">
            <Unit Numerator="mole" Denominator="liter"/>
          </SimBiology>
        </annotation>
      </species>
      <species id="mw9c0222d8_0066_4f7c_8803_f18575631cfe"
name="cibis" compartment="mw175e8610_7578_45f5_80bb_7b61e6e241ab"
initialConcentration="0" substanceUnits="mole"
hasOnlySubstanceUnits="false" boundaryCondition="false"
constant="false">
        <notes>
          <body xmlns="http://www.w3.org/1999/xhtml">Concentration of
cibis in tumour compartment</body>
        </notes>
        <annotation>
          <SimBiology xmlns="http://www.mathworks.com">
            <Unit Numerator="mole" Denominator="liter"/>
          </SimBiology>
        </annotation>
      </species>
      <species id="mwaf05be2a_412f_48e6_8b06_f5d247ba6883" name="nT1"
compartment="mwb4901521_e24a_4f39_acff_107e3522faa9" initialAmount="0"
substanceUnits="MWUSERUNIT_cell" hasOnlySubstanceUnits="true"
boundaryCondition="false" constant="false">
        <notes>
          <body xmlns="http://www.w3.org/1999/xhtml">Number of naive
T1 cells in the lymph node</body>
        </notes>
      </species>
      <species id="mwf317e8cb_4357_45f5_b8c5_961986d1cb12" name="aT1"
compartment="mwb4901521_e24a_4f39_acff_107e3522faa9" initialAmount="0"
substanceUnits="MWUSERUNIT_cell" hasOnlySubstanceUnits="true"
boundaryCondition="false" constant="false">
        <notes>
          <body xmlns="http://www.w3.org/1999/xhtml">Number of
activated T1 cells in the lymph node</body>
        </notes>
      </species>
      <species id="mw975fec59_e535_46d1_98b4_82ad5cb2c176" name="T1"
compartment="mwb4901521_e24a_4f39_acff_107e3522faa9" initialAmount="0"
substanceUnits="MWUSERUNIT_cell" hasOnlySubstanceUnits="true"
boundaryCondition="false" constant="false">
        <notes>
          <body xmlns="http://www.w3.org/1999/xhtml">Number of T1
cells in the lymph node compartment</body>
        </notes>
      </species>
```

```
      <species id="mw479632c6_8f17_47aa_b429_216752f11c51" name="IL2"
compartment="mwb4901521_e24a_4f39_acff_107e3522faa9"
initialConcentration="1e-24" substanceUnits="mole"
hasOnlySubstanceUnits="false" boundaryCondition="false"
constant="false">
        <notes>
          <body xmlns="http://www.w3.org/1999/xhtml">Concentration of
IL2 in the lymph node compartment</body>
        </notes>
        <annotation>
          <SimBiology xmlns="http://www.mathworks.com">
            <Unit Numerator="mole" Denominator="liter"/>
          </SimBiology>
        </annotation>
      </species>
      <species id="mw8607693c_2da9_4dd0_9167_ab2793ec2f3a" name="nT0"
compartment="mwb4901521_e24a_4f39_acff_107e3522faa9" initialAmount="0"
substanceUnits="MWUSERUNIT_cell" hasOnlySubstanceUnits="true"
boundaryCondition="false" constant="false">
        <notes>
          <body xmlns="http://www.w3.org/1999/xhtml">Number of naive
T0 cells in the lymph node</body>
        </notes>
      </species>
      <species id="mw26ca4282_20b3_4f9b_8ce4_58824cec85fc" name="aT0"
compartment="mwb4901521_e24a_4f39_acff_107e3522faa9" initialAmount="0"
substanceUnits="MWUSERUNIT_cell" hasOnlySubstanceUnits="true"
boundaryCondition="false" constant="false">
        <notes>
          <body xmlns="http://www.w3.org/1999/xhtml">Number of
activated T0 cells in the lymph node</body>
        </notes>
      </species>
      <species id="mwd3fc3e45_040b_42b7_b9c1_0f60a875237c" name="T0"
compartment="mwb4901521_e24a_4f39_acff_107e3522faa9" initialAmount="0"
substanceUnits="MWUSERUNIT_cell" hasOnlySubstanceUnits="true"
boundaryCondition="false" constant="false">
        <notes>
          <body xmlns="http://www.w3.org/1999/xhtml">Number of T0
cells in the lymph node compartment</body>
        </notes>
      </species>
      <species id="mw0f3e4986_c5c0_4e85_8f07_03bf0ed91f2b" name="APC"
compartment="mwb4901521_e24a_4f39_acff_107e3522faa9" initialAmount="0"
substanceUnits="MWUSERUNIT_cell" hasOnlySubstanceUnits="true"
boundaryCondition="false" constant="false">
        <notes>
          <body xmlns="http://www.w3.org/1999/xhtml">Number of naive
antigen presenting cells in the lymph node</body>
        </notes>
      </species>
```

```
      <species id="mw507424c1_49c9_4a06_8493_4e61e9f65e52" name="mAPC"
compartment="mwb4901521_e24a_4f39_acff_107e3522faa9" initialAmount="0"
substanceUnits="MWUSERUNIT_cell" hasOnlySubstanceUnits="true"
boundaryCondition="false" constant="false">
        <notes>
          <body xmlns="http://www.w3.org/1999/xhtml">Number of mature
antigen presenting cells in the lymph node</body>
        </notes>
      </species>
      <species id="mwfd9cdc6d_ef96_441b_9ef2_89e3ce96bf71" name="P0"
compartment="mwb4901521_e24a_4f39_acff_107e3522faa9"
initialConcentration="1e-24" substanceUnits="mole"
hasOnlySubstanceUnits="false" boundaryCondition="false"
constant="false">
        <notes>
          <body xmlns="http://www.w3.org/1999/xhtml">Concentration of
free antigen (P0) in the LN compartment</body>
        </notes>
        <annotation>
          <SimBiology xmlns="http://www.mathworks.com">
            <Unit Numerator="mole" Denominator="liter"/>
          </SimBiology>
        </annotation>
      </species>
      <species id="mw38def0d7_03a6_42e4_898f_2b834be9e4a0" name="P1"
compartment="mwb4901521_e24a_4f39_acff_107e3522faa9"
initialConcentration="1e-24" substanceUnits="mole"
hasOnlySubstanceUnits="false" boundaryCondition="false"
constant="false">
        <notes>
          <body xmlns="http://www.w3.org/1999/xhtml">Concentration of
free antigen (P1) in the LN compartment</body>
        </notes>
        <annotation>
          <SimBiology xmlns="http://www.mathworks.com">
            <Unit Numerator="mole" Denominator="liter"/>
          </SimBiology>
        </annotation>
      </species>
      <species id="mw2a165e3b_dfa5_4d73_9af6_2fd6099e47e3"
name="atezolizumab"
compartment="mwb4901521_e24a_4f39_acff_107e3522faa9"
initialConcentration="0" substanceUnits="mole"
hasOnlySubstanceUnits="false" boundaryCondition="false"
constant="false">
        <notes>
          <body xmlns="http://www.w3.org/1999/xhtml">Concentration of
atezolizumab in lymph node compartment</body>
        </notes>
        <annotation>
          <SimBiology xmlns="http://www.mathworks.com">
            <Unit Numerator="mole" Denominator="liter"/>
```

```
          </SimBiology>
        </annotation>
      </species>
      <species id="mw98d3c290_6a02_4140_b562_ca1a3cb39c99"
name="cibis" compartment="mwb4901521_e24a_4f39_acff_107e3522faa9"
initialConcentration="0" substanceUnits="mole"
hasOnlySubstanceUnits="false" boundaryCondition="false"
constant="false">
        <notes>
          <body xmlns="http://www.w3.org/1999/xhtml">Concentration of
cibis in lymph node compartment</body>
        </notes>
        <annotation>
          <SimBiology xmlns="http://www.mathworks.com">
            <Unit Numerator="mole" Denominator="liter"/>
          </SimBiology>
        </annotation>
      </species>
      <species id="mw3cfa9342_80e4_4ad5_a763_147d1db83eef" name="P0"
compartment="mwf681189d_0fc1_4538_a15f_815606c049a6"
initialConcentration="1e-18" substanceUnits="mole"
hasOnlySubstanceUnits="false" boundaryCondition="false"
constant="false">
        <notes>
          <body xmlns="http://www.w3.org/1999/xhtml">Concentration of
antigen P0 in the APC endosomes</body>
        </notes>
        <annotation>
          <SimBiology xmlns="http://www.mathworks.com">
            <Unit Numerator="mole" Denominator="liter"/>
          </SimBiology>
        </annotation>
      </species>
      <species id="mw833b4186_5415_4464_ba45_e286d775edb5" name="p0"
compartment="mwf681189d_0fc1_4538_a15f_815606c049a6"
initialConcentration="1e-18" substanceUnits="mole"
hasOnlySubstanceUnits="false" boundaryCondition="false"
constant="false">
        <notes>
          <body xmlns="http://www.w3.org/1999/xhtml">Concentration of
epitope P0 in the APC endosomes</body>
        </notes>
        <annotation>
          <SimBiology xmlns="http://www.mathworks.com">
            <Unit Numerator="mole" Denominator="liter"/>
          </SimBiology>
        </annotation>
      </species>
      <species id="mw53b8f0a1_9dae_4ac6_be5b_0b3e78208e34" name="P1"
compartment="mwf681189d_0fc1_4538_a15f_815606c049a6"
initialConcentration="1e-18" substanceUnits="mole"
```

```
hasOnlySubstanceUnits="false" boundaryCondition="false"
constant="false">
        <notes>
          <body xmlns="http://www.w3.org/1999/xhtml">Concentration of
antigen P1 in the APC endosomes</body>
        </notes>
        <annotation>
          <SimBiology xmlns="http://www.mathworks.com">
            <Unit Numerator="mole" Denominator="liter"/>
          </SimBiology>
        </annotation>
      </species>
      <species id="mwdd1d892a_1451_4309_955b_e886b7583ba5" name="p1"
compartment="mwf681189d_0fc1_4538_a15f_815606c049a6"
initialConcentration="1e-18" substanceUnits="mole"
hasOnlySubstanceUnits="false" boundaryCondition="false"
constant="false">
        <notes>
          <body xmlns="http://www.w3.org/1999/xhtml">Concentration of
epitope P1 in the APC endosomes</body>
        </notes>
        <annotation>
          <SimBiology xmlns="http://www.mathworks.com">
            <Unit Numerator="mole" Denominator="liter"/>
          </SimBiology>
        </annotation>
      </species>
      <species id="mw942caa29_eb06_4495_9672_6335f8011e75" name="M1"
compartment="mwc9c2eea0_d90b_4a89_901f_6f5f9de09f35"
initialConcentration="2185.79234972678"
substanceUnits="MWBUILTINUNIT_molecule" hasOnlySubstanceUnits="false"
boundaryCondition="false" constant="false">
        <notes>
          <body xmlns="http://www.w3.org/1999/xhtml">Amount of MHC per
area on the cell surface</body>
        </notes>
        <annotation>
          <SimBiology xmlns="http://www.mathworks.com">
            <Unit Numerator="molecule" Denominator="micrometer^2"/>
          </SimBiology>
        </annotation>
      </species>
      <species id="mwae431b93_56a6_4720_bd61_fb993f5e5c47" name="M1p0"
compartment="mwc9c2eea0_d90b_4a89_901f_6f5f9de09f35"
initialConcentration="1e-06" substanceUnits="MWBUILTINUNIT_molecule"
hasOnlySubstanceUnits="false" boundaryCondition="false"
constant="false">
        <notes>
          <body xmlns="http://www.w3.org/1999/xhtml">Antigen-MHC
complex</body>
        </notes>
        <annotation>
```

```
          <SimBiology xmlns="http://www.mathworks.com">
            <Unit Numerator="molecule" Denominator="micrometer^2"/>
          </SimBiology>
        </annotation>
      </species>
      <species id="mw0e63cb09_149e_4a8e_b596_9e21ce9234cd" name="M1p1"
compartment="mwc9c2eea0_d90b_4a89_901f_6f5f9de09f35"
initialConcentration="1e-06" substanceUnits="MWBUILTINUNIT_molecule"
hasOnlySubstanceUnits="false" boundaryCondition="false"
constant="false">
        <notes>
          <body xmlns="http://www.w3.org/1999/xhtml">Antigen-MHC
complex</body>
        </notes>
        <annotation>
          <SimBiology xmlns="http://www.mathworks.com">
            <Unit Numerator="molecule" Denominator="micrometer^2"/>
          </SimBiology>
        </annotation>
      </species>
      <species id="mw832e95af_83ea_46bd_9427_ce8eae60b699" name="M1"
compartment="mwa56ed94a_0a50_45aa_b713_70c4c2665228"
initialConcentration="1e-06" substanceUnits="MWBUILTINUNIT_molecule"
hasOnlySubstanceUnits="false" boundaryCondition="false"
constant="false">
        <annotation>
          <SimBiology xmlns="http://www.mathworks.com">
            <Unit Numerator="molecule" Denominator="micrometer^2"/>
          </SimBiology>
        </annotation>
      </species>
      <species id="mwd1db5e41_695c_4769_b3d8_4151bf3a44d0" name="M1p0"
compartment="mwa56ed94a_0a50_45aa_b713_70c4c2665228"
initialConcentration="1e-06" substanceUnits="MWBUILTINUNIT_molecule"
hasOnlySubstanceUnits="false" boundaryCondition="false"
constant="false">
        <notes>
          <body xmlns="http://www.w3.org/1999/xhtml">Antigen-MHC
complex</body>
        </notes>
        <annotation>
          <SimBiology xmlns="http://www.mathworks.com">
            <Unit Numerator="molecule" Denominator="micrometer^2"/>
          </SimBiology>
        </annotation>
      </species>
      <species id="mwf7546543_1576_4ff3_a1d2_09f9d9d8f508" name="M1p1"
compartment="mwa56ed94a_0a50_45aa_b713_70c4c2665228"
initialConcentration="1e-06" substanceUnits="MWBUILTINUNIT_molecule"
hasOnlySubstanceUnits="false" boundaryCondition="false"
constant="false">
        <notes>
```

```
          &lt;body xmlns="http://www.w3.org/1999/xhtml"&gt;Antigen-MHC
complex&lt;/body&gt;
        &lt;/notes&gt;
        &lt;annotation&gt;
          &lt;SimBiology xmlns="http://www.mathworks.com"&gt;
            &lt;Unit Numerator="molecule" Denominator="micrometer^2"/&gt;
          &lt;/SimBiology&gt;
        &lt;/annotation&gt;
      &lt;/species&gt;
      <species id="mw538e3c56_061a_417d_9d7f_128d0cd23afa"
name="PD1_PDL1" compartment="mw9a8fe99b_34f3_48b8_9432_7de459c65052"
initialConcentration="0" substanceUnits="MWBUILTINUNIT_molecule"
hasOnlySubstanceUnits="false" boundaryCondition="false"
constant="false">
        &lt;notes&gt;
          &lt;body xmlns="http://www.w3.org/1999/xhtml"&gt;concentration of
PD1-PDL1 complex&lt;/body&gt;
        &lt;/notes&gt;
        &lt;annotation&gt;
          &lt;SimBiology xmlns="http://www.mathworks.com"&gt;
            &lt;Unit Numerator="molecule" Denominator="micrometer^2"/&gt;
          &lt;/SimBiology&gt;
        &lt;/annotation&gt;
      &lt;/species&gt;
      <species id="mw80d2fce8_7bcd_47a0_8857_df9df2037ea4"
name="PD1_PDL2" compartment="mw9a8fe99b_34f3_48b8_9432_7de459c65052"
initialConcentration="0" substanceUnits="MWBUILTINUNIT_molecule"
hasOnlySubstanceUnits="false" boundaryCondition="false"
constant="false">
        &lt;notes&gt;
          &lt;body xmlns="http://www.w3.org/1999/xhtml"&gt;concentration of
PD1-PDL2 complex&lt;/body&gt;
        &lt;/notes&gt;
        &lt;annotation&gt;
          &lt;SimBiology xmlns="http://www.mathworks.com"&gt;
            &lt;Unit Numerator="molecule" Denominator="micrometer^2"/&gt;
          &lt;/SimBiology&gt;
        &lt;/annotation&gt;
      &lt;/species&gt;
      <species id="mw895d97df_cb0d_44c8_9785_11b1d6282d01" name="PD1"
compartment="mw9a8fe99b_34f3_48b8_9432_7de459c65052"
initialConcentration="0" substanceUnits="MWBUILTINUNIT_molecule"
hasOnlySubstanceUnits="false" boundaryCondition="false"
constant="false">
        &lt;notes&gt;
          &lt;body xmlns="http://www.w3.org/1999/xhtml"&gt;concentration of
PD1 in synapse&lt;/body&gt;
        &lt;/notes&gt;
        &lt;annotation&gt;
          &lt;SimBiology xmlns="http://www.mathworks.com"&gt;
            &lt;Unit Numerator="molecule" Denominator="micrometer^2"/&gt;
          &lt;/SimBiology&gt;
```

```
        &lt;/annotation&gt;
      &lt;/species&gt;
      <species id="mw48d27efc_8ace_4319_b906_4465d77a46fa" name="PDL1"
compartment="mw9a8fe99b_34f3_48b8_9432_7de459c65052"
initialConcentration="0" substanceUnits="MWBUILTINUNIT_molecule"
hasOnlySubstanceUnits="false" boundaryCondition="false"
constant="false">
        &lt;notes&gt;
          &lt;body xmlns="http://www.w3.org/1999/xhtml"&gt;concentration of
PDL1 in synapse&lt;/body&gt;
        &lt;/notes&gt;
        &lt;annotation&gt;
          &lt;SimBiology xmlns="http://www.mathworks.com"&gt;
            &lt;Unit Numerator="molecule" Denominator="micrometer^2"/&gt;
          &lt;/SimBiology&gt;
        &lt;/annotation&gt;
      &lt;/species&gt;
      <species id="mw46e10e70_4f89_41d2_98d3_04c055feaf2d" name="PDL2"
compartment="mw9a8fe99b_34f3_48b8_9432_7de459c65052"
initialConcentration="0" substanceUnits="MWBUILTINUNIT_molecule"
hasOnlySubstanceUnits="false" boundaryCondition="false"
constant="false">
        &lt;notes&gt;
          &lt;body xmlns="http://www.w3.org/1999/xhtml"&gt;concentration of
PDL2 in synapse&lt;/body&gt;
        &lt;/notes&gt;
        &lt;annotation&gt;
          &lt;SimBiology xmlns="http://www.mathworks.com"&gt;
            &lt;Unit Numerator="molecule" Denominator="micrometer^2"/&gt;
          &lt;/SimBiology&gt;
        &lt;/annotation&gt;
      &lt;/species&gt;
      <species id="mw0aa710e6_b780_4cb3_a8dc_464b70d319d9"
name="PD1_aPD1" compartment="mw9a8fe99b_34f3_48b8_9432_7de459c65052"
initialConcentration="0" substanceUnits="MWBUILTINUNIT_molecule"
hasOnlySubstanceUnits="false" boundaryCondition="false"
constant="false">
        &lt;notes&gt;
          &lt;body xmlns="http://www.w3.org/1999/xhtml"&gt;concentration of
PD1-nivolumab complex&lt;/body&gt;
        &lt;/notes&gt;
        &lt;annotation&gt;
          &lt;SimBiology xmlns="http://www.mathworks.com"&gt;
            &lt;Unit Numerator="molecule" Denominator="micrometer^2"/&gt;
          &lt;/SimBiology&gt;
        &lt;/annotation&gt;
      &lt;/species&gt;
      <species id="mw0e147b6c_09cf_484f_9819_87470663749d"
name="PD1_aPD1_PD1"
compartment="mw9a8fe99b_34f3_48b8_9432_7de459c65052"
initialConcentration="0" substanceUnits="MWBUILTINUNIT_molecule"
```

```
hasOnlySubstanceUnits="false" boundaryCondition="false"
constant="false">
        &lt;notes&gt;
          &lt;body xmlns="http://www.w3.org/1999/xhtml"&gt;concentration of
PD1-nivolumab-PD1 complex&lt;/body&gt;
        &lt;/notes&gt;
        &lt;annotation&gt;
          &lt;SimBiology xmlns="http://www.mathworks.com"&gt;
            &lt;Unit Numerator="molecule" Denominator="micrometer^2"/&gt;
          &lt;/SimBiology&gt;
        &lt;/annotation&gt;
      &lt;/species&gt;
      <species id="mw9c13852d_8802_4ef2_9d33_f72a5a747dea"
name="PDL1_aPDL1" compartment="mw9a8fe99b_34f3_48b8_9432_7de459c65052"
initialConcentration="0" substanceUnits="MWBUILTINUNIT_molecule"
hasOnlySubstanceUnits="false" boundaryCondition="false"
constant="false">
        &lt;notes&gt;
          &lt;body xmlns="http://www.w3.org/1999/xhtml"&gt;concentration of
PDL1-durvalumab complex&lt;/body&gt;
        &lt;/notes&gt;
        &lt;annotation&gt;
          &lt;SimBiology xmlns="http://www.mathworks.com"&gt;
            &lt;Unit Numerator="molecule" Denominator="micrometer^2"/&gt;
          &lt;/SimBiology&gt;
        &lt;/annotation&gt;
      &lt;/species&gt;
      <species id="mwfc64755e_b1aa_411d_a1f7_8bad9fe66a96"
name="PDL1_aPDL1_PDL1"
compartment="mw9a8fe99b_34f3_48b8_9432_7de459c65052"
initialConcentration="0" substanceUnits="MWBUILTINUNIT_molecule"
hasOnlySubstanceUnits="false" boundaryCondition="false"
constant="false">
        &lt;notes&gt;
          &lt;body xmlns="http://www.w3.org/1999/xhtml"&gt;concentration of
PDL1-durvalumab-PDL1 complex&lt;/body&gt;
        &lt;/notes&gt;
        &lt;annotation&gt;
          &lt;SimBiology xmlns="http://www.mathworks.com"&gt;
            &lt;Unit Numerator="molecule" Denominator="micrometer^2"/&gt;
          &lt;/SimBiology&gt;
        &lt;/annotation&gt;
      &lt;/species&gt;
      <species id="mwc47a95ad_5c77_4a62_b9b7_cb2b8abe3260"
name="PD1_PDL1" compartment="mwaad74090_4b37_47eb_b845_2f28d96c0e82"
initialConcentration="0" substanceUnits="MWBUILTINUNIT_molecule"
hasOnlySubstanceUnits="false" boundaryCondition="false"
constant="false">
        &lt;notes&gt;
          &lt;body xmlns="http://www.w3.org/1999/xhtml"&gt;concentration of
PD1-PDL1 complex&lt;/body&gt;
        &lt;/notes&gt;
```

```
        &lt;annotation&gt;
          &lt;SimBiology xmlns="http://www.mathworks.com"&gt;
            &lt;Unit Numerator="molecule" Denominator="micrometer^2"/&gt;
          &lt;/SimBiology&gt;
        &lt;/annotation&gt;
      &lt;/species&gt;
      <species id="mw345e02b4_624f_415f_af98_e55a9f555853"
name="PD1_PDL2" compartment="mwaad74090_4b37_47eb_b845_2f28d96c0e82"
initialConcentration="0" substanceUnits="MWBUILTINUNIT_molecule"
hasOnlySubstanceUnits="false" boundaryCondition="false"
constant="false">
        &lt;notes&gt;
          &lt;body xmlns="http://www.w3.org/1999/xhtml"&gt;concentration of
PD1-PDL2 complex&lt;/body&gt;
        &lt;/notes&gt;
        &lt;annotation&gt;
          &lt;SimBiology xmlns="http://www.mathworks.com"&gt;
            &lt;Unit Numerator="molecule" Denominator="micrometer^2"/&gt;
          &lt;/SimBiology&gt;
        &lt;/annotation&gt;
      &lt;/species&gt;
      <species id="mw4d33db81_6628_421d_8c61_5ef4a4a647ad" name="PD1"
compartment="mwaad74090_4b37_47eb_b845_2f28d96c0e82"
initialConcentration="0" substanceUnits="MWBUILTINUNIT_molecule"
hasOnlySubstanceUnits="false" boundaryCondition="false"
constant="false">
        &lt;notes&gt;
          &lt;body xmlns="http://www.w3.org/1999/xhtml"&gt;concentration of
PD1 in synapse&lt;/body&gt;
        &lt;/notes&gt;
        &lt;annotation&gt;
          &lt;SimBiology xmlns="http://www.mathworks.com"&gt;
            &lt;Unit Numerator="molecule" Denominator="micrometer^2"/&gt;
          &lt;/SimBiology&gt;
        &lt;/annotation&gt;
      &lt;/species&gt;
      <species id="mw70a631d4_9f40_4bbb_bb75_a900f1130935" name="PDL1"
compartment="mwaad74090_4b37_47eb_b845_2f28d96c0e82"
initialConcentration="0" substanceUnits="MWBUILTINUNIT_molecule"
hasOnlySubstanceUnits="false" boundaryCondition="false"
constant="false">
        &lt;notes&gt;
          &lt;body xmlns="http://www.w3.org/1999/xhtml"&gt;concentration of
PDL1 in synapse&lt;/body&gt;
        &lt;/notes&gt;
        &lt;annotation&gt;
          &lt;SimBiology xmlns="http://www.mathworks.com"&gt;
            &lt;Unit Numerator="molecule" Denominator="micrometer^2"/&gt;
          &lt;/SimBiology&gt;
        &lt;/annotation&gt;
      &lt;/species&gt;
```

```
      <species id="mw5b51513e_0500_4cf8_a613_5a8041e539fd" name="PDL2"
compartment="mwaad74090_4b37_47eb_b845_2f28d96c0e82"
initialConcentration="0" substanceUnits="MWBUILTINUNIT_molecule"
hasOnlySubstanceUnits="false" boundaryCondition="false"
constant="false">
        &lt;notes&gt;
          &lt;body xmlns="http://www.w3.org/1999/xhtml"&gt;concentration of
PDL2 in synapse&lt;/body&gt;
        &lt;/notes&gt;
        &lt;annotation&gt;
          &lt;SimBiology xmlns="http://www.mathworks.com"&gt;
            &lt;Unit Numerator="molecule" Denominator="micrometer^2"/&gt;
          &lt;/SimBiology&gt;
        &lt;/annotation&gt;
      &lt;/species&gt;
      <species id="mwb8a9b9f0_4e26_4579_acf2_67e8a150f182"
name="PD1_aPD1" compartment="mwaad74090_4b37_47eb_b845_2f28d96c0e82"
initialConcentration="0" substanceUnits="MWBUILTINUNIT_molecule"
hasOnlySubstanceUnits="false" boundaryCondition="false"
constant="false">
        &lt;notes&gt;
          &lt;body xmlns="http://www.w3.org/1999/xhtml"&gt;concentration of
PD1-nivolumab complex&lt;/body&gt;
        &lt;/notes&gt;
        &lt;annotation&gt;
          &lt;SimBiology xmlns="http://www.mathworks.com"&gt;
            &lt;Unit Numerator="molecule" Denominator="micrometer^2"/&gt;
          &lt;/SimBiology&gt;
        &lt;/annotation&gt;
      &lt;/species&gt;
      <species id="mw89f4badf_d6b8_45ff_9012_7780c0a611d2"
name="PD1_aPD1_PD1"
compartment="mwaad74090_4b37_47eb_b845_2f28d96c0e82"
initialConcentration="0" substanceUnits="MWBUILTINUNIT_molecule"
hasOnlySubstanceUnits="false" boundaryCondition="false"
constant="false">
        &lt;notes&gt;
          &lt;body xmlns="http://www.w3.org/1999/xhtml"&gt;concentration of
PD1-nivolumab-PD1 complex&lt;/body&gt;
        &lt;/notes&gt;
        &lt;annotation&gt;
          &lt;SimBiology xmlns="http://www.mathworks.com"&gt;
            &lt;Unit Numerator="molecule" Denominator="micrometer^2"/&gt;
          &lt;/SimBiology&gt;
        &lt;/annotation&gt;
      &lt;/species&gt;
      <species id="mwf4dc2ee0_9bd9_4f51_ad22_d377e3e1668e"
name="PDL1_aPDL1" compartment="mwaad74090_4b37_47eb_b845_2f28d96c0e82"
initialConcentration="0" substanceUnits="MWBUILTINUNIT_molecule"
hasOnlySubstanceUnits="false" boundaryCondition="false"
constant="false">
        &lt;notes&gt;
```

```
          <body xmlns="http://www.w3.org/1999/xhtml">concentration of PDL1-durvalumab complex</body>
        </notes>
        <annotation>
          <SimBiology xmlns="http://www.mathworks.com">
            <Unit Numerator="molecule" Denominator="micrometer^2"/>
          </SimBiology>
        </annotation>
      </species>
      <species id="mw0c6e5a4d_1f16_46fd_b5b4_bb71908a3fa3" name="PDL1_aPDL1_PDL1" compartment="mwaad74090_4b37_47eb_b845_2f28d96c0e82" initialConcentration="0" substanceUnits="MWBUILTINUNIT_molecule" hasOnlySubstanceUnits="false" boundaryCondition="false" constant="false">
        <notes>
          <body xmlns="http://www.w3.org/1999/xhtml">concentration of PDL1-durvalumab-PDL1 complex</body>
        </notes>
        <annotation>
          <SimBiology xmlns="http://www.mathworks.com">
            <Unit Numerator="molecule" Denominator="micrometer^2"/>
          </SimBiology>
        </annotation>
      </species>
      <species id="mwf1568170_3b99_448b_94b1_035796c05f2f" name="CEA_cibis_CEA_CD3" compartment="mw0305c0ed_ac99_469b_8d32_3127d022edf9" initialConcentration="0" substanceUnits="MWBUILTINUNIT_molecule" hasOnlySubstanceUnits="false" boundaryCondition="false" constant="false">
        <notes>
          <body xmlns="http://www.w3.org/1999/xhtml">concentration of CEA-cibis-CEA_CD3 complex</body>
        </notes>
        <annotation>
          <SimBiology xmlns="http://www.mathworks.com">
            <Unit Numerator="molecule" Denominator="micrometer^2"/>
          </SimBiology>
        </annotation>
      </species>
      <species id="mw4f3f7cec_48cd_46bc_827c_b7fe282b5a76" name="CEA_cibis_CEA" compartment="mw0305c0ed_ac99_469b_8d32_3127d022edf9" initialConcentration="0" substanceUnits="MWBUILTINUNIT_molecule" hasOnlySubstanceUnits="false" boundaryCondition="false" constant="false">
        <notes>
          <body xmlns="http://www.w3.org/1999/xhtml">concentration of CEA-cibis-CEA complex</body>
        </notes>
        <annotation>
```

```
          <SimBiology xmlns="http://www.mathworks.com">
            <Unit Numerator="molecule" Denominator="micrometer^2"/>
          </SimBiology>
        </annotation>
      </species>
      <species id="mw0904c3c8_c9cd_43fd_82b9_e8a5772b6404"
name="CEA_cibis_CD3"
compartment="mw0305c0ed_ac99_469b_8d32_3127d022edf9"
initialConcentration="0" substanceUnits="MWBUILTINUNIT_molecule"
hasOnlySubstanceUnits="false" boundaryCondition="false"
constant="false">
        <notes>
          <body xmlns="http://www.w3.org/1999/xhtml">concentration of
CEA-cibis-CD3 complex</body>
        </notes>
        <annotation>
          <SimBiology xmlns="http://www.mathworks.com">
            <Unit Numerator="molecule" Denominator="micrometer^2"/>
          </SimBiology>
        </annotation>
      </species>
      <species id="mwd684b838_9206_4f20_b576_f4313889c57f"
name="CD3_cibis" compartment="mw0305c0ed_ac99_469b_8d32_3127d022edf9"
initialConcentration="0" substanceUnits="MWBUILTINUNIT_molecule"
hasOnlySubstanceUnits="false" boundaryCondition="false"
constant="false">
        <notes>
          <body xmlns="http://www.w3.org/1999/xhtml">concentration of
CD3-cibis complex</body>
        </notes>
        <annotation>
          <SimBiology xmlns="http://www.mathworks.com">
            <Unit Numerator="molecule" Denominator="micrometer^2"/>
          </SimBiology>
        </annotation>
      </species>
      <species id="mw748d24b3_d226_4e4d_9970_2410b77ccfa1"
name="CEA_cibis" compartment="mw0305c0ed_ac99_469b_8d32_3127d022edf9"
initialConcentration="0" substanceUnits="MWBUILTINUNIT_molecule"
hasOnlySubstanceUnits="false" boundaryCondition="false"
constant="false">
        <notes>
          <body xmlns="http://www.w3.org/1999/xhtml">concentration of
CEA-cibis complex</body>
        </notes>
        <annotation>
          <SimBiology xmlns="http://www.mathworks.com">
            <Unit Numerator="molecule" Denominator="micrometer^2"/>
          </SimBiology>
        </annotation>
      </species>
```

```
      <species id="mwa5aae57a_8cb6_4abc_8865_078cae23d61b" name="CEA"
compartment="mw0305c0ed_ac99_469b_8d32_3127d022edf9"
initialConcentration="0" substanceUnits="MWBUILTINUNIT_molecule"
hasOnlySubstanceUnits="false" boundaryCondition="false"
constant="false">
        <notes>
          <body xmlns="http://www.w3.org/1999/xhtml">concentration of
CEA in synapse</body>
        </notes>
        <annotation>
          <SimBiology xmlns="http://www.mathworks.com">
            <Unit Numerator="molecule" Denominator="micrometer^2"/>
          </SimBiology>
        </annotation>
      </species>
      <species id="mw5f168fc1_14e9_4b01_916e_4962d6278d2e" name="CD3"
compartment="mw0305c0ed_ac99_469b_8d32_3127d022edf9"
initialConcentration="0" substanceUnits="MWBUILTINUNIT_molecule"
hasOnlySubstanceUnits="false" boundaryCondition="false"
constant="false">
        <notes>
          <body xmlns="http://www.w3.org/1999/xhtml">concentration of
CD3 in synapse</body>
        </notes>
        <annotation>
          <SimBiology xmlns="http://www.mathworks.com">
            <Unit Numerator="molecule" Denominator="micrometer^2"/>
          </SimBiology>
        </annotation>
      </species>
      <species id="mw5c8acb40_e149_4324_a7a2_eadf8fda3445"
name="CEA_cibis_CEA_CD3"
compartment="mwfc416eeb_b169_4568_9e44_cab5996f5ee2"
initialConcentration="0" substanceUnits="MWBUILTINUNIT_molecule"
hasOnlySubstanceUnits="false" boundaryCondition="false"
constant="false">
        <notes>
          <body xmlns="http://www.w3.org/1999/xhtml">concentration of
CEA-cibis-CEA_CD3 complex</body>
        </notes>
        <annotation>
          <SimBiology xmlns="http://www.mathworks.com">
            <Unit Numerator="molecule" Denominator="micrometer^2"/>
          </SimBiology>
        </annotation>
      </species>
      <species id="mw31898027_19ab_4432_8d21_20da25633a64"
name="CEA_cibis_CEA"
compartment="mwfc416eeb_b169_4568_9e44_cab5996f5ee2"
initialConcentration="0" substanceUnits="MWBUILTINUNIT_molecule"
hasOnlySubstanceUnits="false" boundaryCondition="false"
constant="false">
```

```
        <notes>
          <body xmlns="http://www.w3.org/1999/xhtml">concentration of
CEA-cibis-CEA complex</body>
        </notes>
        <annotation>
          <SimBiology xmlns="http://www.mathworks.com">
            <Unit Numerator="molecule" Denominator="micrometer^2"/>
          </SimBiology>
        </annotation>
      </species>
      <species id="mw7b471287_592e_46b7_8683_9006b4bed437"
name="CEA_cibis_CD3"
compartment="mwfc416eeb_b169_4568_9e44_cab5996f5ee2"
initialConcentration="0" substanceUnits="MWBUILTINUNIT_molecule"
hasOnlySubstanceUnits="false" boundaryCondition="false"
constant="false">
        <notes>
          <body xmlns="http://www.w3.org/1999/xhtml">concentration of
CEA-cibis-CD3 complex</body>
        </notes>
        <annotation>
          <SimBiology xmlns="http://www.mathworks.com">
            <Unit Numerator="molecule" Denominator="micrometer^2"/>
          </SimBiology>
        </annotation>
      </species>
      <species id="mw41e864d0_1566_4d72_ba8a_d6991da393ff"
name="CD3_cibis" compartment="mwfc416eeb_b169_4568_9e44_cab5996f5ee2"
initialConcentration="0" substanceUnits="MWBUILTINUNIT_molecule"
hasOnlySubstanceUnits="false" boundaryCondition="false"
constant="false">
        <notes>
          <body xmlns="http://www.w3.org/1999/xhtml">concentration of
CD3-cibis complex</body>
        </notes>
        <annotation>
          <SimBiology xmlns="http://www.mathworks.com">
            <Unit Numerator="molecule" Denominator="micrometer^2"/>
          </SimBiology>
        </annotation>
      </species>
      <species id="mwdc4ebba0_e2e2_420e_ab38_3dd8a8a9a4b0"
name="CEA_cibis" compartment="mwfc416eeb_b169_4568_9e44_cab5996f5ee2"
initialConcentration="0" substanceUnits="MWBUILTINUNIT_molecule"
hasOnlySubstanceUnits="false" boundaryCondition="false"
constant="false">
        <notes>
          <body xmlns="http://www.w3.org/1999/xhtml">concentration of
CEA-cibis complex</body>
        </notes>
        <annotation>
          <SimBiology xmlns="http://www.mathworks.com">
```

BMJ Publishing Group Limited (BMJ) disclaims all liability and responsibility arising from any reliance placed on this supplemental material which has been supplied by the author(s)


```
            <Unit Numerator="molecule" Denominator="micrometer^2"/>
          </SimBiology>
        </annotation>
      </species>
      <species id="mw8cd989f8_196a_44a9_a449_5f7d44e2c0cf" name="CEA"
compartment="mwfc416eeb_b169_4568_9e44_cab5996f5ee2"
initialConcentration="0" substanceUnits="MWBUILTINUNIT_molecule"
hasOnlySubstanceUnits="false" boundaryCondition="false"
constant="false">
        <notes>
          <body xmlns="http://www.w3.org/1999/xhtml">concentration of
CEA in synapse</body>
        </notes>
        <annotation>
          <SimBiology xmlns="http://www.mathworks.com">
            <Unit Numerator="molecule" Denominator="micrometer^2"/>
          </SimBiology>
        </annotation>
      </species>
      <species id="mw16bdbdfb_62b5_45b6_987d_fae9a564f93e" name="CD3"
compartment="mwfc416eeb_b169_4568_9e44_cab5996f5ee2"
initialConcentration="0" substanceUnits="MWBUILTINUNIT_molecule"
hasOnlySubstanceUnits="false" boundaryCondition="false"
constant="false">
        <notes>
          <body xmlns="http://www.w3.org/1999/xhtml">concentration of
CD3 in synapse</body>
        </notes>
        <annotation>
          <SimBiology xmlns="http://www.mathworks.com">
            <Unit Numerator="molecule" Denominator="micrometer^2"/>
          </SimBiology>
        </annotation>
      </species>
    </listOfSpecies>
    <listOfParameters>
      <parameter id="mw1ab76cfe_e766_4ab0_b0c5_5162010849d6"
name="k_cell_clear" value="0.1" units="MWDERIVEDUNIT_1__day"
constant="true">
        <notes>
          <body xmlns="http://www.w3.org/1999/xhtml">Rate of dead cell
clearance from tumour compartment Rate of clearence of dead cancer
cells</body>
        </notes>
      </parameter>
      <parameter id="mw5c6752bd_6029_486c_b439_45f7ca1b3111"
name="cell" value="1" units="MWUSERUNIT_cell" constant="true"/>
      <parameter id="mwbdd9014c_961d_4199_a03d_419699e06107"
name="day" value="1" units="MWBUILTINUNIT_day" constant="true"/>
      <parameter id="mwe3f6b99e_81aa_4e82_b8e1_a3ae820e4aa5"
name="vol_cell" value="2527.31128244685"
units="MWDERIVEDUNIT_micrometer___3__cell" constant="true">
```

```
        <notes>
          <body xmlns="http://www.w3.org/1999/xhtml">Average volume of
cancer cell Volume of a single cancer cell</body>
        </notes>
      </parameter>
      <parameter id="mw2eb1dcb4_cb03_4de1_ae0e_ba7d9c54f167"
name="vol_Tcell" value="176"
units="MWDERIVEDUNIT_micrometer___3__cell" constant="true">
        <notes>
          <body xmlns="http://www.w3.org/1999/xhtml">Average volume of
T cells Volume of a single T cell</body>
        </notes>
      </parameter>
      <parameter id="mwa01f7b00_e009_4ea1_a621_7e3a90ebf97a"
name="V_Tmin" value="0.001"
units="MWBUILTINPREFIX_micro_MWBUILTINUNIT_liter" constant="true">
        <notes>
          <body xmlns="http://www.w3.org/1999/xhtml">Cancer-Free
Tumour compartment volumeCancer-free Tumour compartment volume</body>
        </notes>
      </parameter>
      <parameter id="mw1e7cee5a_6fd5_4375_9ff9_96509d2f0af7"
name="C_total" value="0" units="MWUSERUNIT_cell" constant="false"/>
      <parameter id="mw0782fa64_c077_445f_9358_fbb0e8cc0a7e"
name="T_total" value="0" units="MWUSERUNIT_cell" constant="false"/>
      <parameter id="mw5e8348dc_3969_4032_bf93_6f838d1e9121"
name="T_total_LN" value="0" units="MWUSERUNIT_cell" constant="false"/>
      <parameter id="mwcc034ae1_f0c4_4edf_b337_d36183873773"
name="R_Tcell" value="1" units="MWDERIVEDUNIT_cell__day"
constant="false"/>
      <parameter id="mwebb3353d_0237_474f_8045_a75656c37891"
name="Tregs_" value="0" units="MWUSERUNIT_cell" constant="false"/>
      <parameter id="mw38ea07af_24e1_43bd_b07b_ec2a1371f4d4"
name="H_APC" value="0.5" units="dimensionless" constant="false"/>
      <parameter id="mw154bd5ac_230a_4d71_be6f_5ab6ed5abe16"
name="H_mAPC" value="1" units="dimensionless" constant="false"/>
      <parameter id="mwffa3495a_26ce_48b7_bee5_50af90fed75f"
name="H_PD1_C1" value="1" units="dimensionless" constant="false"/>
      <parameter id="mw5fab6ae8_fb41_4c3a_818e_ce71aaaac5d9"
name="H_PD1_APC" value="1" units="dimensionless" constant="false"/>
      <parameter id="mw487f1739_cea4_4ce1_a649_f1ee4097f5aa"
name="H_CEA_C1_T1" value="0" units="dimensionless" constant="false"/>
      <parameter id="mwe4443c20_248a_4264_a3dc_ddda03ad2c04"
name="H_CEA_C1_T0" value="0" units="dimensionless" constant="false"/>
      <parameter id="mw79e9ce52_7313_4287_ad50_9c9cf1274898"
name="k_C1_growth" value="0.005" units="MWDERIVEDUNIT_1__day"
constant="true">
        <notes>
          <body xmlns="http://www.w3.org/1999/xhtml">Cancer cell
growth rate Cancer cell growth rate</body>
        </notes>
      </parameter>
```

```
      <parameter id="mwedddfe92_4421_4f29_8a47_d1b8a6db2304"
name="C_max" value="418879020478639" units="MWUSERUNIT_cell"
constant="true">
        <notes>
          <body xmlns="http://www.w3.org/1999/xhtml">Cancer cell
capacity Cancer cell capacity</body>
        </notes>
      </parameter>
      <parameter id="mw92cb2786_b098_48c2_a8dd_18d26e65a0d6"
name="k_C1_death" value="1e-05" units="MWDERIVEDUNIT_1__day"
constant="true">
        <notes>
          <body xmlns="http://www.w3.org/1999/xhtml">Cancer cell death
rate from innate immune cells Cancer cell death rate</body>
        </notes>
      </parameter>
      <parameter id="mw47b25c11_9847_4587_8215_ed3f3ef66f20"
name="k_C1_therapy" value="0" units="MWDERIVEDUNIT_1__day"
constant="false">
        <notes>
          <body xmlns="http://www.w3.org/1999/xhtml">Rate of C1
killing by therapy</body>
        </notes>
      </parameter>
      <parameter id="mwfbc673b2_613b_40e9_80e2_a3d56e48ab56"
name="initial_tumour_diameter" value="0.1"
units="MWBUILTINPREFIX_centi_MWBUILTINUNIT_meter" constant="true"/>
      <parameter id="mw98c1f539_f992_42f9_b09c_d98aa315dd63"
name="n_T1_clones" value="80" units="dimensionless" constant="true">
        <notes>
          <body xmlns="http://www.w3.org/1999/xhtml">Number of T cell
clones (estimated)</body>
        </notes>
      </parameter>
      <parameter id="mw6bb44ac8_1770_41f1_ab1d_0d1a00772b9a"
name="Q_T1_in" value="218.858981455742"
units="MWDERIVEDUNIT_cell__day" constant="true">
        <notes>
          <body xmlns="http://www.w3.org/1999/xhtml">Rate of naive T
cell transport into the lLN calculated based on naive T cell entry
rate estimated based on 12-hr dwell time of T cells in the LN (Germian
2012) and assuming 600 LN in the body (Ferrer 1998), T cell density in
the blood (Autissier 2010) and T cell diversity (Robins 2009)</body>
        </notes>
      </parameter>
      <parameter id="mwe7749870_048b_4ac0_844b_ef279fe1561f"
name="Q_T1_out" value="1.13" units="MWDERIVEDUNIT_1__day"
constant="true">
        <notes>
          <body xmlns="http://www.w3.org/1999/xhtml">Rate of naive T
cell transport out of the LN (Germain 2012)</body>
        </notes>
```

```
      </parameter>
      <parameter id="mwbebade6f_8329_4441_8a17_da5f70d2cb42"
name="k_T1_act" value="20" units="MWDERIVEDUNIT_1__day"
constant="true">
        <notes>
          <body xmlns="http://www.w3.org/1999/xhtml">T1 activation
rate</body>
        </notes>
      </parameter>
      <parameter id="mw2c17a42d_4cb1_43ab_90b6_6d1dbadc56de"
name="k_T1_pro" value="1" units="MWDERIVEDUNIT_1__day"
constant="true">
        <notes>
          <body xmlns="http://www.w3.org/1999/xhtml">T1 proliferation
rate</body>
        </notes>
      </parameter>
      <parameter id="mw9bcacce9_c511_4740_973e_f50fb674fd80"
name="k_T1_death" value="0.01" units="MWDERIVEDUNIT_1__day"
constant="true">
        <notes>
          <body xmlns="http://www.w3.org/1999/xhtml">T1 death
rate</body>
        </notes>
      </parameter>
      <parameter id="mw73b6655b_bc81_420b_bef6_4a23b4c57207"
name="q_T1_P_in" value="0.201" units="MWDERIVEDUNIT_1__minute"
constant="true">
        <notes>
          <body xmlns="http://www.w3.org/1999/xhtml">rate of T1
tranport into the peripheral compartment</body>
        </notes>
      </parameter>
      <parameter id="mwdc728989_1d46_4fe0_bc40_67ec5556584c"
name="q_T1_P_out" value="1" units="MWDERIVEDUNIT_1__day"
constant="true">
        <notes>
          <body xmlns="http://www.w3.org/1999/xhtml">rate of T1
tranport out of the peripheral compartment</body>
        </notes>
      </parameter>
      <parameter id="mw2fc20c96_b91f_40c9_b552_5aae17078d51"
name="q_T1_T_in" value="3.35e-06"
units="MWDERIVEDUNIT_1__centimeter___3_minute" constant="true">
        <notes>
          <body xmlns="http://www.w3.org/1999/xhtml">rate of T1
tranport into the tumour compartment</body>
        </notes>
      </parameter>
      <parameter id="mwecabb6dc_07d2_452b_b559_7a5a9c36e194"
name="q_T1_LN_out" value="24" units="MWDERIVEDUNIT_1__day"
constant="true">
```

```
        <notes>
          <body xmlns="http://www.w3.org/1999/xhtml">rate of T1
tranport out of the LN compartment</body>
        </notes>
      </parameter>
      <parameter id="mw06785562_a614_40f5_b1cf_23c5c2da8aba"
name="k_C_BTcell" value="1" units="MWDERIVEDUNIT_1__day"
constant="false">
        <notes>
          <body xmlns="http://www.w3.org/1999/xhtml">Rate of cancer
cell death by T cells (assumed)</body>
        </notes>
      </parameter>
      <parameter id="mw5c913adf_4222_49ab_bf00_936c2cac430d"
name="k_Treg_TCE" value="2" units="MWDERIVEDUNIT_1__day"
constant="false">
        <notes>
          <body xmlns="http://www.w3.org/1999/xhtml">Rate of cancer
cell death by T cells (guess)</body>
        </notes>
      </parameter>
      <parameter id="mwcd6f2806_c6ac_486c_b680_913fb6fc077b"
name="k_C_BTreg" value="1" units="MWDERIVEDUNIT_1__day"
constant="false">
        <notes>
          <body xmlns="http://www.w3.org/1999/xhtml">Rate of cancer
cell death by Tregs (assumed)</body>
        </notes>
      </parameter>
      <parameter id="mw8f2a737b_6631_4e62_834c_0e2ad84cb12a"
name="k_IL2_deg" value="0.2" units="MWDERIVEDUNIT_1__minute"
constant="true">
        <notes>
          <body xmlns="http://www.w3.org/1999/xhtml">rate of IL2
degradation (Lotze 1985)</body>
        </notes>
      </parameter>
      <parameter id="mw4db95ce7_153f_4c52_b346_bb639b69966e"
name="k_IL2_cons" value="6e-06"
units="MWDERIVEDUNIT_nanomole__cell__hour" constant="true">
        <notes>
          <body xmlns="http://www.w3.org/1999/xhtml">rate of IL2
consumption by T cells (Lotze 1985)</body>
        </notes>
      </parameter>
      <parameter id="mwa77be588_4158_477f_b088_450a8a14647d"
name="k_IL2_sec" value="3e-05"
units="MWDERIVEDUNIT_nanomole__cell__hour" constant="true">
        <notes>
          <body xmlns="http://www.w3.org/1999/xhtml">rate of IL2
secretion from T cells (Han 2012, Thurley 2015, Liu 2001)</body>
        </notes>
```

BMJ Publishing Group Limited (BMJ) disclaims all liability and responsibility arising from any reliance placed on this supplemental material which has been supplied by the author(s)


```
      </parameter>
      <parameter id="mwc266a39c_cd93_4ee9_a52b_c032c6a118f6"
name="IL2_50" value="0.32"
units="MWBUILTINPREFIX_nano_MWBUILTINUNIT_molarity" constant="true">
        <notes>
          <body xmlns="http://www.w3.org/1999/xhtml">T cell activation
half-maximal IL2 concentration (Marchingo 2014)</body>
        </notes>
      </parameter>
      <parameter id="mw470a2f94_f39a_45d1_bd57_2f506e768b0b"
name="IL2_50_Treg" value="0.0032"
units="MWBUILTINPREFIX_nano_MWBUILTINUNIT_molarity" constant="true">
        <notes>
          <body xmlns="http://www.w3.org/1999/xhtml">Treg activation
half-maximal IL2 concentration (Wang Smith 1987)</body>
        </notes>
      </parameter>
      <parameter id="mwb3dbb26b_dc9b_4dae_b56b_52700f65e4e5" name="N0"
value="5" units="dimensionless" constant="true">
        <notes>
          <body xmlns="http://www.w3.org/1999/xhtml">numer of
activated T cell generation with no IL2 (Marchingo 2014)</body>
        </notes>
      </parameter>
      <parameter id="mwcc68ed97_1448_4aa0_bf3e_a3ce54ccaca8"
name="N_IL2" value="11" units="dimensionless" constant="true">
        <notes>
          <body xmlns="http://www.w3.org/1999/xhtml">maximum number of
activated T cell generations due to IL2 (Marchingo 2014)</body>
        </notes>
      </parameter>
      <parameter id="mw7dd2d096_125c_43c8_8c6d_462231267034"
name="k_T1" value="0.1" units="MWDERIVEDUNIT_1__day" constant="true">
        <notes>
          <body xmlns="http://www.w3.org/1999/xhtml">Rate of T cell
exhaustion by cancer cells (estimated)</body>
        </notes>
      </parameter>
      <parameter id="mw4b836c57_546e_445b_b43d_95ce959cc91a"
name="k_C_T1" value="3" units="MWDERIVEDUNIT_1__day" constant="true">
        <notes>
          <body xmlns="http://www.w3.org/1999/xhtml">Rate of cancer
cell death by T cells (assumed)</body>
        </notes>
      </parameter>
      <parameter id="mw8108013c_5caa_4887_9636_3b1da28004b8"
name="k_Treg" value="2" units="MWDERIVEDUNIT_1__day" constant="true">
        <notes>
          <body xmlns="http://www.w3.org/1999/xhtml">Rate of T cell
death by Tregs (estimated)</body>
        </notes>
      </parameter>
```

```
      <parameter id="mw10ec1765_75ce_4474_94ca_6439ea9ef6cd"
name="H_P1" value="1" units="dimensionless" constant="false"/>
      <parameter id="mwc101eabc_ad45_48bb_b7ba_0a56d51fed98" name="a0"
value="0.692556926727918" units="dimensionless" constant="true"/>
      <parameter id="mw4c1ac185_3067_4da9_8869_b8094ae780f1" name="a1"
value="-0.096114835729841" units="dimensionless" constant="true"/>
      <parameter id="mw1ba3fe10_7bb4_4be9_9efa_e0e29c841c5b" name="a2"
value="0.00863812431352" units="dimensionless" constant="true"/>
      <parameter id="mwabc5258e_86ee_4684_ad1b_022fdf9808b2" name="a3"
value="-0.000403990701606" units="dimensionless" constant="true"/>
      <parameter id="mwc8e58a9d_94fd_46ae_88ea_d64803f582e1" name="a4"
value="7.59161205e-06" units="dimensionless" constant="true"/>
      <parameter id="mw71812882_d83d_43aa_a957_2dcfc26f16cb" name="m"
value="0" units="dimensionless" constant="false"/>
      <parameter id="mwf0b385a0_3c19_4518_9d9f_e58cad44b2ea" name="k"
value="0" units="dimensionless" constant="false"/>
      <parameter id="mw36a1fdc2_d829_49d9_8d94_a489fcce2a08"
name="k_ratio_1" value="0" units="dimensionless" constant="true"/>
      <parameter id="mwc87bd752_46f1_49c2_b111_e7505fd74ca8"
name="F_T1_act" value="0" units="MWUSERUNIT_cell" constant="false"/>
      <parameter id="mw41e31e9a_6f80_41a3_a437_bfaa6e8baed9"
name="N_aT" value="1" units="dimensionless" constant="false"/>
      <parameter id="mwcdf3bb71_6afa_48e7_b780_2d7ee5f9b5db"
name="n_T0_clones" value="100" units="dimensionless" constant="true">
        <notes>
          <body xmlns="http://www.w3.org/1999/xhtml">Number of T cell
clones (estimated)</body>
        </notes>
      </parameter>
      <parameter id="mw43bc1fbc_527c_4caa_b5f0_dacb9c792a18"
name="Q_T0_in" value="356.645243594488"
units="MWDERIVEDUNIT_cell__day" constant="true">
        <notes>
          <body xmlns="http://www.w3.org/1999/xhtml">Rate of naive T
cell transport into the lLN calculated based on naive T cell entry
rate estimated based on 12-hr dwell time of T cells in the LN (Germian
2012) and assuming 600 LN in the body (Ferrer 1998), T cell density in
the blood (Autissier 2010) and T cell diversity (Robins 2009)</body>
        </notes>
      </parameter>
      <parameter id="mw6657d584_3305_4159_9028_0fd387db86bc"
name="Q_T0_out" value="1.13" units="MWDERIVEDUNIT_1__day"
constant="true">
        <notes>
          <body xmlns="http://www.w3.org/1999/xhtml">Rate of naive T
cell transport out of the LN (Germain 2012)</body>
        </notes>
      </parameter>
      <parameter id="mwddb6a8f0_b347_4faa_9536_294915a489ea"
name="k_T0_act" value="20" units="MWDERIVEDUNIT_1__day"
constant="true">
        <notes>
```

BMJ Publishing Group Limited (BMJ) disclaims all liability and responsibility arising from any reliance placed on this supplemental material which has been supplied by the author(s)


```
          <body xmlns="http://www.w3.org/1999/xhtml">T0 activation
rate</body>
        </notes>
      </parameter>
      <parameter id="mw1f6f8208_3f72_4671_8286_8e080906848d"
name="k_T0_pro" value="1" units="MWDERIVEDUNIT_1__day"
constant="true">
        <notes>
          <body xmlns="http://www.w3.org/1999/xhtml">T0 proliferation
rate</body>
        </notes>
      </parameter>
      <parameter id="mw33b5d408_7337_4983_b330_cfcb3f4357af"
name="k_T0_death" value="0.01" units="MWDERIVEDUNIT_1__day"
constant="true">
        <notes>
          <body xmlns="http://www.w3.org/1999/xhtml">T0 death
rate</body>
        </notes>
      </parameter>
      <parameter id="mwddf444aa_74ff_4ba5_81a2_b30b34dc661e"
name="q_T0_P_in" value="0.201" units="MWDERIVEDUNIT_1__minute"
constant="true">
        <notes>
          <body xmlns="http://www.w3.org/1999/xhtml">rate of T0
tranport into the peripheral compartment</body>
        </notes>
      </parameter>
      <parameter id="mw6b8e638c_f28a_4c66_9b9c_c58ded48ccc0"
name="q_T0_P_out" value="0.015" units="MWDERIVEDUNIT_1__day"
constant="true">
        <notes>
          <body xmlns="http://www.w3.org/1999/xhtml">rate of T0
tranport out of the peripheral compartment</body>
        </notes>
      </parameter>
      <parameter id="mw302fac6a_5a44_40e3_a0dc_31785767fc4f"
name="q_T0_T_in" value="3.35e-06"
units="MWDERIVEDUNIT_1__centimeter___3_minute" constant="true">
        <notes>
          <body xmlns="http://www.w3.org/1999/xhtml">rate of T0
tranport into the tumour compartment</body>
        </notes>
      </parameter>
      <parameter id="mwbc88286a_79e7_4c26_a3ec_720caf764025"
name="q_T0_LN_out" value="24" units="MWDERIVEDUNIT_1__day"
constant="true">
        <notes>
          <body xmlns="http://www.w3.org/1999/xhtml">rate of T0
tranport out of the LN compartment</body>
        </notes>
      </parameter>
```

```
      <parameter id="mw1e889781_12c4_4172_91b6_546e3a00bfb4"
name="H_P0" value="1" units="dimensionless" constant="false"/>
      <parameter id="mwbb1a101c_bd5e_4dd7_b529_54e77169a609"
name="k_ratio_0" value="0" units="dimensionless" constant="true"/>
      <parameter id="mwdbdc3427_4618_4a63_be76_98a56e309b17"
name="F_T0_act" value="0" units="MWUSERUNIT_cell" constant="false"/>
      <parameter id="mw53fa75fa_b009_489f_9a91_2299c40b06e7"
name="k_APC_mat" value="1.5" units="MWDERIVEDUNIT_1__day"
constant="true">
        <notes>
          <body xmlns="http://www.w3.org/1999/xhtml">Maximum rate of
APC maturation</body>
        </notes>
      </parameter>
      <parameter id="mwa25696c4_7c1f_493f_9fcf_dfe77a664fda"
name="k_APC_mig" value="4" units="MWDERIVEDUNIT_1__day"
constant="true">
        <notes>
          <body xmlns="http://www.w3.org/1999/xhtml">Rate of APC
migration</body>
        </notes>
      </parameter>
      <parameter id="mwd682106f_de26_4639_8b62_e17205f22c2a"
name="k_APC_death" value="0.01" units="MWDERIVEDUNIT_1__day"
constant="true">
        <notes>
          <body xmlns="http://www.w3.org/1999/xhtml">Rate of APC
death</body>
        </notes>
      </parameter>
      <parameter id="mwb5663a72_7385_40b3_b101_1a11d4351ffb"
name="k_mAPC_death" value="0.02" units="MWDERIVEDUNIT_1__day"
constant="true">
        <notes>
          <body xmlns="http://www.w3.org/1999/xhtml">Rate of mAPC
death</body>
        </notes>
      </parameter>
      <parameter id="mw02c23a69_3001_4dcc_a56a_92c6ed513d09"
name="APC0_T" value="400000" units="MWDERIVEDUNIT_cell__milliliter"
constant="true">
        <notes>
          <body xmlns="http://www.w3.org/1999/xhtml">APC density in
the tumour</body>
        </notes>
      </parameter>
      <parameter id="mwf5121bef_af1c_473c_a722_fa4c65155afb"
name="APC0_LN" value="1200000" units="MWDERIVEDUNIT_cell__milliliter"
constant="true">
        <notes>
          <body xmlns="http://www.w3.org/1999/xhtml">APC density in
the LN</body>
```

```
        </notes>
      </parameter>
      <parameter id="mw7d571cef_3602_4299_9ec8_f9d570e17881"
name="k_c" value="2" units="MWDERIVEDUNIT_1__day" constant="true">
        <notes>
          <body xmlns="http://www.w3.org/1999/xhtml">Cytokine rate
constant</body>
        </notes>
      </parameter>
      <parameter id="mw5ccd755d_506a_494a_8e09_94b0a330e418" name="c0"
value="1e-09" units="MWBUILTINUNIT_molarity" constant="true">
        <notes>
          <body xmlns="http://www.w3.org/1999/xhtml">Baseline cytokine
concentration</body>
        </notes>
      </parameter>
      <parameter id="mwf7577d46_55a5_459f_86f6_36b2f0c1829b"
name="c50" value="1e-09" units="MWBUILTINUNIT_molarity"
constant="true">
        <notes>
          <body xmlns="http://www.w3.org/1999/xhtml">Cytokine
concentration for half-maximal APC maturation</body>
        </notes>
      </parameter>
      <parameter id="mwcb716e87_b63f_40df_a44f_1002c7d61986"
name="DAMPs" value="1.34e-14" units="MWDERIVEDUNIT_mole__cell"
constant="true">
        <notes>
          <body xmlns="http://www.w3.org/1999/xhtml">Concentration of
cytokines released per dying cancer cell Concentration of Cytokines
Released by Cancer Cell Death</body>
        </notes>
      </parameter>
      <parameter id="mw6d000fc0_ba86_41a7_beee_8cafe05139ce"
name="n_sites_APC" value="10" units="dimensionless" constant="true">
        <notes>
          <body xmlns="http://www.w3.org/1999/xhtml">Maxium number of
T Cells an APC can interact with Maximum Number of T Cells an APC can
Interact with</body>
        </notes>
      </parameter>
      <parameter id="mw3852f618_204c_448a_b245_9df43b665635"
name="kin" value="14.4" units="MWDERIVEDUNIT_1__day" constant="true">
        <notes>
          <body xmlns="http://www.w3.org/1999/xhtml">Rate of MHC
internalization Rate of MHC Internalization</body>
        </notes>
      </parameter>
      <parameter id="mw188d8839_a857_4645_a216_9e090aa6baad"
name="kout" value="28.8" units="MWDERIVEDUNIT_1__day" constant="true">
        <notes>
```

BMJ Publishing Group Limited (BMJ) disclaims all liability and responsibility arising from any reliance placed on this supplemental material which has been supplied by the author(s)


```
          <body xmlns="http://www.w3.org/1999/xhtml">Rate of MHC
externalization Rate of MHC Externalization</body>
        </notes>
      </parameter>
      <parameter id="mw6b11c414_a69b_4efd_8a07_d1173fa59a87"
name="k_P0_up" value="14.4" units="MWDERIVEDUNIT_1__day__cell"
constant="true">
        <notes>
          <body xmlns="http://www.w3.org/1999/xhtml">Rate of antigen
uptake by APCs Rate of Antigen Uptake</body>
        </notes>
      </parameter>
      <parameter id="mwc17972bb_efbb_4c99_ab02_61201c886f8d"
name="k_xP0_deg" value="2" units="MWDERIVEDUNIT_1__day"
constant="true">
        <notes>
          <body xmlns="http://www.w3.org/1999/xhtml">Rate of
extracellular antigen degradation Rate of Antigen Degradation in the
Extracellular Space</body>
        </notes>
      </parameter>
      <parameter id="mw3cf8aee1_8eb9_4ee4_93a7_61eb9f20252f"
name="k_P0_deg" value="17.28" units="MWDERIVEDUNIT_1__day"
constant="true">
        <notes>
          <body xmlns="http://www.w3.org/1999/xhtml">Rate of endosomal
antigen degradation Rate of Antigen Degradation in Endosomes</body>
        </notes>
      </parameter>
      <parameter id="mw08e80428_100e_4181_8dff_866054ccd158"
name="k_p0_deg" value="144" units="MWDERIVEDUNIT_1__day"
constant="true">
        <notes>
          <body xmlns="http://www.w3.org/1999/xhtml">Rate of endosomal
epitope degradation Rate of Epitope Degradation</body>
        </notes>
      </parameter>
      <parameter id="mw33d69d64_f4f9_4384_9195_c2233607df55"
name="k_P0_on" value="144000" units="MWDERIVEDUNIT_1__day__molarity"
constant="true">
        <notes>
          <body xmlns="http://www.w3.org/1999/xhtml">Rate of antigen-
MHC binding Antigen Association Constant</body>
        </notes>
      </parameter>
      <parameter id="mw8cb23925_0616_4ba0_b05f_b7c310055464"
name="k_P0_d1" value="1e-07" units="MWBUILTINUNIT_molarity"
constant="true">
        <notes>
          <body xmlns="http://www.w3.org/1999/xhtml">Antigen-MHC
kd</body>
        </notes>
```

```
      </parameter>
      <parameter id="mw45d28c78_e295_422a_93e8_63fd5c065117"
name="p0_50" value="2.64550264550265e-05"
units="MWDERIVEDUNIT_molecule__micrometer___2" constant="true">
        <notes>
          <body xmlns="http://www.w3.org/1999/xhtml">MHC-Epitope-TCR
Concentration for Half-Maximal T Cell Activation</body>
        </notes>
      </parameter>
      <parameter id="mw62203837_dd40_4baa_8b94_89d3fc6cc634"
name="P0_C1" value="1e-08" units="MWDERIVEDUNIT_molarity__cell"
constant="true">
        <notes>
          <body xmlns="http://www.w3.org/1999/xhtml">Concentration of
P0 in C1</body>
        </notes>
      </parameter>
      <parameter id="mw91c9d65f_4e18_48ad_a863_b4572ca1bc2e"
name="A_syn" value="37.8" units="MWDERIVEDUNIT_micrometer___2"
constant="true">
        <notes>
          <body xmlns="http://www.w3.org/1999/xhtml">Surface area of
the synapse Synapse Surface Area</body>
        </notes>
      </parameter>
      <parameter id="mw1968e71e_f5e7_4518_bf96_58e98a32246f"
name="A_Tcell" value="151.310411930437"
units="MWDERIVEDUNIT_micrometer___2" constant="true">
        <notes>
          <body xmlns="http://www.w3.org/1999/xhtml">Surface area of
the T cell T Cell Surface Area</body>
        </notes>
      </parameter>
      <parameter id="mwf55bfe7a_0ad3_448f_b02b_5d85aca0aae5"
name="A_cell" value="897.270277791781"
units="MWDERIVEDUNIT_micrometer___2" constant="true">
        <notes>
          <body xmlns="http://www.w3.org/1999/xhtml">Surface area of
the Cancer cell Cancer Cell Surface Area</body>
        </notes>
      </parameter>
      <parameter id="mw3a89a2a1_413a_48ba_b39d_abce742943a1"
name="A_APC" value="900" units="MWDERIVEDUNIT_micrometer___2"
constant="true">
        <notes>
          <body xmlns="http://www.w3.org/1999/xhtml">Surface area of
the APC APC Surface Area</body>
        </notes>
      </parameter>
      <parameter id="mw1bdcde86_7239_49ff_a50c_244b38426b19"
name="k_M1p0_TCR_on" value="1"
```

```
units="MWDERIVEDUNIT_1__second_molecule__micrometer___2"
constant="true">
        <notes>
          <body xmlns="http://www.w3.org/1999/xhtml">Rate of TCR
binding to MHC-peptide complex Rate of TCR Binding</body>
        </notes>
      </parameter>
      <parameter id="mwe1d5301d_65a6_4805_a0c4_b9488461ccc8"
name="k_M1p0_TCR_off" value="1" units="MWDERIVEDUNIT_1__second"
constant="true">
        <notes>
          <body xmlns="http://www.w3.org/1999/xhtml">Rate of TCR
unbinding from MHC-peptide complex Rate of TCR Unbinding</body>
        </notes>
      </parameter>
      <parameter id="mw1390963b_ac2e_43bf_9068_18e15a3f8933"
name="TCR_p0_tot" value="103.813080670361"
units="MWDERIVEDUNIT_molecule__micrometer___2" constant="true">
        <notes>
          <body xmlns="http://www.w3.org/1999/xhtml">Total number of
TCR molecules per naive T cell Number of TCR Molecules on Naive T
Cells per Unit Area</body>
        </notes>
      </parameter>
      <parameter id="mwb2d4fc83_566f_4b7d_9037_c52ec0a459af"
name="pTCR_p0_MHC_tot" value="0"
units="MWDERIVEDUNIT_molecule__micrometer___2" constant="false">
        <notes>
          <body xmlns="http://www.w3.org/1999/xhtml">Total number of
MHC-p0-TCR complexes of all different activation levels</body>
        </notes>
      </parameter>
      <parameter id="mw96f9fe99_e334_402d_b28a_00dbdf7c575f"
name="k_P1_up" value="14.4" units="MWDERIVEDUNIT_1__day__cell"
constant="true">
        <notes>
          <body xmlns="http://www.w3.org/1999/xhtml">Rate of antigen
uptake by APCs Rate of Antigen Uptake</body>
        </notes>
      </parameter>
      <parameter id="mw853e9708_350e_4ea7_8907_ea58cfa95380"
name="k_xP1_deg" value="2" units="MWDERIVEDUNIT_1__day"
constant="true">
        <notes>
          <body xmlns="http://www.w3.org/1999/xhtml">Rate of
extracellular antigen degradation Rate of Antigen Degradation in the
Extracellular Space</body>
        </notes>
      </parameter>
      <parameter id="mwf862cc85_c33f_4654_a561_ae76fa81bb97"
name="k_P1_deg" value="17.28" units="MWDERIVEDUNIT_1__day"
constant="true">
```

```
        <notes>
          <body xmlns="http://www.w3.org/1999/xhtml">Rate of endosomal
antigen degradation Rate of Antigen Degradation in Endosomes</body>
        </notes>
      </parameter>
      <parameter id="mwfc10a527_910a_4ef1_8947_e37acf0cf148"
name="k_p1_deg" value="144" units="MWDERIVEDUNIT_1__day"
constant="true">
        <notes>
          <body xmlns="http://www.w3.org/1999/xhtml">Rate of endosomal
epitope degradation Rate of Epitope Degradation</body>
        </notes>
      </parameter>
      <parameter id="mwddabbddc_09d9_4688_97e3_0ec72d15abaf"
name="k_P1_on" value="144000" units="MWDERIVEDUNIT_1__day__molarity"
constant="true">
        <notes>
          <body xmlns="http://www.w3.org/1999/xhtml">Rate of antigen-
MHC binding Antigen Association Constant</body>
        </notes>
      </parameter>
      <parameter id="mw0c9670f6_ecd6_4631_90cc_30463073a545"
name="k_P1_d1" value="4e-08" units="MWBUILTINUNIT_molarity"
constant="true">
        <notes>
          <body xmlns="http://www.w3.org/1999/xhtml">Antigen-MHC
kd</body>
        </notes>
      </parameter>
      <parameter id="mw382b7a70_e63f_4a14_b5ae_03012ce36574"
name="p1_50" value="2.64550264550265e-05"
units="MWDERIVEDUNIT_molecule__micrometer___2" constant="true">
        <notes>
          <body xmlns="http://www.w3.org/1999/xhtml">MHC-Epitope-TCR
Concentration for Half-Maximal T Cell Activation</body>
        </notes>
      </parameter>
      <parameter id="mw09a78ea1_f99a_49fb_8f57_f79df3425689"
name="P1_C1" value="1e-08" units="MWDERIVEDUNIT_molarity__cell"
constant="true">
        <notes>
          <body xmlns="http://www.w3.org/1999/xhtml">Concentration of
P1 in C1</body>
        </notes>
      </parameter>
      <parameter id="mwe857ab2a_b73e_41db_9689_4fce13b4c5f9"
name="k_M1p1_TCR_on" value="1"
units="MWDERIVEDUNIT_1__second_molecule__micrometer___2"
constant="true">
        <notes>
          <body xmlns="http://www.w3.org/1999/xhtml">Rate of TCR
binding to MHC-peptide complex Rate of TCR Binding</body>
```

```
        </notes>
      </parameter>
      <parameter id="mw3e998fd3_0e21_483b_a0dc_f9717401fc48"
name="k_M1p1_TCR_off" value="1" units="MWDERIVEDUNIT_1__second"
constant="true">
        <notes>
          <body xmlns="http://www.w3.org/1999/xhtml">Rate of TCR
unbinding from MHC-peptide complex Rate of TCR Unbinding</body>
        </notes>
      </parameter>
      <parameter id="mw0231f6de_2302_4910_aa44_dbb729a3e732"
name="k_M1p1_TCR_p" value="1" units="MWDERIVEDUNIT_1__second"
constant="true">
        <notes>
          <body xmlns="http://www.w3.org/1999/xhtml">Rate of MHC-
peptide-TCR complex modification Rate of TCR Modification</body>
        </notes>
      </parameter>
      <parameter id="mw0fc88c2e_4d0b_4fc9_ad59_3b84599698f7"
name="phi_M1p1_TCR" value="0.09" units="MWDERIVEDUNIT_1__second"
constant="true">
        <notes>
          <body xmlns="http://www.w3.org/1999/xhtml">Rate of MHC-
peptide-TCR complex with maximal modification that leads to non-
signalingRate of TCR Modification Leading to Non-Signaling</body>
        </notes>
      </parameter>
      <parameter id="mwf5abafbe_c4ea_43d1_917a_1e0f82adcb97"
name="N_M1p1_TCR" value="10" units="dimensionless" constant="true">
        <notes>
          <body xmlns="http://www.w3.org/1999/xhtml">Number of
modifcation steps for MHC-peptide-TCR complex Number of Intermediate
Steps</body>
        </notes>
      </parameter>
      <parameter id="mw3edb1bd6_42e5_4c85_bd91_3427b4480151"
name="TCR_p1_tot" value="103.813080670361"
units="MWDERIVEDUNIT_molecule__micrometer___2" constant="true">
        <notes>
          <body xmlns="http://www.w3.org/1999/xhtml">Total number of
TCR molecules per naive T cell Number of TCR Molecules on Naive T
Cells per Unit Area</body>
        </notes>
      </parameter>
      <parameter id="mw0bcc5b8c_3735_4aa8_be25_eeed6fb095d1"
name="pTCR_p1_MHC_tot" value="0"
units="MWDERIVEDUNIT_molecule__micrometer___2" constant="false">
        <notes>
          <body xmlns="http://www.w3.org/1999/xhtml">Total number of
MHC-p1-TCR complexes of all different activation levels</body>
        </notes>
      </parameter>
```

```xml
      <parameter id="mwe8d5d721_005f_49f7_ada6_e82cd8ca00c6"
name="kon_PD1_PDL1" value="0.0583333333333333"
units="MWDERIVEDUNIT_1__micromolarity_nanometer_second"
constant="true">
        <notes>
          <body xmlns="http://www.w3.org/1999/xhtml">kon of PD1-PDL1
binding PD1-PDL1 Binding Rate</body>
        </notes>
      </parameter>
      <parameter id="mw75ebeaf4_1dd4_4b61_bdf7_0611af968ca8"
name="aPD1" value="0" units="MWBUILTINUNIT_molarity" constant="false">
        <notes>
          <body xmlns="http://www.w3.org/1999/xhtml">Concentration of
PD1 antibody</body>
        </notes>
      </parameter>
      <parameter id="mw140e5cc4_b897_4719_aa3d_0b30f25bc4dd"
name="aPDL1" value="0" units="MWBUILTINUNIT_molarity"
constant="false">
        <notes>
          <body xmlns="http://www.w3.org/1999/xhtml">Concentration of
PDL1 antibody</body>
        </notes>
      </parameter>
      <parameter id="mw01026970_e68e_4663_a8f4_3c89fbb89a28"
name="q_P_atezolizumab" value="6.97743585e-08"
units="MWDERIVEDUNIT_milliliter__second" constant="true"/>
      <parameter id="mwb04099fe_184d_408f_b64a_5211b48295fa"
name="q_T_atezolizumab" value="8.52e-06"
units="MWDERIVEDUNIT_1__second" constant="true"/>
      <parameter id="mwbfae92a6_4484_4c8d_954b_821e213acb8f"
name="q_LN_atezolizumab" value="6.97743585e-08"
units="MWDERIVEDUNIT_milliliter__second" constant="true"/>
      <parameter id="mw4478efbf_cc05_4bfb_a0f1_51d7236455dd"
name="q_LD_atezolizumab" value="0.0015"
units="MWDERIVEDUNIT_1__minute" constant="true"/>
      <parameter id="mwb815e7ef_6822_4c31_ad87_3283f2e76ec4"
name="k_cl_atezolizumab" value="0.003" units="MWDERIVEDUNIT_1__hour"
constant="true"/>
      <parameter id="mwa5c3d9de_dbc4_4f10_a373_24389f61938a"
name="gamma_C_atezolizumab" value="0.3643" units="dimensionless"
constant="true"/>
      <parameter id="mw81e3f6ab_7c90_45d2_828d_5d9e8d54f20c"
name="gamma_P_atezolizumab" value="0.0405" units="dimensionless"
constant="true"/>
      <parameter id="mwe8121890_8596_4287_b3ee_50b05edf5540"
name="gamma_T_atezolizumab" value="0.522" units="dimensionless"
constant="true"/>
      <parameter id="mw3901f164_3d95_4e77_ad0b_2d4452a9d976"
name="gamma_LN_atezolizumab" value="0.2" units="dimensionless"
constant="true"/>
```

```xml
      <parameter id="mw4cfa47a0_69d4_4eec_aa6c_e4470eeeb266" name="kon_PD1_PDL2" value="0.0766666666666667" units="MWDERIVEDUNIT_1__micromolarity_nanometer_second" constant="true">
        <notes>
          <body xmlns="http://www.w3.org/1999/xhtml">kon of PD1-PDL2 binding PD1-PDL2 Binding Rate</body>
        </notes>
      </parameter>
      <parameter id="mw8db9064f_6e42_49c7_8a0b_a47da71a3758" name="kon_PD1_aPD1" value="1300000" units="MWDERIVEDUNIT_1__molarity_second" constant="true">
        <notes>
          <body xmlns="http://www.w3.org/1999/xhtml">kon of PD1-antiPD1 binding PD1-nivolumab Binding Rate Constant</body>
        </notes>
      </parameter>
      <parameter id="mwe0f00ed8_dcf4_4859_9755_bb24cd7f35c9" name="kon_PDL1_aPDL1" value="430000" units="MWDERIVEDUNIT_1__molarity_second" constant="true">
        <notes>
          <body xmlns="http://www.w3.org/1999/xhtml">kon of PDL1-antiPDL1 binding PDL1-durvalumab Binding Rate Constant</body>
        </notes>
      </parameter>
      <parameter id="mw75aefaf3_1856_43cf_8511_2fa3e0451e0b" name="koff_PD1_PDL1" value="1.435" units="MWDERIVEDUNIT_1__second" constant="true">
        <notes>
          <body xmlns="http://www.w3.org/1999/xhtml">koff of PD1-PDL1 binding PD1-PDL1 Unbinding Rate</body>
        </notes>
      </parameter>
      <parameter id="mw93bf4558_6b97_4f12_bc08_c258a1086db9" name="koff_PD1_PDL2" value="0.529" units="MWDERIVEDUNIT_1__second" constant="true">
        <notes>
          <body xmlns="http://www.w3.org/1999/xhtml">koff of PD1-PDL2 binding PD1-PDL2 Unbinding Rate</body>
        </notes>
      </parameter>
      <parameter id="mw991ab93b_f5ef_47e4_8661_9588c62249e4" name="koff_PD1_aPD1" value="0.00338" units="MWDERIVEDUNIT_1__second" constant="true">
        <notes>
          <body xmlns="http://www.w3.org/1999/xhtml">koff of PD1-nivolumab binding PD1-aPD1 Unbinding Rate</body>
        </notes>
      </parameter>
      <parameter id="mwe82cc259_fdc8_4239_a6e1_1f0fea41ca6d" name="koff_PDL1_aPDL1" value="0.000172" units="MWDERIVEDUNIT_1__second" constant="true">
```

```
        <notes>
          <body xmlns="http://www.w3.org/1999/xhtml">koff of PDL1-
durvalumab binding PDL1-aPDL1 Unbinding Rate</body>
        </notes>
      </parameter>
      <parameter id="mw614c8d32_2578_4c5c_ba98_2300e829174f"
name="Chi_PD1" value="3.33333333333333"
units="MWDERIVEDUNIT_1__nanometer" constant="true">
        <notes>
          <body xmlns="http://www.w3.org/1999/xhtml">Antibody cross-
arm binding efficiency PD1 Antibody Cross-Arm Binding Strength
Corrected for 2D</body>
        </notes>
      </parameter>
      <parameter id="mw3e179651_289c_42d8_bfe9_a4412cee4d9c"
name="Chi_PDL1" value="33.3333333333333"
units="MWDERIVEDUNIT_1__nanometer" constant="true">
        <notes>
          <body xmlns="http://www.w3.org/1999/xhtml">Antibody cross-
arm binding efficiency PDL1 Antibody Cross-Arm Binding Strength
Corrected for 2D</body>
        </notes>
      </parameter>
      <parameter id="mwe2df21f5_c609_4db4_800f_b318a4cd9ebd"
name="PD1_50" value="6" units="MWDERIVEDUNIT_molecule__micrometer___2"
constant="true">
        <notes>
          <body xmlns="http://www.w3.org/1999/xhtml">PD1/PDL1
concentration for half-maximal T cell inactivation PD1-PDL1
Concentration for Half-Maximal PD1-Induced Reduction of T Cell
Killing</body>
        </notes>
      </parameter>
      <parameter id="mw8cda36fb_5234_46ed_bdb8_520a142b8826"
name="n_PD1" value="2" units="dimensionless" constant="true">
        <notes>
          <body xmlns="http://www.w3.org/1999/xhtml">Hill coefficient
for PD1/PDL1 half-maximal T cell inactivation Hill Coefficient for
PD1-Induced Reduction of T Cell Killing</body>
        </notes>
      </parameter>
      <parameter id="mw27d20453_a8b4_4947_bc96_a82d24a92d58"
name="T_PD1_total" value="60000" units="MWBUILTINUNIT_molecule"
constant="false">
        <notes>
          <body xmlns="http://www.w3.org/1999/xhtml">Concentration of
PD1 on T cells Total number of PD1 Molecules on T Cells</body>
        </notes>
      </parameter>
      <parameter id="mwdf582c9f_98b3_4fd2_a1b2_9c10ed295a43"
name="C_PDL1_total" value="400000" units="MWBUILTINUNIT_molecule"
constant="false">
```

```
        <notes>
          <body xmlns="http://www.w3.org/1999/xhtml">Number of PDL1
molecules per cancer cell Total number of PDL1 Molecules on Cancer
Cells</body>
        </notes>
      </parameter>
      <parameter id="mwad42c5a9_e21f_4100_add0_623b9a89169c"
name="APC_PDL1_total" value="1600000" units="MWBUILTINUNIT_molecule"
constant="false">
        <notes>
          <body xmlns="http://www.w3.org/1999/xhtml">Number of PDL1
molecules per APC Total number of PDL1 Molecules on Cancer
Cells</body>
        </notes>
      </parameter>
      <parameter id="mwcb443c9f_9d14_4279_aa40_7513a4dd8f48"
name="r_PDL2C" value="0.05" units="dimensionless" constant="false">
        <notes>
          <body xmlns="http://www.w3.org/1999/xhtml">Number of PDL1
molecules per APC estimated</body>
        </notes>
      </parameter>
      <parameter id="mwe3d9a595_a61c_479a_9ef5_e5a6aa984d65"
name="r_PDL2APC" value="0.05" units="dimensionless" constant="false">
        <notes>
          <body xmlns="http://www.w3.org/1999/xhtml">Number of PDL2
molecules per APC estimated</body>
        </notes>
      </parameter>
      <parameter id="mw4e644bb6_5cf2_4286_ad23_2992c5369649"
name="T_syn" value="75.6" units="MWDERIVEDUNIT_micrometer___2"
constant="true">
        <notes>
          <body xmlns="http://www.w3.org/1999/xhtml">Surface area of
the synapse (Janssen 2007)</body>
        </notes>
      </parameter>
      <parameter id="mw36366027_1d43_45e4_a407_edd118db612a"
name="q_P_cibis" value="2.755e-07"
units="MWDERIVEDUNIT_milliliter__second" constant="true"/>
      <parameter id="mw7ca21d14_d8be_4151_a1ca_04e2bfeabb57"
name="q_T_cibis" value="8.52e-06" units="MWDERIVEDUNIT_1__second"
constant="true"/>
      <parameter id="mwe6152cdd_350a_4c16_81ca_1e481e6c0a64"
name="q_LN_cibis" value="2.755e-07"
units="MWDERIVEDUNIT_milliliter__second" constant="true"/>
      <parameter id="mw874a6840_c8e2_4455_888c_3f46f6b9f513"
name="q_LD_cibis" value="0.0015" units="MWDERIVEDUNIT_1__minute"
constant="true"/>
      <parameter id="mw41dce75f_74ae_4cf2_8cf1_4733ddbc9d1a"
name="k_cl_cibis" value="0.025" units="MWDERIVEDUNIT_1__hour"
constant="true"/>
```

```
      <parameter id="mw8b890aa4_5a8d_494f_a9dc_94dd9102747d"
name="gamma_C_cibis" value="0.46" units="dimensionless"
constant="true"/>
      <parameter id="mw0a99753d_641d_4691_8979_ff41ab8c42c4"
name="gamma_P_cibis" value="0.067" units="dimensionless"
constant="true"/>
      <parameter id="mw9d3532da_0dcb_480c_9ec6_05144794ed9e"
name="gamma_T_cibis" value="0.522" units="dimensionless"
constant="true"/>
      <parameter id="mw035bd865_eaf6_4933_a20e_09f1ca241df0"
name="gamma_LN_cibis" value="0.2" units="dimensionless"
constant="true"/>
      <parameter id="mwfffe4beb_6c50_42ac_8f60_6131e3e8cc8b"
name="dT_syn" value="3"
units="MWBUILTINPREFIX_nano_MWBUILTINUNIT_meter" constant="true">
        <notes>
          <body xmlns="http://www.w3.org/1999/xhtml">Distance between
two cells in the synapse (Janssen 2007)</body>
        </notes>
      </parameter>
      <parameter id="mw6014386a_19d2_4a74_bb82_c32db3ebe899"
name="SA_Tcell" value="151.310411930437"
units="MWDERIVEDUNIT_micrometer___2" constant="true">
        <notes>
          <body xmlns="http://www.w3.org/1999/xhtml">Surface area of
the T cell calculated based on the average T cell diameter (Chapman
1981)</body>
        </notes>
      </parameter>
      <parameter id="mwd676f62b_ea64_4770_b805_03a42fe744f5"
name="SA_cell" value="1063.61760879936"
units="MWDERIVEDUNIT_micrometer___2" constant="true">
        <notes>
          <body xmlns="http://www.w3.org/1999/xhtml">Surface area of
the Cancer cell calculated based on the average Cancer cell diameter
(Hosokawa 2013; stage IV NSCLC)</body>
        </notes>
      </parameter>
      <parameter id="mwbb6b5fad_79fc_4c1e_8c28_716ef8102b7f"
name="SA_APC" value="900" units="MWDERIVEDUNIT_micrometer___2"
constant="true">
        <notes>
          <body xmlns="http://www.w3.org/1999/xhtml">Surface area of
the APC (Agrawal 1996)</body>
        </notes>
      </parameter>
      <parameter id="mw15df1616_97ed_4ca4_b30e_ebb077cf4325"
name="kon_CD3_cibis" value="10000"
units="MWDERIVEDUNIT_1__molarity_second" constant="true">
        <notes>
          <body xmlns="http://www.w3.org/1999/xhtml">kon of CD3-cibis
binding kon for CD3-cibis (Steffi Lehmann 2016)</body>
```

```
        </notes>
      </parameter>
      <parameter id="mw9cf96b22_1a33_4f87_afcd_68dba7e3c4a6"
name="kon_CEA_cibis" value="1000"
units="MWDERIVEDUNIT_1__molarity_second" constant="true">
        <notes>
          <body xmlns="http://www.w3.org/1999/xhtml">kon of CEA-cibis
binding kon for CD3-cibis (Steffi Lehmann 2016)</body>
        </notes>
      </parameter>
      <parameter id="mw65b50fa7_efcb_4017_9c8e_0221fab22f43"
name="kon_CD3_cibis2" value="3333.33333333333"
units="MWDERIVEDUNIT_1__molarity_nanometer_second" constant="true">
        <notes>
          <body xmlns="http://www.w3.org/1999/xhtml">kon of CD3-cibis
binding kon for CD3-cibis (Steffi Lehmann 2016)</body>
        </notes>
      </parameter>
      <parameter id="mw7d525f58_1dbe_4e8b_95e9_c1716c018ebd"
name="kon_CEA_cibis2" value="333.333333333333"
units="MWDERIVEDUNIT_1__molarity_nanometer_second" constant="true">
        <notes>
          <body xmlns="http://www.w3.org/1999/xhtml">kon of CEA-cibis
binding kon for CD3-cibis (Steffi Lehmann 2016)</body>
        </notes>
      </parameter>
      <parameter id="mwf8e07c6f_de10_40ed_8fed_c4b415ac88ff"
name="koff_CD3_cibis" value="0.00075" units="MWDERIVEDUNIT_1__second"
constant="true">
        <notes>
          <body xmlns="http://www.w3.org/1999/xhtml">koff of CD3-cibis
binding calculated based on the measured kd and kon (Steffi Lehmann
2016)</body>
        </notes>
      </parameter>
      <parameter id="mw254b6741_04ff_476a_86b0_e18f1f7448a6"
name="koff_CEA_cibis" value="0.00013" units="MWDERIVEDUNIT_1__second"
constant="true">
        <notes>
          <body xmlns="http://www.w3.org/1999/xhtml">koff of CEA-cibis
binding calculated based on the measured kd and kon (Steffi Lehmann
2016)</body>
        </notes>
      </parameter>
      <parameter id="mwc205da12_9624_4676_ba4c_e1d6d6b18abd"
name="Chi_CEA_cibis" value="0.333333330953227"
units="MWDERIVEDUNIT_1__nanometer" constant="true">
        <notes>
          <body xmlns="http://www.w3.org/1999/xhtml">Antibody cross-
arm binding efficiency  that also includes the conversion of kon from
3D to 2D(Estimated based on Wang 2014)</body>
        </notes>
```

```
      </parameter>
      <parameter id="mwaecb4a00_67ae_4818_8f41_195a5060534e"
name="CEA_CD3_50" value="8"
units="MWDERIVEDUNIT_molecule__micrometer___2" constant="true">
        <notes>
          <body xmlns="http://www.w3.org/1999/xhtml">CEA/CD3
concentration for half-maximal T cell inactivation (Jonathan
2016)</body>
        </notes>
      </parameter>
      <parameter id="mwdbca445b_0370_40eb_9cb0_41018b8b2740"
name="n_CEA_CD3" value="3" units="dimensionless" constant="true">
        <notes>
          <body xmlns="http://www.w3.org/1999/xhtml">Hill coefficient
for CEA/CD3 half-maximal T cell inactivation (estimated?)</body>
        </notes>
      </parameter>
      <parameter id="mwb002721a_80c2_4e13_ab14_bc346c326535"
name="T1_CD3_total" value="62000" units="MWBUILTINUNIT_molecule"
constant="false">
        <notes>
          <body xmlns="http://www.w3.org/1999/xhtml">concentration of
CD3 on T1 cells (guess)</body>
        </notes>
      </parameter>
      <parameter id="mw9da4d78b_cfec_4e11_b021_8e57ba26a901"
name="C1_CEA_total" value="20000" units="MWBUILTINUNIT_molecule"
constant="false">
        <notes>
          <body xmlns="http://www.w3.org/1999/xhtml">number of CEA
molecules per C1 cell (guess)</body>
        </notes>
      </parameter>
      <parameter id="mw8947be8c_bc84_4748_bf0e_d682b86f2ec4"
name="T0_CD3_total" value="62000" units="MWBUILTINUNIT_molecule"
constant="false">
        <notes>
          <body xmlns="http://www.w3.org/1999/xhtml">concentration of
CD3 on T0 cells (guess)</body>
        </notes>
      </parameter>
    </listOfParameters>
    <listOfInitialAssignments>
      <initialAssignment
symbol="mw36a1fdc2_d829_49d9_8d94_a489fcce2a08">
        <math xmlns="http://www.w3.org/1998/Math/MathML">
          <apply>
            <divide/>
            <ci> mw2c17a42d_4cb1_43ab_90b6_6d1dbadc56de </ci>
            <ci> mwbebade6f_8329_4441_8a17_da5f70d2cb42 </ci>
          </apply>
        </math>
```

```
      </initialAssignment>
      <initialAssignment
symbol="mwbb1a101c_bd5e_4dd7_b529_54e77169a609">
        <math xmlns="http://www.w3.org/1998/Math/MathML">
          <apply>
            <divide/>
            <ci> mw1f6f8208_3f72_4671_8286_8e080906848d </ci>
            <ci> mwddb6a8f0_b347_4faa_9536_294915a489ea </ci>
          </apply>
        </math>
      </initialAssignment>
      <initialAssignment
symbol="mw895d97df_cb0d_44c8_9785_11b1d6282d01">
        <math xmlns="http://www.w3.org/1998/Math/MathML">
          <apply>
            <divide/>
            <ci> mw27d20453_a8b4_4947_bc96_a82d24a92d58 </ci>
            <ci> mw1968e71e_f5e7_4518_bf96_58e98a32246f </ci>
          </apply>
        </math>
      </initialAssignment>
      <initialAssignment
symbol="mw48d27efc_8ace_4319_b906_4465d77a46fa">
        <math xmlns="http://www.w3.org/1998/Math/MathML">
          <apply>
            <divide/>
            <ci> mwdf582c9f_98b3_4fd2_a1b2_9c10ed295a43 </ci>
            <ci> mwf55bfe7a_0ad3_448f_b02b_5d85aca0aae5 </ci>
          </apply>
        </math>
      </initialAssignment>
      <initialAssignment
symbol="mw46e10e70_4f89_41d2_98d3_04c055feaf2d">
        <math xmlns="http://www.w3.org/1998/Math/MathML">
          <apply>
            <divide/>
            <apply>
              <times/>
              <ci> mwdf582c9f_98b3_4fd2_a1b2_9c10ed295a43 </ci>
              <ci> mwcb443c9f_9d14_4279_aa40_7513a4dd8f48 </ci>
            </apply>
            <ci> mwf55bfe7a_0ad3_448f_b02b_5d85aca0aae5 </ci>
          </apply>
        </math>
      </initialAssignment>
      <initialAssignment
symbol="mw4d33db81_6628_421d_8c61_5ef4a4a647ad">
        <math xmlns="http://www.w3.org/1998/Math/MathML">
          <apply>
            <divide/>
            <ci> mw27d20453_a8b4_4947_bc96_a82d24a92d58 </ci>
            <ci> mw1968e71e_f5e7_4518_bf96_58e98a32246f </ci>
```

```
          </apply>
        </math>
      </initialAssignment>
      <initialAssignment
symbol="mw70a631d4_9f40_4bbb_bb75_a900f1130935">
        <math xmlns="http://www.w3.org/1998/Math/MathML">
          <apply>
            <divide/>
            <ci> mwad42c5a9_e21f_4100_add0_623b9a89169c </ci>
            <ci> mw3a89a2a1_413a_48ba_b39d_abce742943a1 </ci>
          </apply>
        </math>
      </initialAssignment>
      <initialAssignment
symbol="mw5b51513e_0500_4cf8_a613_5a8041e539fd">
        <math xmlns="http://www.w3.org/1998/Math/MathML">
          <apply>
            <divide/>
            <apply>
              <times/>
              <ci> mwad42c5a9_e21f_4100_add0_623b9a89169c </ci>
              <ci> mwe3d9a595_a61c_479a_9ef5_e5a6aa984d65 </ci>
            </apply>
            <ci> mw3a89a2a1_413a_48ba_b39d_abce742943a1 </ci>
          </apply>
        </math>
      </initialAssignment>
      <initialAssignment
symbol="mw5f168fc1_14e9_4b01_916e_4962d6278d2e">
        <math xmlns="http://www.w3.org/1998/Math/MathML">
          <apply>
            <divide/>
            <ci> mwb002721a_80c2_4e13_ab14_bc346c326535 </ci>
            <ci> mw6014386a_19d2_4a74_bb82_c32db3ebe899 </ci>
          </apply>
        </math>
      </initialAssignment>
      <initialAssignment
symbol="mwa5aae57a_8cb6_4abc_8865_078cae23d61b">
        <math xmlns="http://www.w3.org/1998/Math/MathML">
          <apply>
            <divide/>
            <ci> mw9da4d78b_cfec_4e11_b021_8e57ba26a901 </ci>
            <ci> mwd676f62b_ea64_4770_b805_03a42fe744f5 </ci>
          </apply>
        </math>
      </initialAssignment>
      <initialAssignment
symbol="mw16bdbdfb_62b5_45b6_987d_fae9a564f93e">
        <math xmlns="http://www.w3.org/1998/Math/MathML">
          <apply>
            <divide/>
```

```
            <ci> mw8947be8c_bc84_4748_bf0e_d682b86f2ec4 </ci>
            <ci> mw6014386a_19d2_4a74_bb82_c32db3ebe899 </ci>
          </apply>
        </math>
      </initialAssignment>
      <initialAssignment
symbol="mw8cd989f8_196a_44a9_a449_5f7d44e2c0cf">
        <math xmlns="http://www.w3.org/1998/Math/MathML">
          <apply>
            <divide/>
            <ci> mw9da4d78b_cfec_4e11_b021_8e57ba26a901 </ci>
            <ci> mwd676f62b_ea64_4770_b805_03a42fe744f5 </ci>
          </apply>
        </math>
      </initialAssignment>
    </listOfInitialAssignments>
    <listOfRules>
      <assignmentRule
variable="mw175e8610_7578_45f5_80bb_7b61e6e241ab">
        <math xmlns="http://www.w3.org/1998/Math/MathML">
          <apply>
            <plus/>
            <ci> mwa01f7b00_e009_4ea1_a621_7e3a90ebf97a </ci>
            <apply>
              <times/>
              <ci> mwe3f6b99e_81aa_4e82_b8e1_a3ae820e4aa5 </ci>
              <ci> mwdf5f1168_f5b1_4306_8f6d_418a6021e921 </ci>
            </apply>
            <apply>
              <times/>
              <ci> mw2eb1dcb4_cb03_4de1_ae0e_ba7d9c54f167 </ci>
              <ci> mw088a157f_05c9_4257_a674_1b5bb4ee465b </ci>
            </apply>
            <apply>
              <times/>
              <ci> mwe3f6b99e_81aa_4e82_b8e1_a3ae820e4aa5 </ci>
              <ci> mw2aafd721_12db_4909_b572_82abf04c578d </ci>
            </apply>
            <apply>
              <times/>
              <ci> mw2eb1dcb4_cb03_4de1_ae0e_ba7d9c54f167 </ci>
              <ci> mw66344d11_136c_4af3_9f9a_cc5c3035331c </ci>
            </apply>
            <apply>
              <times/>
              <ci> mw2eb1dcb4_cb03_4de1_ae0e_ba7d9c54f167 </ci>
              <ci> mwa5fd10b1_5a9e_41d2_a246_260c6fc1dbf5 </ci>
            </apply>
          </apply>
        </math>
      </assignmentRule>
```

```
      <assignmentRule
variable="mw1e7cee5a_6fd5_4375_9ff9_96509d2f0af7">
        <math xmlns="http://www.w3.org/1998/Math/MathML">
          <apply>
            <plus/>
            <apply>
              <times/>
              <cn type="integer"> 0 </cn>
              <ci> mw5c6752bd_6029_486c_b439_45f7ca1b3111 </ci>
            </apply>
            <ci> mw2aafd721_12db_4909_b572_82abf04c578d </ci>
          </apply>
        </math>
      </assignmentRule>
      <assignmentRule
variable="mw0782fa64_c077_445f_9358_fbb0e8cc0a7e">
        <math xmlns="http://www.w3.org/1998/Math/MathML">
          <apply>
            <plus/>
            <apply>
              <times/>
              <cn type="integer"> 0 </cn>
              <ci> mw5c6752bd_6029_486c_b439_45f7ca1b3111 </ci>
            </apply>
            <ci> mw66344d11_136c_4af3_9f9a_cc5c3035331c </ci>
            <ci> mwa5fd10b1_5a9e_41d2_a246_260c6fc1dbf5 </ci>
          </apply>
        </math>
      </assignmentRule>
      <assignmentRule
variable="mw5e8348dc_3969_4032_bf93_6f838d1e9121">
        <math xmlns="http://www.w3.org/1998/Math/MathML">
          <apply>
            <plus/>
            <apply>
              <times/>
              <cn type="integer"> 0 </cn>
              <ci> mw5c6752bd_6029_486c_b439_45f7ca1b3111 </ci>
            </apply>
            <ci> mw975fec59_e535_46d1_98b4_82ad5cb2c176 </ci>
          </apply>
        </math>
      </assignmentRule>
      <assignmentRule
variable="mwcc034ae1_f0c4_4edf_b337_d36183873773">
        <math xmlns="http://www.w3.org/1998/Math/MathML">
          <apply>
            <plus/>
            <apply>
              <divide/>
              <apply>
                <times/>
```

```
    &lt;cn type="integer"&gt; 0 &lt;/cn&gt;
    &lt;ci&gt; mw5c6752bd_6029_486c_b439_45f7ca1b3111 &lt;/ci&gt;
  &lt;/apply&gt;
  &lt;ci&gt; mwbdd9014c_961d_4199_a03d_419699e06107 &lt;/ci&gt;
&lt;/apply&gt;
&lt;apply&gt;
  &lt;times/&gt;
  &lt;apply&gt;
    &lt;divide/&gt;
    &lt;apply&gt;
      &lt;times/&gt;
      &lt;ci&gt; mw4b836c57_546e_445b_b43d_95ce959cc91a &lt;/ci&gt;
      &lt;ci&gt; mw66344d11_136c_4af3_9f9a_cc5c3035331c &lt;/ci&gt;
      &lt;ci&gt; mw2aafd721_12db_4909_b572_82abf04c578d &lt;/ci&gt;
    &lt;/apply&gt;
    &lt;apply&gt;
      &lt;plus/&gt;
      &lt;ci&gt; mw1e7cee5a_6fd5_4375_9ff9_96509d2f0af7 &lt;/ci&gt;
      &lt;ci&gt; mw0782fa64_c077_445f_9358_fbb0e8cc0a7e &lt;/ci&gt;
      &lt;ci&gt; mw5c6752bd_6029_486c_b439_45f7ca1b3111 &lt;/ci&gt;
    &lt;/apply&gt;
  &lt;/apply&gt;
  &lt;apply&gt;
    &lt;minus/&gt;
    &lt;cn type="integer"&gt; 1 &lt;/cn&gt;
    &lt;ci&gt; mwffa3495a_26ce_48b7_bee5_50af90fed75f &lt;/ci&gt;
  &lt;/apply&gt;
&lt;/apply&gt;
&lt;apply&gt;
  &lt;times/&gt;
  &lt;apply&gt;
    &lt;divide/&gt;
    &lt;apply&gt;
      &lt;times/&gt;
      &lt;ci&gt; mw06785562_a614_40f5_b1cf_23c5c2da8aba &lt;/ci&gt;
      &lt;ci&gt; mw66344d11_136c_4af3_9f9a_cc5c3035331c &lt;/ci&gt;
      &lt;ci&gt; mw2aafd721_12db_4909_b572_82abf04c578d &lt;/ci&gt;
    &lt;/apply&gt;
    &lt;apply&gt;
      &lt;plus/&gt;
      &lt;ci&gt; mw1e7cee5a_6fd5_4375_9ff9_96509d2f0af7 &lt;/ci&gt;
      &lt;ci&gt; mw0782fa64_c077_445f_9358_fbb0e8cc0a7e &lt;/ci&gt;
      &lt;ci&gt; mw5c6752bd_6029_486c_b439_45f7ca1b3111 &lt;/ci&gt;
    &lt;/apply&gt;
  &lt;/apply&gt;
  &lt;ci&gt; mw487f1739_cea4_4ce1_a649_f1ee4097f5aa &lt;/ci&gt;
&lt;/apply&gt;
&lt;apply&gt;
  &lt;times/&gt;
  &lt;apply&gt;
    &lt;divide/&gt;
    &lt;apply&gt;
```

```
                <times/>
                <ci> mw06785562_a614_40f5_b1cf_23c5c2da8aba </ci>
                <ci> mw66344d11_136c_4af3_9f9a_cc5c3035331c </ci>
                <ci> mw2aafd721_12db_4909_b572_82abf04c578d </ci>
              </apply>
              <apply>
                <plus/>
                <ci> mw1e7cee5a_6fd5_4375_9ff9_96509d2f0af7 </ci>
                <ci> mw0782fa64_c077_445f_9358_fbb0e8cc0a7e </ci>
                <ci> mw5c6752bd_6029_486c_b439_45f7ca1b3111 </ci>
              </apply>
            </apply>
            <ci> mwe4443c20_248a_4264_a3dc_ddda03ad2c04 </ci>
          </apply>
        </apply>
      </math>
    </assignmentRule>
    <assignmentRule
variable="mw71812882_d83d_43aa_a957_2dcfc26f16cb">
      <math xmlns="http://www.w3.org/1998/Math/MathML">
        <apply>
          <plus/>
          <ci> mwc101eabc_ad45_48bb_b7ba_0a56d51fed98 </ci>
          <apply>
            <times/>
            <ci> mw4c1ac185_3067_4da9_8869_b8094ae780f1 </ci>
            <ci> mw41e31e9a_6f80_41a3_a437_bfaa6e8baed9 </ci>
          </apply>
          <apply>
            <times/>
            <ci> mw1ba3fe10_7bb4_4be9_9efa_e0e29c841c5b </ci>
            <apply>
              <power/>
              <ci> mw41e31e9a_6f80_41a3_a437_bfaa6e8baed9 </ci>
              <cn type="integer"> 2 </cn>
            </apply>
          </apply>
          <apply>
            <times/>
            <ci> mwabc5258e_86ee_4684_ad1b_022fdf9808b2 </ci>
            <apply>
              <power/>
              <ci> mw41e31e9a_6f80_41a3_a437_bfaa6e8baed9 </ci>
              <cn type="integer"> 3 </cn>
            </apply>
          </apply>
          <apply>
            <times/>
            <ci> mwc8e58a9d_94fd_46ae_88ea_d64803f582e1 </ci>
            <apply>
              <power/>
              <ci> mw41e31e9a_6f80_41a3_a437_bfaa6e8baed9 </ci>
```

```
                <cn type="integer"> 4 </cn>
              </apply>
            </apply>
          </apply>
        </math>
      </assignmentRule>
      <assignmentRule
variable="mwf0b385a0_3c19_4518_9d9f_e58cad44b2ea">
        <math xmlns="http://www.w3.org/1998/Math/MathML">
          <apply>
            <minus/>
            <apply>
              <ln/>
              <apply>
                <divide/>
                <apply>
                  <divide/>
                  <apply>
                    <minus/>
                    <apply>
                      <exp/>
                      <ci> mw41e31e9a_6f80_41a3_a437_bfaa6e8baed9
</ci>
                    </apply>
                    <cn type="integer"> 1 </cn>
                  </apply>
                  <apply>
                    <minus/>
                    <apply>
                      <times/>
                      <cn type="integer"> 2 </cn>
                      <apply>
                        <exp/>
                        <ci> mw41e31e9a_6f80_41a3_a437_bfaa6e8baed9
</ci>
                      </apply>
                    </apply>
                    <cn type="integer"> 1 </cn>
                  </apply>
                </apply>
                <apply>
                  <minus/>
                  <cn type="integer"> 1 </cn>
                  <apply>
                    <exp/>
                    <apply>
                      <minus/>
                      <ci> mw41e31e9a_6f80_41a3_a437_bfaa6e8baed9
</ci>
                    </apply>
                  </apply>
                </apply>
```

```
            </apply>
          </apply>
        </apply>
      </math>
    </assignmentRule>
    <assignmentRule
variable="mwc87bd752_46f1_49c2_b111_e7505fd74ca8">
      <math xmlns="http://www.w3.org/1998/Math/MathML">
        <apply>
          <times/>
          <ci> mwf317e8cb_4357_45f5_b8c5_961986d1cb12 </ci>
          <apply>
            <minus/>
            <cn type="integer"> 1 </cn>
            <apply>
              <times/>
              <apply>
                <minus/>
                <cn type="integer"> 1 </cn>
                <apply>
                  <exp/>
                  <apply>
                    <minus/>
                    <ci> mw41e31e9a_6f80_41a3_a437_bfaa6e8baed9
</ci>
                  </apply>
                </apply>
              </apply>
              <apply>
                <exp/>
                <apply>
                  <times/>
                  <apply>
                    <minus/>
                    <ci> mwf0b385a0_3c19_4518_9d9f_e58cad44b2ea
</ci>
                  </apply>
                  <apply>
                    <power/>
                    <apply>
                      <divide/>
                      <apply>
                        <divide/>
                        <apply>
                          <times/>
                          <ci>
mw36a1fdc2_d829_49d9_8d94_a489fcce2a08 </ci>
                          <ci>
mwf317e8cb_4357_45f5_b8c5_961986d1cb12 </ci>
                        </apply>
                        <apply>
                          <plus/>
```

```
                            <apply>
                              <times/>
                              <ci>
mw154bd5ac_230a_4d71_be6f_5ab6ed5abe16 </ci>
                              <ci>
mwaf05be2a_412f_48e6_8b06_f5d247ba6883 </ci>
                            </apply>
                            <cn type="e-notation"> 1 <sep/> -9 </cn>
                          </apply>
                        </apply>
                        <apply>
                          <minus/>
                          <apply>
                            <times/>
                            <cn type="integer"> 2 </cn>
                            <apply>
                              <exp/>
                              <ci>
mw41e31e9a_6f80_41a3_a437_bfaa6e8baed9 </ci>
                            </apply>
                          </apply>
                          <cn type="integer"> 1 </cn>
                        </apply>
                      </apply>
                      <ci> mw71812882_d83d_43aa_a957_2dcfc26f16cb
</ci>
                    </apply>
                  </apply>
                </apply>
              </apply>
            </apply>
          </apply>
        </math>
      </assignmentRule>
      <assignmentRule
variable="mw41e31e9a_6f80_41a3_a437_bfaa6e8baed9">
        <math xmlns="http://www.w3.org/1998/Math/MathML">
          <apply>
            <plus/>
            <ci> mwb3dbb26b_dc9b_4dae_b56b_52700f65e4e5 </ci>
            <apply>
              <divide/>
              <apply>
                <times/>
                <ci> mwcc68ed97_1448_4aa0_bf3e_a3ce54ccaca8 </ci>
                <ci> mw479632c6_8f17_47aa_b429_216752f11c51 </ci>
              </apply>
              <apply>
                <plus/>
                <ci> mwc266a39c_cd93_4ee9_a52b_c032c6a118f6 </ci>
                <ci> mw479632c6_8f17_47aa_b429_216752f11c51 </ci>
              </apply>
```

```
            </apply>
          </apply>
        </math>
      </assignmentRule>
      <assignmentRule
variable="mwebb3353d_0237_474f_8045_a75656c37891">
        <math xmlns="http://www.w3.org/1998/Math/MathML">
          <ci> mwa5fd10b1_5a9e_41d2_a246_260c6fc1dbf5 </ci>
        </math>
      </assignmentRule>
      <assignmentRule
variable="mwdbdc3427_4618_4a63_be76_98a56e309b17">
        <math xmlns="http://www.w3.org/1998/Math/MathML">
          <apply>
            <times/>
            <ci> mw26ca4282_20b3_4f9b_8ce4_58824cec85fc </ci>
            <apply>
              <minus/>
              <cn type="integer"> 1 </cn>
              <apply>
                <times/>
                <apply>
                  <minus/>
                  <cn type="integer"> 1 </cn>
                  <apply>
                    <exp/>
                    <apply>
                      <minus/>
                      <ci> mw41e31e9a_6f80_41a3_a437_bfaa6e8baed9
</ci>
                    </apply>
                  </apply>
                </apply>
                <apply>
                  <exp/>
                  <apply>
                    <times/>
                    <apply>
                      <minus/>
                      <ci> mwf0b385a0_3c19_4518_9d9f_e58cad44b2ea
</ci>
                    </apply>
                    <apply>
                      <power/>
                      <apply>
                        <divide/>
                        <apply>
                          <divide/>
                          <apply>
                            <times/>
                            <ci>
mwbb1a101c_bd5e_4dd7_b529_54e77169a609 </ci>
```

```
                            &lt;ci&gt;
mw26ca4282_20b3_4f9b_8ce4_58824cec85fc &lt;/ci&gt;
                          &lt;/apply&gt;
                          &lt;apply&gt;
                            &lt;plus/&gt;
                            &lt;apply&gt;
                              &lt;times/&gt;
                              &lt;ci&gt;
mw38ea07af_24e1_43bd_b07b_ec2a1371f4d4 &lt;/ci&gt;
                              &lt;ci&gt;
mw8607693c_2da9_4dd0_9167_ab2793ec2f3a &lt;/ci&gt;
                            &lt;/apply&gt;
                            &lt;cn type="e-notation"&gt; 1 &lt;sep/&gt; -9 &lt;/cn&gt;
                          &lt;/apply&gt;
                        &lt;/apply&gt;
                        &lt;apply&gt;
                          &lt;minus/&gt;
                          &lt;apply&gt;
                            &lt;times/&gt;
                            &lt;cn type="integer"&gt; 2 &lt;/cn&gt;
                            &lt;apply&gt;
                              &lt;exp/&gt;
                              &lt;ci&gt;
mw41e31e9a_6f80_41a3_a437_bfaa6e8baed9 &lt;/ci&gt;
                            &lt;/apply&gt;
                          &lt;/apply&gt;
                          &lt;cn type="integer"&gt; 1 &lt;/cn&gt;
                        &lt;/apply&gt;
                      &lt;/apply&gt;
                      &lt;ci&gt; mw71812882_d83d_43aa_a957_2dcfc26f16cb
&lt;/ci&gt;
                    &lt;/apply&gt;
                  &lt;/apply&gt;
                &lt;/apply&gt;
              &lt;/apply&gt;
            &lt;/apply&gt;
          &lt;/apply&gt;
        &lt;/math&gt;
      &lt;/assignmentRule&gt;
      <assignmentRule
variable="mw38ea07af_24e1_43bd_b07b_ec2a1371f4d4">
        &lt;math xmlns="http://www.w3.org/1998/Math/MathML"&gt;
          &lt;apply&gt;
            &lt;divide/&gt;
            &lt;apply&gt;
              &lt;times/&gt;
              &lt;ci&gt; mw6d000fc0_ba86_41a7_beee_8cafe05139ce &lt;/ci&gt;
              &lt;ci&gt; mw0f3e4986_c5c0_4e85_8f07_03bf0ed91f2b &lt;/ci&gt;
            &lt;/apply&gt;
            &lt;apply&gt;
              &lt;plus/&gt;
              &lt;apply&gt;
```

```
                &lt;times/&gt;
                &lt;ci&gt; mw6d000fc0_ba86_41a7_beee_8cafe05139ce &lt;/ci&gt;
                &lt;ci&gt; mw0f3e4986_c5c0_4e85_8f07_03bf0ed91f2b &lt;/ci&gt;
              &lt;/apply&gt;
              &lt;ci&gt; mw5e8348dc_3969_4032_bf93_6f838d1e9121 &lt;/ci&gt;
              &lt;ci&gt; mw5c6752bd_6029_486c_b439_45f7ca1b3111 &lt;/ci&gt;
            &lt;/apply&gt;
          &lt;/apply&gt;
        &lt;/math&gt;
      &lt;/assignmentRule&gt;
      <assignmentRule
variable="mw154bd5ac_230a_4d71_be6f_5ab6ed5abe16">
        &lt;math xmlns="http://www.w3.org/1998/Math/MathML"&gt;
          &lt;apply&gt;
            &lt;divide/&gt;
            &lt;apply&gt;
              &lt;times/&gt;
              &lt;ci&gt; mw6d000fc0_ba86_41a7_beee_8cafe05139ce &lt;/ci&gt;
              &lt;ci&gt; mw507424c1_49c9_4a06_8493_4e61e9f65e52 &lt;/ci&gt;
            &lt;/apply&gt;
            &lt;apply&gt;
              &lt;plus/&gt;
              &lt;apply&gt;
                &lt;times/&gt;
                &lt;ci&gt; mw6d000fc0_ba86_41a7_beee_8cafe05139ce &lt;/ci&gt;
                &lt;ci&gt; mw507424c1_49c9_4a06_8493_4e61e9f65e52 &lt;/ci&gt;
              &lt;/apply&gt;
              &lt;ci&gt; mw5e8348dc_3969_4032_bf93_6f838d1e9121 &lt;/ci&gt;
              &lt;ci&gt; mw5c6752bd_6029_486c_b439_45f7ca1b3111 &lt;/ci&gt;
            &lt;/apply&gt;
          &lt;/apply&gt;
        &lt;/math&gt;
      &lt;/assignmentRule&gt;
      <assignmentRule
variable="mwb2d4fc83_566f_4b7d_9037_c52ec0a459af">
        &lt;math xmlns="http://www.w3.org/1998/Math/MathML"&gt;
          &lt;apply&gt;
            &lt;times/&gt;
            &lt;cn&gt; 0.5 &lt;/cn&gt;
            &lt;apply&gt;
              &lt;minus/&gt;
              &lt;apply&gt;
                &lt;plus/&gt;
                &lt;apply&gt;
                  &lt;divide/&gt;
                  &lt;ci&gt; mwd1db5e41_695c_4769_b3d8_4151bf3a44d0 &lt;/ci&gt;
                  &lt;ci&gt; mwcdf3bb71_6afa_48e7_b780_2d7ee5f9b5db &lt;/ci&gt;
                &lt;/apply&gt;
                &lt;ci&gt; mw1390963b_ac2e_43bf_9068_18e15a3f8933 &lt;/ci&gt;
                &lt;apply&gt;
                  &lt;divide/&gt;
                  &lt;ci&gt; mwe1d5301d_65a6_4805_a0c4_b9488461ccc8 &lt;/ci&gt;
```

```
                  <ci> mw1bdcde86_7239_49ff_a50c_244b38426b19 </ci>
                </apply>
              </apply>
              <apply>
                <times/>
                <ci> mw1390963b_ac2e_43bf_9068_18e15a3f8933 </ci>
                <apply>
                  <root/>
                  <degree>
                    <cn type="integer"> 2 </cn>
                  </degree>
                  <apply>
                    <minus/>
                    <apply>
                      <power/>
                      <apply>
                        <divide/>
                        <apply>
                          <plus/>
                          <apply>
                            <divide/>
                            <ci>
mwd1db5e41_695c_4769_b3d8_4151bf3a44d0 </ci>
                            <ci>
mwcdf3bb71_6afa_48e7_b780_2d7ee5f9b5db </ci>
                          </apply>
                          <ci> mw1390963b_ac2e_43bf_9068_18e15a3f8933
</ci>
                          <apply>
                            <divide/>
                            <ci>
mwe1d5301d_65a6_4805_a0c4_b9488461ccc8 </ci>
                            <ci>
mw1bdcde86_7239_49ff_a50c_244b38426b19 </ci>
                          </apply>
                        </apply>
                        <ci> mw1390963b_ac2e_43bf_9068_18e15a3f8933
</ci>
                      </apply>
                      <cn type="integer"> 2 </cn>
                    </apply>
                    <apply>
                      <divide/>
                      <apply>
                        <divide/>
                        <apply>
                          <times/>
                          <cn type="integer"> 4 </cn>
                          <ci> mwd1db5e41_695c_4769_b3d8_4151bf3a44d0
</ci>
                        </apply>
```

```
                        <ci> mwcdf3bb71_6afa_48e7_b780_2d7ee5f9b5db
</ci>
                      </apply>
                      <ci> mw1390963b_ac2e_43bf_9068_18e15a3f8933
</ci>
                    </apply>
                  </apply>
                </apply>
              </apply>
            </apply>
          </apply>
        </math>
      </assignmentRule>
      <assignmentRule
variable="mw1e889781_12c4_4172_91b6_546e3a00bfb4">
        <math xmlns="http://www.w3.org/1998/Math/MathML">
          <apply>
            <divide/>
            <ci> mwb2d4fc83_566f_4b7d_9037_c52ec0a459af </ci>
            <apply>
              <plus/>
              <ci> mwb2d4fc83_566f_4b7d_9037_c52ec0a459af </ci>
              <ci> mw45d28c78_e295_422a_93e8_63fd5c065117 </ci>
            </apply>
          </apply>
        </math>
      </assignmentRule>
      <assignmentRule
variable="mw0bcc5b8c_3735_4aa8_be25_eeed6fb095d1">
        <math xmlns="http://www.w3.org/1998/Math/MathML">
          <apply>
            <times/>
            <apply>
              <divide/>
              <ci> mw3e998fd3_0e21_483b_a0dc_f9717401fc48 </ci>
              <apply>
                <plus/>
                <ci> mw3e998fd3_0e21_483b_a0dc_f9717401fc48 </ci>
                <ci> mw0fc88c2e_4d0b_4fc9_ad59_3b84599698f7 </ci>
              </apply>
            </apply>
            <apply>
              <power/>
              <apply>
                <divide/>
                <ci> mw0231f6de_2302_4910_aa44_dbb729a3e732 </ci>
                <apply>
                  <plus/>
                  <ci> mw3e998fd3_0e21_483b_a0dc_f9717401fc48 </ci>
                  <ci> mw0231f6de_2302_4910_aa44_dbb729a3e732 </ci>
                </apply>
              </apply>
```

```
            <ci> mwf5abafbe_c4ea_43d1_917a_1e0f82adcb97 </ci>
          </apply>
          <cn> 0.5 </cn>
          <apply>
            <minus/>
            <apply>
              <plus/>
              <apply>
                <divide/>
                <ci> mwf7546543_1576_4ff3_a1d2_09f9d9d8f508 </ci>
                <ci> mw98c1f539_f992_42f9_b09c_d98aa315dd63 </ci>
              </apply>
              <ci> mw3edb1bd6_42e5_4c85_bd91_3427b4480151 </ci>
              <apply>
                <divide/>
                <ci> mw3e998fd3_0e21_483b_a0dc_f9717401fc48 </ci>
                <ci> mwe857ab2a_b73e_41db_9689_4fce13b4c5f9 </ci>
              </apply>
            </apply>
            <apply>
              <times/>
              <ci> mw3edb1bd6_42e5_4c85_bd91_3427b4480151 </ci>
              <apply>
                <root/>
                <degree>
                  <cn type="integer"> 2 </cn>
                </degree>
                <apply>
                  <minus/>
                  <apply>
                    <power/>
                    <apply>
                      <divide/>
                      <apply>
                        <plus/>
                        <apply>
                          <divide/>
                          <ci>
mwf7546543_1576_4ff3_a1d2_09f9d9d8f508 </ci>
                          <ci>
mw98c1f539_f992_42f9_b09c_d98aa315dd63 </ci>
                        </apply>
                        <ci> mw3edb1bd6_42e5_4c85_bd91_3427b4480151
</ci>
                        <apply>
                          <divide/>
                          <ci>
mw3e998fd3_0e21_483b_a0dc_f9717401fc48 </ci>
                          <ci>
mwe857ab2a_b73e_41db_9689_4fce13b4c5f9 </ci>
                        </apply>
                      </apply>
```

```
                        <ci> mw3edb1bd6_42e5_4c85_bd91_3427b4480151
</ci>
                      </apply>
                      <cn type="integer"> 2 </cn>
                    </apply>
                    <apply>
                      <divide/>
                      <apply>
                        <divide/>
                        <apply>
                          <times/>
                          <cn type="integer"> 4 </cn>
                          <ci> mwf7546543_1576_4ff3_a1d2_09f9d9d8f508
</ci>
                        </apply>
                        <ci> mw98c1f539_f992_42f9_b09c_d98aa315dd63
</ci>
                      </apply>
                      <ci> mw3edb1bd6_42e5_4c85_bd91_3427b4480151
</ci>
                    </apply>
                  </apply>
                </apply>
              </apply>
            </apply>
          </apply>
        </math>
      </assignmentRule>
      <assignmentRule
variable="mw10ec1765_75ce_4474_94ca_6439ea9ef6cd">
        <math xmlns="http://www.w3.org/1998/Math/MathML">
          <apply>
            <divide/>
            <ci> mw0bcc5b8c_3735_4aa8_be25_eeed6fb095d1 </ci>
            <apply>
              <plus/>
              <ci> mw0bcc5b8c_3735_4aa8_be25_eeed6fb095d1 </ci>
              <ci> mw382b7a70_e63f_4a14_b5ae_03012ce36574 </ci>
            </apply>
          </apply>
        </math>
      </assignmentRule>
      <assignmentRule
variable="mw140e5cc4_b897_4719_aa3d_0b30f25bc4dd">
        <math xmlns="http://www.w3.org/1998/Math/MathML">
          <ci> mw59118247_974d_4558_88d8_f2e55f289d1a </ci>
        </math>
      </assignmentRule>
      <assignmentRule
variable="mwffa3495a_26ce_48b7_bee5_50af90fed75f">
        <math xmlns="http://www.w3.org/1998/Math/MathML">
          <apply>
```

```
                <divide/>
                <apply>
                  <power/>
                  <apply>
                    <divide/>
                    <apply>
                      <plus/>
                      <ci> mw538e3c56_061a_417d_9d7f_128d0cd23afa </ci>
                      <ci> mw80d2fce8_7bcd_47a0_8857_df9df2037ea4 </ci>
                    </apply>
                    <ci> mwe2df21f5_c609_4db4_800f_b318a4cd9ebd </ci>
                  </apply>
                  <ci> mw8cda36fb_5234_46ed_bdb8_520a142b8826 </ci>
                </apply>
                <apply>
                  <plus/>
                  <apply>
                    <power/>
                    <apply>
                      <divide/>
                      <apply>
                        <plus/>
                        <ci> mw538e3c56_061a_417d_9d7f_128d0cd23afa </ci>
                        <ci> mw80d2fce8_7bcd_47a0_8857_df9df2037ea4 </ci>
                      </apply>
                      <ci> mwe2df21f5_c609_4db4_800f_b318a4cd9ebd </ci>
                    </apply>
                    <ci> mw8cda36fb_5234_46ed_bdb8_520a142b8826 </ci>
                  </apply>
                  <cn type="integer"> 1 </cn>
                </apply>
              </apply>
            </math>
          </assignmentRule>
          <assignmentRule
variable="mw5fab6ae8_fb41_4c3a_818e_ce71aaaac5d9">
            <math xmlns="http://www.w3.org/1998/Math/MathML">
              <apply>
                <divide/>
                <apply>
                  <power/>
                  <apply>
                    <divide/>
                    <apply>
                      <plus/>
                      <ci> mwc47a95ad_5c77_4a62_b9b7_cb2b8abe3260 </ci>
                      <ci> mw345e02b4_624f_415f_af98_e55a9f555853 </ci>
                    </apply>
                    <ci> mwe2df21f5_c609_4db4_800f_b318a4cd9ebd </ci>
                  </apply>
                  <ci> mw8cda36fb_5234_46ed_bdb8_520a142b8826 </ci>
                </apply>
```

```
              &lt;apply&gt;
                &lt;plus/&gt;
                &lt;apply&gt;
                  &lt;power/&gt;
                  &lt;apply&gt;
                    &lt;divide/&gt;
                    &lt;apply&gt;
                      &lt;plus/&gt;
                      &lt;ci&gt; mwc47a95ad_5c77_4a62_b9b7_cb2b8abe3260 &lt;/ci&gt;
                      &lt;ci&gt; mw345e02b4_624f_415f_af98_e55a9f555853 &lt;/ci&gt;
                    &lt;/apply&gt;
                    &lt;ci&gt; mwe2df21f5_c609_4db4_800f_b318a4cd9ebd &lt;/ci&gt;
                  &lt;/apply&gt;
                  &lt;ci&gt; mw8cda36fb_5234_46ed_bdb8_520a142b8826 &lt;/ci&gt;
                &lt;/apply&gt;
                &lt;cn type="integer"&gt; 1 &lt;/cn&gt;
              &lt;/apply&gt;
            &lt;/apply&gt;
          &lt;/math&gt;
        &lt;/assignmentRule&gt;
        <assignmentRule
variable="mw487f1739_cea4_4ce1_a649_f1ee4097f5aa">
          &lt;math xmlns="http://www.w3.org/1998/Math/MathML"&gt;
            &lt;apply&gt;
              &lt;divide/&gt;
              &lt;apply&gt;
                &lt;power/&gt;
                &lt;apply&gt;
                  &lt;divide/&gt;
                  &lt;ci&gt; mwf1568170_3b99_448b_94b1_035796c05f2f &lt;/ci&gt;
                  &lt;ci&gt; mwaecb4a00_67ae_4818_8f41_195a5060534e &lt;/ci&gt;
                &lt;/apply&gt;
                &lt;ci&gt; mwdbca445b_0370_40eb_9cb0_41018b8b2740 &lt;/ci&gt;
              &lt;/apply&gt;
              &lt;apply&gt;
                &lt;plus/&gt;
                &lt;apply&gt;
                  &lt;power/&gt;
                  &lt;apply&gt;
                    &lt;divide/&gt;
                    &lt;ci&gt; mwf1568170_3b99_448b_94b1_035796c05f2f &lt;/ci&gt;
                    &lt;ci&gt; mwaecb4a00_67ae_4818_8f41_195a5060534e &lt;/ci&gt;
                  &lt;/apply&gt;
                  &lt;ci&gt; mwdbca445b_0370_40eb_9cb0_41018b8b2740 &lt;/ci&gt;
                &lt;/apply&gt;
                &lt;cn type="integer"&gt; 1 &lt;/cn&gt;
              &lt;/apply&gt;
            &lt;/apply&gt;
          &lt;/math&gt;
        &lt;/assignmentRule&gt;
        <assignmentRule
variable="mwe4443c20_248a_4264_a3dc_ddda03ad2c04">
```

```
          <math xmlns="http://www.w3.org/1998/Math/MathML">
            <apply>
              <divide/>
              <apply>
                <power/>
                <apply>
                  <divide/>
                  <ci> mw5c8acb40_e149_4324_a7a2_eadf8fda3445 </ci>
                  <ci> mwaecb4a00_67ae_4818_8f41_195a5060534e </ci>
                </apply>
                <ci> mwdbca445b_0370_40eb_9cb0_41018b8b2740 </ci>
              </apply>
              <apply>
                <plus/>
                <apply>
                  <power/>
                  <apply>
                    <divide/>
                    <ci> mw5c8acb40_e149_4324_a7a2_eadf8fda3445 </ci>
                    <ci> mwaecb4a00_67ae_4818_8f41_195a5060534e </ci>
                  </apply>
                  <ci> mwdbca445b_0370_40eb_9cb0_41018b8b2740 </ci>
                </apply>
                <cn type="integer"> 1 </cn>
              </apply>
            </apply>
          </math>
        </assignmentRule>
      </listOfRules>
      <listOfReactions>
        <reaction id="mw9907037b_d4c8_4042_95fd_de9d27584cb7"
reversible="false" fast="false">
          <listOfReactants>
            <speciesReference
species="mwdf5f1168_f5b1_4306_8f6d_418a6021e921" stoichiometry="1"/>
          </listOfReactants>
          <kineticLaw>
            <math xmlns="http://www.w3.org/1998/Math/MathML">
              <apply>
                <times/>
                <ci> mw1ab76cfe_e766_4ab0_b0c5_5162010849d6 </ci>
                <ci> mwdf5f1168_f5b1_4306_8f6d_418a6021e921 </ci>
              </apply>
            </math>
          </kineticLaw>
        </reaction>
        <reaction id="mwdd7621f0_a6af_47ee_8304_4ad76542d794"
reversible="false" fast="false">
          <listOfReactants>
            <speciesReference
species="mw088a157f_05c9_4257_a674_1b5bb4ee465b" stoichiometry="1"/>
          </listOfReactants>
```

```
        <kineticLaw>
          <math xmlns="http://www.w3.org/1998/Math/MathML">
            <apply>
              <times/>
              <ci> mw1ab76cfe_e766_4ab0_b0c5_5162010849d6 </ci>
              <ci> mw088a157f_05c9_4257_a674_1b5bb4ee465b </ci>
            </apply>
          </math>
        </kineticLaw>
      </reaction>
      <reaction id="mwf2b4ce6b_e3c7_4ab7_82c8_ee85336af5b5"
reversible="false" fast="false">
        <notes>
          <body xmlns="http://www.w3.org/1999/xhtml">Cancer cell
growth (Logistic model based on Komarova and reviewed in
Marusic)</body>
        </notes>
        <listOfProducts>
          <speciesReference
species="mw2aafd721_12db_4909_b572_82abf04c578d" stoichiometry="1"/>
        </listOfProducts>
        <kineticLaw>
          <math xmlns="http://www.w3.org/1998/Math/MathML">
            <apply>
              <times/>
              <ci> mw79e9ce52_7313_4287_ad50_9c9cf1274898 </ci>
              <ci> mw2aafd721_12db_4909_b572_82abf04c578d </ci>
              <apply>
                <minus/>
                <cn type="integer"> 1 </cn>
                <apply>
                  <divide/>
                  <ci> mw1e7cee5a_6fd5_4375_9ff9_96509d2f0af7 </ci>
                  <ci> mwedddfe92_4421_4f29_8a47_d1b8a6db2304 </ci>
                </apply>
              </apply>
            </apply>
          </math>
        </kineticLaw>
      </reaction>
      <reaction id="mw004be998_012f_4dec_961b_1046b2680c7f"
reversible="false" fast="false">
        <notes>
          <body xmlns="http://www.w3.org/1999/xhtml">Cancer cell
death</body>
        </notes>
        <listOfReactants>
          <speciesReference
species="mw2aafd721_12db_4909_b572_82abf04c578d" stoichiometry="1"/>
        </listOfReactants>
        <listOfProducts>
```

```
          <speciesReference
species="mwdf5f1168_f5b1_4306_8f6d_418a6021e921" stoichiometry="1"/>
        </listOfProducts>
        <kineticLaw>
          <math xmlns="http://www.w3.org/1998/Math/MathML">
            <apply>
              <times/>
              <ci> mw92cb2786_b098_48c2_a8dd_18d26e65a0d6 </ci>
              <ci> mw2aafd721_12db_4909_b572_82abf04c578d </ci>
            </apply>
          </math>
        </kineticLaw>
      </reaction>
      <reaction id="mw5e912079_9687_45d0_b5d3_29824277c92d"
reversible="false" fast="false">
        <notes>
          <body xmlns="http://www.w3.org/1999/xhtml">Naive T cell
entry into the lymph node</body>
        </notes>
        <listOfProducts>
          <speciesReference
species="mwaf05be2a_412f_48e6_8b06_f5d247ba6883" stoichiometry="1"/>
        </listOfProducts>
        <kineticLaw>
          <math xmlns="http://www.w3.org/1998/Math/MathML">
            <apply>
              <times/>
              <ci> mw6bb44ac8_1770_41f1_ab1d_0d1a00772b9a </ci>
              <ci> mw98c1f539_f992_42f9_b09c_d98aa315dd63 </ci>
            </apply>
          </math>
        </kineticLaw>
      </reaction>
      <reaction id="mwb082dcbc_2f59_49e3_aebe_92b5e5f2d2ff"
reversible="false" fast="false">
        <notes>
          <body xmlns="http://www.w3.org/1999/xhtml">Naive T cell exit
from the lymph node</body>
        </notes>
        <listOfReactants>
          <speciesReference
species="mwaf05be2a_412f_48e6_8b06_f5d247ba6883" stoichiometry="1"/>
        </listOfReactants>
        <kineticLaw>
          <math xmlns="http://www.w3.org/1998/Math/MathML">
            <apply>
              <times/>
              <ci> mwe7749870_048b_4ac0_844b_ef279fe1561f </ci>
              <ci> mwaf05be2a_412f_48e6_8b06_f5d247ba6883 </ci>
            </apply>
          </math>
        </kineticLaw>
```

```
      </reaction>
      <reaction id="mw38511581_c07d_435b_b8bc_c5e6c5500525"
reversible="false" fast="false">
        <notes>
          <body xmlns="http://www.w3.org/1999/xhtml">Naive T cell
activation</body>
        </notes>
        <listOfReactants>
          <speciesReference
species="mwaf05be2a_412f_48e6_8b06_f5d247ba6883" stoichiometry="1"/>
        </listOfReactants>
        <listOfProducts>
          <speciesReference
species="mwf317e8cb_4357_45f5_b8c5_961986d1cb12" stoichiometry="1"/>
        </listOfProducts>
        <kineticLaw>
          <math xmlns="http://www.w3.org/1998/Math/MathML">
            <apply>
              <times/>
              <ci> mwbebade6f_8329_4441_8a17_da5f70d2cb42 </ci>
              <ci> mw154bd5ac_230a_4d71_be6f_5ab6ed5abe16 </ci>
              <ci> mw10ec1765_75ce_4474_94ca_6439ea9ef6cd </ci>
              <ci> mwaf05be2a_412f_48e6_8b06_f5d247ba6883 </ci>
            </apply>
          </math>
        </kineticLaw>
      </reaction>
      <reaction id="mw229b1d7f_f0b3_4c0a_95f1_34898be9de2c"
reversible="false" fast="false">
        <notes>
          <body xmlns="http://www.w3.org/1999/xhtml">aT1 cell
proliferation</body>
        </notes>
        <listOfReactants>
          <speciesReference
species="mwf317e8cb_4357_45f5_b8c5_961986d1cb12" stoichiometry="1"/>
        </listOfReactants>
        <kineticLaw>
          <math xmlns="http://www.w3.org/1998/Math/MathML">
            <apply>
              <times/>
              <apply>
                <divide/>
                <ci> mw2c17a42d_4cb1_43ab_90b6_6d1dbadc56de </ci>
                <ci> mw41e31e9a_6f80_41a3_a437_bfaa6e8baed9 </ci>
              </apply>
              <ci> mwf317e8cb_4357_45f5_b8c5_961986d1cb12 </ci>
            </apply>
          </math>
        </kineticLaw>
      </reaction>
```

```
      <reaction id="mw2159c589_6696_48b9_92cb_80fa4c0684c8"
reversible="false" fast="false">
        <notes>
          <body xmlns="http://www.w3.org/1999/xhtml">aT1 cell
proliferation</body>
        </notes>
        <listOfProducts>
          <speciesReference
species="mw975fec59_e535_46d1_98b4_82ad5cb2c176" stoichiometry="1"/>
        </listOfProducts>
        <kineticLaw>
          <math xmlns="http://www.w3.org/1998/Math/MathML">
            <apply>
              <times/>
              <apply>
                <divide/>
                <ci> mw2c17a42d_4cb1_43ab_90b6_6d1dbadc56de </ci>
                <ci> mw41e31e9a_6f80_41a3_a437_bfaa6e8baed9 </ci>
              </apply>
              <cn type="integer"> 2 </cn>
              <apply>
                <exp/>
                <apply>
                  <minus/>
                  <ci> mw41e31e9a_6f80_41a3_a437_bfaa6e8baed9 </ci>
                  <cn type="integer"> 1 </cn>
                </apply>
              </apply>
              <ci> mwf317e8cb_4357_45f5_b8c5_961986d1cb12 </ci>
              <apply>
                <minus/>
                <cn type="integer"> 1 </cn>
                <ci> mw5fab6ae8_fb41_4c3a_818e_ce71aaaac5d9 </ci>
              </apply>
            </apply>
          </math>
        </kineticLaw>
      </reaction>
      <reaction id="mw06b6b81d_375a_4784_8c97_05e10e8b4f98"
reversible="false" fast="false">
        <notes>
          <body xmlns="http://www.w3.org/1999/xhtml">T cell death in
the central compartment</body>
        </notes>
        <listOfReactants>
          <speciesReference
species="mwa04d3906_d300_4e70_bbc1_fb52680cd5d3" stoichiometry="1"/>
        </listOfReactants>
        <kineticLaw>
          <math xmlns="http://www.w3.org/1998/Math/MathML">
            <apply>
              <times/>
```

```
              <ci> mw9bcacce9_c511_4740_973e_f50fb674fd80 </ci>
              <ci> mwa04d3906_d300_4e70_bbc1_fb52680cd5d3 </ci>
            </apply>
          </math>
        </kineticLaw>
      </reaction>
      <reaction id="mwd2109bf4_1aaa_4aad_9e82_2027ca627c14"
reversible="false" fast="false">
        <notes>
          <body xmlns="http://www.w3.org/1999/xhtml">T cell death in
the peripheral compartment</body>
        </notes>
        <listOfReactants>
          <speciesReference
species="mw2c87cd24_8cfc_4eb0_8b60_5565ba85c240" stoichiometry="1"/>
        </listOfReactants>
        <kineticLaw>
          <math xmlns="http://www.w3.org/1998/Math/MathML">
            <apply>
              <times/>
              <ci> mw9bcacce9_c511_4740_973e_f50fb674fd80 </ci>
              <ci> mw2c87cd24_8cfc_4eb0_8b60_5565ba85c240 </ci>
            </apply>
          </math>
        </kineticLaw>
      </reaction>
      <reaction id="mw1a2b5299_6c86_4e9f_80f7_18baf211069f"
reversible="false" fast="false">
        <notes>
          <body xmlns="http://www.w3.org/1999/xhtml">T cell death in
the tumour compartment</body>
        </notes>
        <listOfReactants>
          <speciesReference
species="mw66344d11_136c_4af3_9f9a_cc5c3035331c" stoichiometry="1"/>
        </listOfReactants>
        <listOfProducts>
          <speciesReference
species="mw088a157f_05c9_4257_a674_1b5bb4ee465b" stoichiometry="1"/>
        </listOfProducts>
        <kineticLaw>
          <math xmlns="http://www.w3.org/1998/Math/MathML">
            <apply>
              <times/>
              <ci> mw9bcacce9_c511_4740_973e_f50fb674fd80 </ci>
              <ci> mw66344d11_136c_4af3_9f9a_cc5c3035331c </ci>
            </apply>
          </math>
        </kineticLaw>
      </reaction>
      <reaction id="mw1bf0546b_7c0c_4ef3_8554_b996d9aaec4a"
reversible="false" fast="false">
```

```
        <notes>
          <body xmlns="http://www.w3.org/1999/xhtml">T cell death in
the lymph node compartment</body>
        </notes>
        <listOfReactants>
          <speciesReference
species="mw975fec59_e535_46d1_98b4_82ad5cb2c176" stoichiometry="1"/>
        </listOfReactants>
        <kineticLaw>
          <math xmlns="http://www.w3.org/1998/Math/MathML">
            <apply>
              <times/>
              <ci> mw9bcacce9_c511_4740_973e_f50fb674fd80 </ci>
              <ci> mw975fec59_e535_46d1_98b4_82ad5cb2c176 </ci>
            </apply>
          </math>
        </kineticLaw>
      </reaction>
      <reaction id="mw91106aa9_b104_4611_bb7a_562e1525944a"
reversible="false" fast="false">
        <notes>
          <body xmlns="http://www.w3.org/1999/xhtml">T cell death from
Tregs</body>
        </notes>
        <listOfReactants>
          <speciesReference
species="mw66344d11_136c_4af3_9f9a_cc5c3035331c" stoichiometry="1"/>
        </listOfReactants>
        <listOfProducts>
          <speciesReference
species="mw088a157f_05c9_4257_a674_1b5bb4ee465b" stoichiometry="1"/>
        </listOfProducts>
        <kineticLaw>
          <math xmlns="http://www.w3.org/1998/Math/MathML">
            <apply>
              <divide/>
              <apply>
                <times/>
                <ci> mw8108013c_5caa_4887_9636_3b1da28004b8 </ci>
                <ci> mw66344d11_136c_4af3_9f9a_cc5c3035331c </ci>
                <ci> mwebb3353d_0237_474f_8045_a75656c37891 </ci>
              </apply>
              <apply>
                <plus/>
                <ci> mw1e7cee5a_6fd5_4375_9ff9_96509d2f0af7 </ci>
                <ci> mw0782fa64_c077_445f_9358_fbb0e8cc0a7e </ci>
                <ci> mw5c6752bd_6029_486c_b439_45f7ca1b3111 </ci>
              </apply>
            </apply>
          </math>
        </kineticLaw>
      </reaction>
```

```
      <reaction id="mw675e6b40_11c7_494b_bdd3_aa4ce6a01a8a"
reversible="false" fast="false">
        <notes>
          <body xmlns="http://www.w3.org/1999/xhtml">T cell death from
Tregs</body>
        </notes>
        <listOfReactants>
          <speciesReference
species="mw66344d11_136c_4af3_9f9a_cc5c3035331c" stoichiometry="1"/>
        </listOfReactants>
        <listOfProducts>
          <speciesReference
species="mw088a157f_05c9_4257_a674_1b5bb4ee465b" stoichiometry="1"/>
        </listOfProducts>
        <kineticLaw>
          <math xmlns="http://www.w3.org/1998/Math/MathML">
            <apply>
              <times/>
              <apply>
                <divide/>
                <apply>
                  <times/>
                  <ci> mw5c913adf_4222_49ab_bf00_936c2cac430d </ci>
                  <ci> mw66344d11_136c_4af3_9f9a_cc5c3035331c </ci>
                  <ci> mwebb3353d_0237_474f_8045_a75656c37891 </ci>
                </apply>
                <apply>
                  <plus/>
                  <ci> mw1e7cee5a_6fd5_4375_9ff9_96509d2f0af7 </ci>
                  <ci> mw0782fa64_c077_445f_9358_fbb0e8cc0a7e </ci>
                  <ci> mw5c6752bd_6029_486c_b439_45f7ca1b3111 </ci>
                </apply>
              </apply>
              <ci> mwe4443c20_248a_4264_a3dc_ddda03ad2c04 </ci>
            </apply>
          </math>
        </kineticLaw>
      </reaction>
      <reaction id="mw3fc411e1_31a7_4219_938e_5c5f6243374c"
reversible="false" fast="false">
        <notes>
          <body xmlns="http://www.w3.org/1999/xhtml">T cell death from
cancer</body>
        </notes>
        <listOfReactants>
          <speciesReference
species="mw66344d11_136c_4af3_9f9a_cc5c3035331c" stoichiometry="1"/>
        </listOfReactants>
        <listOfProducts>
          <speciesReference
species="mw088a157f_05c9_4257_a674_1b5bb4ee465b" stoichiometry="1"/>
        </listOfProducts>
```

```
        <kineticLaw>
          <math xmlns="http://www.w3.org/1998/Math/MathML">
            <apply>
              <times/>
              <apply>
                <divide/>
                <apply>
                  <times/>
                  <ci> mw7dd2d096_125c_43c8_8c6d_462231267034 </ci>
                  <ci> mw66344d11_136c_4af3_9f9a_cc5c3035331c </ci>
                  <ci> mw1e7cee5a_6fd5_4375_9ff9_96509d2f0af7 </ci>
                </apply>
                <apply>
                  <plus/>
                  <ci> mw1e7cee5a_6fd5_4375_9ff9_96509d2f0af7 </ci>
                  <ci> mw0782fa64_c077_445f_9358_fbb0e8cc0a7e </ci>
                  <ci> mw5c6752bd_6029_486c_b439_45f7ca1b3111 </ci>
                </apply>
              </apply>
              <ci> mwffa3495a_26ce_48b7_bee5_50af90fed75f </ci>
            </apply>
          </math>
        </kineticLaw>
      </reaction>
      <reaction id="mw6203457e_2d4d_467c_a21e_8bc36c193a39"
reversible="false" fast="false">
        <notes>
          <body xmlns="http://www.w3.org/1999/xhtml">T cell transport
into the peripheral compartment</body>
        </notes>
        <listOfReactants>
          <speciesReference
species="mwa04d3906_d300_4e70_bbc1_fb52680cd5d3" stoichiometry="1"/>
        </listOfReactants>
        <listOfProducts>
          <speciesReference
species="mw2c87cd24_8cfc_4eb0_8b60_5565ba85c240" stoichiometry="1"/>
        </listOfProducts>
        <kineticLaw>
          <math xmlns="http://www.w3.org/1998/Math/MathML">
            <apply>
              <times/>
              <ci> mw73b6655b_bc81_420b_bef6_4a23b4c57207 </ci>
              <ci> mwa04d3906_d300_4e70_bbc1_fb52680cd5d3 </ci>
            </apply>
          </math>
        </kineticLaw>
      </reaction>
      <reaction id="mw435ccd06_8191_4aa8_8235_470e1a71c243"
reversible="false" fast="false">
        <notes>
```

```
          <body xmlns="http://www.w3.org/1999/xhtml">T cell transport
out of the peripheral compartment</body>
        </notes>
        <listOfReactants>
          <speciesReference
species="mw2c87cd24_8cfc_4eb0_8b60_5565ba85c240" stoichiometry="1"/>
        </listOfReactants>
        <listOfProducts>
          <speciesReference
species="mwa04d3906_d300_4e70_bbc1_fb52680cd5d3" stoichiometry="1"/>
        </listOfProducts>
        <kineticLaw>
          <math xmlns="http://www.w3.org/1998/Math/MathML">
            <apply>
              <times/>
              <ci> mwdc728989_1d46_4fe0_bc40_67ec5556584c </ci>
              <ci> mw2c87cd24_8cfc_4eb0_8b60_5565ba85c240 </ci>
            </apply>
          </math>
        </kineticLaw>
      </reaction>
      <reaction id="mw9ca5bf66_1455_4abd_953f_6b72e52cc63f"
reversible="false" fast="false">
        <notes>
          <body xmlns="http://www.w3.org/1999/xhtml">T cell transport
into the tumour compartment</body>
        </notes>
        <listOfReactants>
          <speciesReference
species="mwa04d3906_d300_4e70_bbc1_fb52680cd5d3" stoichiometry="1"/>
        </listOfReactants>
        <listOfProducts>
          <speciesReference
species="mw66344d11_136c_4af3_9f9a_cc5c3035331c" stoichiometry="1"/>
        </listOfProducts>
        <kineticLaw>
          <math xmlns="http://www.w3.org/1998/Math/MathML">
            <apply>
              <times/>
              <ci> mw2fc20c96_b91f_40c9_b552_5aae17078d51 </ci>
              <ci> mw175e8610_7578_45f5_80bb_7b61e6e241ab </ci>
              <ci> mwa04d3906_d300_4e70_bbc1_fb52680cd5d3 </ci>
            </apply>
          </math>
        </kineticLaw>
      </reaction>
      <reaction id="mw05644126_4cf9_4148_940d_a7d463aaa367"
reversible="false" fast="false">
        <notes>
          <body xmlns="http://www.w3.org/1999/xhtml">T cell transport
out of the lymph node compartment</body>
        </notes>
```

```
        <listOfReactants>
          <speciesReference
species="mw975fec59_e535_46d1_98b4_82ad5cb2c176" stoichiometry="1"/>
        </listOfReactants>
        <listOfProducts>
          <speciesReference
species="mwa04d3906_d300_4e70_bbc1_fb52680cd5d3" stoichiometry="1"/>
        </listOfProducts>
        <kineticLaw>
          <math xmlns="http://www.w3.org/1998/Math/MathML">
            <apply>
              <times/>
              <ci> mwecabb6dc_07d2_452b_b559_7a5a9c36e194 </ci>
              <ci> mw975fec59_e535_46d1_98b4_82ad5cb2c176 </ci>
            </apply>
          </math>
        </kineticLaw>
      </reaction>
      <reaction id="mw2209469c_a716_42b1_9021_776efdfea374"
reversible="false" fast="false">
        <notes>
          <body xmlns="http://www.w3.org/1999/xhtml">IL2
degradation</body>
        </notes>
        <listOfReactants>
          <speciesReference
species="mw479632c6_8f17_47aa_b429_216752f11c51" stoichiometry="1"/>
        </listOfReactants>
        <kineticLaw>
          <math xmlns="http://www.w3.org/1998/Math/MathML">
            <apply>
              <times/>
              <ci> mw8f2a737b_6631_4e62_834c_0e2ad84cb12a </ci>
              <ci> mw479632c6_8f17_47aa_b429_216752f11c51 </ci>
              <ci> mwb4901521_e24a_4f39_acff_107e3522faa9 </ci>
            </apply>
          </math>
        </kineticLaw>
      </reaction>
      <reaction id="mw2a56ee6d_b146_49de_a3b6_0a165003a0d5"
reversible="false" fast="false">
        <notes>
          <body xmlns="http://www.w3.org/1999/xhtml">IL2 consumption
by T cells</body>
        </notes>
        <listOfReactants>
          <speciesReference
species="mw479632c6_8f17_47aa_b429_216752f11c51" stoichiometry="1"/>
        </listOfReactants>
        <kineticLaw>
          <math xmlns="http://www.w3.org/1998/Math/MathML">
            <apply>
```

```
            <divide/>
            <apply>
              <times/>
              <ci> mw4db95ce7_153f_4c52_b346_bb639b69966e </ci>
              <ci> mw5e8348dc_3969_4032_bf93_6f838d1e9121 </ci>
              <ci> mw479632c6_8f17_47aa_b429_216752f11c51 </ci>
            </apply>
            <apply>
              <plus/>
              <ci> mwc266a39c_cd93_4ee9_a52b_c032c6a118f6 </ci>
              <ci> mw479632c6_8f17_47aa_b429_216752f11c51 </ci>
            </apply>
          </apply>
        </math>
      </kineticLaw>
    </reaction>
    <reaction id="mw6de1b507_489a_4b5c_8951_fbb0ce748dfc"
reversible="false" fast="false">
      <notes>
        <body xmlns="http://www.w3.org/1999/xhtml">IL2 secretion
from activated T cells</body>
      </notes>
      <listOfProducts>
        <speciesReference
species="mw479632c6_8f17_47aa_b429_216752f11c51" stoichiometry="1"/>
      </listOfProducts>
      <kineticLaw>
        <math xmlns="http://www.w3.org/1998/Math/MathML">
          <apply>
            <times/>
            <ci> mwa77be588_4158_477f_b088_450a8a14647d </ci>
            <ci> mwf317e8cb_4357_45f5_b8c5_961986d1cb12 </ci>
          </apply>
        </math>
      </kineticLaw>
    </reaction>
    <reaction id="mwf159d4cc_113a_4cba_b579_8fbf09a017dd"
reversible="false" fast="false">
      <notes>
        <body xmlns="http://www.w3.org/1999/xhtml">Cancer cell
killing by T cells</body>
      </notes>
      <listOfReactants>
        <speciesReference
species="mw2aafd721_12db_4909_b572_82abf04c578d" stoichiometry="1"/>
      </listOfReactants>
      <listOfProducts>
        <speciesReference
species="mwdf5f1168_f5b1_4306_8f6d_418a6021e921" stoichiometry="1"/>
      </listOfProducts>
      <kineticLaw>
        <math xmlns="http://www.w3.org/1998/Math/MathML">
```

```
<apply>
  <plus/>
  <apply>
    <times/>
    <apply>
      <divide/>
      <apply>
        <times/>
        <ci> mw4b836c57_546e_445b_b43d_95ce959cc91a </ci>
        <ci> mw66344d11_136c_4af3_9f9a_cc5c3035331c </ci>
        <ci> mw2aafd721_12db_4909_b572_82abf04c578d </ci>
      </apply>
      <apply>
        <plus/>
        <ci> mw1e7cee5a_6fd5_4375_9ff9_96509d2f0af7 </ci>
        <ci> mw0782fa64_c077_445f_9358_fbb0e8cc0a7e </ci>
        <ci> mw5c6752bd_6029_486c_b439_45f7ca1b3111 </ci>
      </apply>
    </apply>
    <apply>
      <minus/>
      <cn type="integer"> 1 </cn>
      <ci> mwffa3495a_26ce_48b7_bee5_50af90fed75f </ci>
    </apply>
  </apply>
  <apply>
    <times/>
    <apply>
      <divide/>
      <apply>
        <times/>
        <ci> mw06785562_a614_40f5_b1cf_23c5c2da8aba </ci>
        <ci> mw66344d11_136c_4af3_9f9a_cc5c3035331c </ci>
        <ci> mw2aafd721_12db_4909_b572_82abf04c578d </ci>
      </apply>
      <apply>
        <plus/>
        <ci> mw1e7cee5a_6fd5_4375_9ff9_96509d2f0af7 </ci>
        <ci> mw0782fa64_c077_445f_9358_fbb0e8cc0a7e </ci>
        <ci> mw5c6752bd_6029_486c_b439_45f7ca1b3111 </ci>
      </apply>
    </apply>
    <ci> mw487f1739_cea4_4ce1_a649_f1ee4097f5aa </ci>
  </apply>
  <apply>
    <times/>
    <apply>
      <divide/>
      <apply>
        <times/>
        <ci> mwcd6f2806_c6ac_486c_b680_913fb6fc077b </ci>
        <ci> mw66344d11_136c_4af3_9f9a_cc5c3035331c </ci>
```

```
                  <ci> mw2aafd721_12db_4909_b572_82abf04c578d </ci>
                </apply>
                <apply>
                  <plus/>
                  <ci> mw1e7cee5a_6fd5_4375_9ff9_96509d2f0af7 </ci>
                  <ci> mw0782fa64_c077_445f_9358_fbb0e8cc0a7e </ci>
                  <ci> mw5c6752bd_6029_486c_b439_45f7ca1b3111 </ci>
                </apply>
              </apply>
              <ci> mwe4443c20_248a_4264_a3dc_ddda03ad2c04 </ci>
            </apply>
          </apply>
        </math>
      </kineticLaw>
    </reaction>
    <reaction id="mw37fa7a4f_3b5b_40ea_9fd7_ed05431e13e0"
reversible="false" fast="false">
      <notes>
        <body xmlns="http://www.w3.org/1999/xhtml">Naive T cell
entry into the lymph node</body>
      </notes>
      <listOfProducts>
        <speciesReference
species="mw8607693c_2da9_4dd0_9167_ab2793ec2f3a" stoichiometry="1"/>
      </listOfProducts>
      <kineticLaw>
        <math xmlns="http://www.w3.org/1998/Math/MathML">
          <apply>
            <times/>
            <ci> mw43bc1fbc_527c_4caa_b5f0_dacb9c792a18 </ci>
            <ci> mwcdf3bb71_6afa_48e7_b780_2d7ee5f9b5db </ci>
          </apply>
        </math>
      </kineticLaw>
    </reaction>
    <reaction id="mw26e70624_211d_4d90_be49_513ec4e40239"
reversible="false" fast="false">
      <notes>
        <body xmlns="http://www.w3.org/1999/xhtml">Naive T cell exit
from the lymph node</body>
      </notes>
      <listOfReactants>
        <speciesReference
species="mw8607693c_2da9_4dd0_9167_ab2793ec2f3a" stoichiometry="1"/>
      </listOfReactants>
      <kineticLaw>
        <math xmlns="http://www.w3.org/1998/Math/MathML">
          <apply>
            <times/>
            <ci> mw6657d584_3305_4159_9028_0fd387db86bc </ci>
            <ci> mw8607693c_2da9_4dd0_9167_ab2793ec2f3a </ci>
          </apply>
```

```
          &lt;/math&gt;
        &lt;/kineticLaw&gt;
      &lt;/reaction&gt;
      <reaction id="mweb662ab9_d1df_481b_9500_899b514f8007"
reversible="false" fast="false">
        &lt;notes&gt;
          &lt;body xmlns="http://www.w3.org/1999/xhtml"&gt;Naive T cell
activation&lt;/body&gt;
        &lt;/notes&gt;
        &lt;listOfReactants&gt;
          <speciesReference
species="mw8607693c_2da9_4dd0_9167_ab2793ec2f3a" stoichiometry="1"/>
        &lt;/listOfReactants&gt;
        &lt;listOfProducts&gt;
          <speciesReference
species="mw26ca4282_20b3_4f9b_8ce4_58824cec85fc" stoichiometry="1"/>
        &lt;/listOfProducts&gt;
        &lt;kineticLaw&gt;
          &lt;math xmlns="http://www.w3.org/1998/Math/MathML"&gt;
            &lt;apply&gt;
              &lt;times/&gt;
              &lt;ci&gt; mwddb6a8f0_b347_4faa_9536_294915a489ea &lt;/ci&gt;
              &lt;ci&gt; mw38ea07af_24e1_43bd_b07b_ec2a1371f4d4 &lt;/ci&gt;
              &lt;ci&gt; mw1e889781_12c4_4172_91b6_546e3a00bfb4 &lt;/ci&gt;
              &lt;ci&gt; mw8607693c_2da9_4dd0_9167_ab2793ec2f3a &lt;/ci&gt;
            &lt;/apply&gt;
          &lt;/math&gt;
        &lt;/kineticLaw&gt;
      &lt;/reaction&gt;
      <reaction id="mwd23188d3_14cb_4024_bdec_e87125e778d9"
reversible="false" fast="false">
        &lt;notes&gt;
          &lt;body xmlns="http://www.w3.org/1999/xhtml"&gt;aT0 cell
proliferation&lt;/body&gt;
        &lt;/notes&gt;
        &lt;listOfReactants&gt;
          <speciesReference
species="mw26ca4282_20b3_4f9b_8ce4_58824cec85fc" stoichiometry="1"/>
        &lt;/listOfReactants&gt;
        &lt;kineticLaw&gt;
          &lt;math xmlns="http://www.w3.org/1998/Math/MathML"&gt;
            &lt;apply&gt;
              &lt;times/&gt;
              &lt;apply&gt;
                &lt;divide/&gt;
                &lt;ci&gt; mw1f6f8208_3f72_4671_8286_8e080906848d &lt;/ci&gt;
                &lt;ci&gt; mw41e31e9a_6f80_41a3_a437_bfaa6e8baed9 &lt;/ci&gt;
              &lt;/apply&gt;
              &lt;ci&gt; mw26ca4282_20b3_4f9b_8ce4_58824cec85fc &lt;/ci&gt;
            &lt;/apply&gt;
          &lt;/math&gt;
        &lt;/kineticLaw&gt;
```

```
      </reaction>
      <reaction id="mw16744246_679c_4386_b9b0_05ab8b2d632a"
reversible="false" fast="false">
        <notes>
          <body xmlns="http://www.w3.org/1999/xhtml">aT0 cell
proliferation</body>
        </notes>
        <listOfProducts>
          <speciesReference
species="mwd3fc3e45_040b_42b7_b9c1_0f60a875237c" stoichiometry="1"/>
        </listOfProducts>
        <kineticLaw>
          <math xmlns="http://www.w3.org/1998/Math/MathML">
            <apply>
              <times/>
              <apply>
                <divide/>
                <ci> mw1f6f8208_3f72_4671_8286_8e080906848d </ci>
                <ci> mw41e31e9a_6f80_41a3_a437_bfaa6e8baed9 </ci>
              </apply>
              <cn type="integer"> 2 </cn>
              <apply>
                <exp/>
                <apply>
                  <minus/>
                  <ci> mw41e31e9a_6f80_41a3_a437_bfaa6e8baed9 </ci>
                  <cn type="integer"> 1 </cn>
                </apply>
              </apply>
              <ci> mw26ca4282_20b3_4f9b_8ce4_58824cec85fc </ci>
              <apply>
                <minus/>
                <cn type="integer"> 1 </cn>
                <ci> mw5fab6ae8_fb41_4c3a_818e_ce71aaaac5d9 </ci>
              </apply>
            </apply>
          </math>
        </kineticLaw>
      </reaction>
      <reaction id="mwcf01c3b9_3c0b_4a08_9935_bd42d86eb9df"
reversible="false" fast="false">
        <notes>
          <body xmlns="http://www.w3.org/1999/xhtml">T cell death in
the central compartment</body>
        </notes>
        <listOfReactants>
          <speciesReference
species="mw2f881f74_14f8_45be_ac4a_bb4e1e3ede99" stoichiometry="1"/>
        </listOfReactants>
        <kineticLaw>
          <math xmlns="http://www.w3.org/1998/Math/MathML">
            <apply>
```

BMJ Publishing Group Limited (BMJ) disclaims all liability and responsibility arising from any reliance placed on this supplemental material which has been supplied by the author(s)


```
              <times/>
              <ci> mw33b5d408_7337_4983_b330_cfcb3f4357af </ci>
              <ci> mw2f881f74_14f8_45be_ac4a_bb4e1e3ede99 </ci>
            </apply>
          </math>
        </kineticLaw>
      </reaction>
      <reaction id="mwdb41221f_d539_4b13_8a64_1226e23f6cea"
reversible="false" fast="false">
        <notes>
          <body xmlns="http://www.w3.org/1999/xhtml">T cell death in
the peripheral compartment</body>
        </notes>
        <listOfReactants>
          <speciesReference
species="mw56b94d8b_7702_49a1_a07a_62a53c209830" stoichiometry="1"/>
        </listOfReactants>
        <kineticLaw>
          <math xmlns="http://www.w3.org/1998/Math/MathML">
            <apply>
              <times/>
              <ci> mw33b5d408_7337_4983_b330_cfcb3f4357af </ci>
              <ci> mw56b94d8b_7702_49a1_a07a_62a53c209830 </ci>
            </apply>
          </math>
        </kineticLaw>
      </reaction>
      <reaction id="mw667f46d5_a931_4b54_a343_5cbb50b730a6"
reversible="false" fast="false">
        <notes>
          <body xmlns="http://www.w3.org/1999/xhtml">T cell death in
the tumour compartment</body>
        </notes>
        <listOfReactants>
          <speciesReference
species="mwa5fd10b1_5a9e_41d2_a246_260c6fc1dbf5" stoichiometry="1"/>
        </listOfReactants>
        <listOfProducts>
          <speciesReference
species="mw088a157f_05c9_4257_a674_1b5bb4ee465b" stoichiometry="1"/>
        </listOfProducts>
        <kineticLaw>
          <math xmlns="http://www.w3.org/1998/Math/MathML">
            <apply>
              <times/>
              <ci> mw33b5d408_7337_4983_b330_cfcb3f4357af </ci>
              <ci> mwa5fd10b1_5a9e_41d2_a246_260c6fc1dbf5 </ci>
            </apply>
          </math>
        </kineticLaw>
      </reaction>
```

```
      <reaction id="mw588aa936_f0eb_4e95_a233_2fe0fa2e7abf"
reversible="false" fast="false">
        <notes>
          <body xmlns="http://www.w3.org/1999/xhtml">T cell death in
the lymph node compartment</body>
        </notes>
        <listOfReactants>
          <speciesReference
species="mwd3fc3e45_040b_42b7_b9c1_0f60a875237c" stoichiometry="1"/>
        </listOfReactants>
        <kineticLaw>
          <math xmlns="http://www.w3.org/1998/Math/MathML">
            <apply>
              <times/>
              <ci> mw33b5d408_7337_4983_b330_cfcb3f4357af </ci>
              <ci> mwd3fc3e45_040b_42b7_b9c1_0f60a875237c </ci>
            </apply>
          </math>
        </kineticLaw>
      </reaction>
      <reaction id="mwa2b9b6cd_bfa2_4bec_962b_cfb9ef5bdb9a"
reversible="false" fast="false">
        <notes>
          <body xmlns="http://www.w3.org/1999/xhtml">T cell transport
into the peripheral compartment</body>
        </notes>
        <listOfReactants>
          <speciesReference
species="mw2f881f74_14f8_45be_ac4a_bb4e1e3ede99" stoichiometry="1"/>
        </listOfReactants>
        <listOfProducts>
          <speciesReference
species="mw56b94d8b_7702_49a1_a07a_62a53c209830" stoichiometry="1"/>
        </listOfProducts>
        <kineticLaw>
          <math xmlns="http://www.w3.org/1998/Math/MathML">
            <apply>
              <times/>
              <ci> mwddf444aa_74ff_4ba5_81a2_b30b34dc661e </ci>
              <ci> mw2f881f74_14f8_45be_ac4a_bb4e1e3ede99 </ci>
            </apply>
          </math>
        </kineticLaw>
      </reaction>
      <reaction id="mw025b4abb_2dc4_4950_93cd_35e7d5b344f2"
reversible="false" fast="false">
        <notes>
          <body xmlns="http://www.w3.org/1999/xhtml">T cell transport
out of the peripheral compartment</body>
        </notes>
        <listOfReactants>
```

```
          <speciesReference
species="mw56b94d8b_7702_49a1_a07a_62a53c209830" stoichiometry="1"/>
        </listOfReactants>
        <listOfProducts>
          <speciesReference
species="mw2f881f74_14f8_45be_ac4a_bb4e1e3ede99" stoichiometry="1"/>
        </listOfProducts>
        <kineticLaw>
          <math xmlns="http://www.w3.org/1998/Math/MathML">
            <apply>
              <times/>
              <ci> mw6b8e638c_f28a_4c66_9b9c_c58ded48ccc0 </ci>
              <ci> mw56b94d8b_7702_49a1_a07a_62a53c209830 </ci>
            </apply>
          </math>
        </kineticLaw>
      </reaction>
      <reaction id="mw15cc195c_b8c9_4095_92c9_fb0f77d33d4e"
reversible="false" fast="false">
        <notes>
          <body xmlns="http://www.w3.org/1999/xhtml">T cell transport
into the tumour compartment</body>
        </notes>
        <listOfReactants>
          <speciesReference
species="mw2f881f74_14f8_45be_ac4a_bb4e1e3ede99" stoichiometry="1"/>
        </listOfReactants>
        <listOfProducts>
          <speciesReference
species="mwa5fd10b1_5a9e_41d2_a246_260c6fc1dbf5" stoichiometry="1"/>
        </listOfProducts>
        <kineticLaw>
          <math xmlns="http://www.w3.org/1998/Math/MathML">
            <apply>
              <times/>
              <ci> mw302fac6a_5a44_40e3_a0dc_31785767fc4f </ci>
              <ci> mw175e8610_7578_45f5_80bb_7b61e6e241ab </ci>
              <ci> mw2f881f74_14f8_45be_ac4a_bb4e1e3ede99 </ci>
            </apply>
          </math>
        </kineticLaw>
      </reaction>
      <reaction id="mwc6099b55_1d18_4d89_9639_6cab518dd3d1"
reversible="false" fast="false">
        <notes>
          <body xmlns="http://www.w3.org/1999/xhtml">T cell transport
out of the lymph node compartment</body>
        </notes>
        <listOfReactants>
          <speciesReference
species="mwd3fc3e45_040b_42b7_b9c1_0f60a875237c" stoichiometry="1"/>
        </listOfReactants>
```

```
        <listOfProducts>
          <speciesReference
species="mw2f881f74_14f8_45be_ac4a_bb4e1e3ede99" stoichiometry="1"/>
        </listOfProducts>
        <kineticLaw>
          <math xmlns="http://www.w3.org/1998/Math/MathML">
            <apply>
              <times/>
              <ci> mwbc88286a_79e7_4c26_a3ec_720caf764025 </ci>
              <ci> mwd3fc3e45_040b_42b7_b9c1_0f60a875237c </ci>
            </apply>
          </math>
        </kineticLaw>
      </reaction>
      <reaction id="mw17c09118_be9f_4275_b9a1_4f2cc4922b43"
reversible="false" fast="false">
        <notes>
          <body xmlns="http://www.w3.org/1999/xhtml">IL2 consumption
by Tregs</body>
        </notes>
        <listOfReactants>
          <speciesReference
species="mw479632c6_8f17_47aa_b429_216752f11c51" stoichiometry="1"/>
        </listOfReactants>
        <kineticLaw>
          <math xmlns="http://www.w3.org/1998/Math/MathML">
            <apply>
              <divide/>
              <apply>
                <times/>
                <ci> mw4db95ce7_153f_4c52_b346_bb639b69966e </ci>
                <ci> mwd3fc3e45_040b_42b7_b9c1_0f60a875237c </ci>
                <ci> mw479632c6_8f17_47aa_b429_216752f11c51 </ci>
              </apply>
              <apply>
                <plus/>
                <ci> mw470a2f94_f39a_45d1_bd57_2f506e768b0b </ci>
                <ci> mw479632c6_8f17_47aa_b429_216752f11c51 </ci>
              </apply>
            </apply>
          </math>
        </kineticLaw>
      </reaction>
      <reaction id="mw934a2343_3613_4d9f_a557_822a672b7f30"
reversible="false" fast="false">
        <notes>
          <body xmlns="http://www.w3.org/1999/xhtml">APC
recruitment/death in the tumour</body>
        </notes>
        <listOfProducts>
          <speciesReference
species="mwf53e0b71_eca1_413f_9604_8297474f789f" stoichiometry="1"/>
```

```
        &lt;/listOfProducts&gt;
        &lt;kineticLaw&gt;
          &lt;math xmlns="http://www.w3.org/1998/Math/MathML"&gt;
            &lt;apply&gt;
              &lt;times/&gt;
              &lt;ci&gt; mwd682106f_de26_4639_8b62_e17205f22c2a &lt;/ci&gt;
              &lt;apply&gt;
                &lt;minus/&gt;
                &lt;apply&gt;
                  &lt;times/&gt;
                  &lt;ci&gt; mw02c23a69_3001_4dcc_a56a_92c6ed513d09 &lt;/ci&gt;
                  &lt;ci&gt; mw175e8610_7578_45f5_80bb_7b61e6e241ab &lt;/ci&gt;
                &lt;/apply&gt;
                &lt;ci&gt; mwf53e0b71_eca1_413f_9604_8297474f789f &lt;/ci&gt;
              &lt;/apply&gt;
            &lt;/apply&gt;
          &lt;/math&gt;
        &lt;/kineticLaw&gt;
      &lt;/reaction&gt;
      <reaction id="mw9fe32b4d_8409_421e_80fb_f61c8cffae4f"
reversible="false" fast="false">
        &lt;notes&gt;
          &lt;body xmlns="http://www.w3.org/1999/xhtml"&gt;APC
recruitment/death in LN&lt;/body&gt;
        &lt;/notes&gt;
        &lt;listOfProducts&gt;
          <speciesReference
species="mw0f3e4986_c5c0_4e85_8f07_03bf0ed91f2b" stoichiometry="1"/>
        &lt;/listOfProducts&gt;
        &lt;kineticLaw&gt;
          &lt;math xmlns="http://www.w3.org/1998/Math/MathML"&gt;
            &lt;apply&gt;
              &lt;times/&gt;
              &lt;ci&gt; mwd682106f_de26_4639_8b62_e17205f22c2a &lt;/ci&gt;
              &lt;apply&gt;
                &lt;minus/&gt;
                &lt;apply&gt;
                  &lt;times/&gt;
                  &lt;ci&gt; mwf5121bef_af1c_473c_a722_fa4c65155afb &lt;/ci&gt;
                  &lt;ci&gt; mwb4901521_e24a_4f39_acff_107e3522faa9 &lt;/ci&gt;
                &lt;/apply&gt;
                &lt;ci&gt; mw0f3e4986_c5c0_4e85_8f07_03bf0ed91f2b &lt;/ci&gt;
              &lt;/apply&gt;
            &lt;/apply&gt;
          &lt;/math&gt;
        &lt;/kineticLaw&gt;
      &lt;/reaction&gt;
      <reaction id="mwcade2a82_1c5e_48e8_9223_00f2559195ff"
reversible="false" fast="false">
        &lt;notes&gt;
          &lt;body xmlns="http://www.w3.org/1999/xhtml"&gt;APC maturation in
the tumour&lt;/body&gt;
```

```
        </notes>
        <listOfReactants>
          <speciesReference
species="mwf53e0b71_eca1_413f_9604_8297474f789f" stoichiometry="1"/>
        </listOfReactants>
        <listOfProducts>
          <speciesReference
species="mw40d0c8ab_6e98_45f5_a2d7_4f4167cfc589" stoichiometry="1"/>
        </listOfProducts>
        <kineticLaw>
          <math xmlns="http://www.w3.org/1998/Math/MathML">
            <apply>
              <times/>
              <apply>
                <divide/>
                <apply>
                  <times/>
                  <ci> mw53fa75fa_b009_489f_9a91_2299c40b06e7 </ci>
                  <ci> mw1a0b99a8_2ed5_48b7_9014_a1041ffeebb3 </ci>
                </apply>
                <apply>
                  <plus/>
                  <ci> mw1a0b99a8_2ed5_48b7_9014_a1041ffeebb3 </ci>
                  <ci> mwf7577d46_55a5_459f_86f6_36b2f0c1829b </ci>
                </apply>
              </apply>
              <ci> mwf53e0b71_eca1_413f_9604_8297474f789f </ci>
            </apply>
          </math>
        </kineticLaw>
      </reaction>
      <reaction id="mw9440c076_c6a9_45c4_b6af_ca28e2005293"
reversible="false" fast="false">
        <notes>
          <body xmlns="http://www.w3.org/1999/xhtml">APC migration to
the lymph node</body>
        </notes>
        <listOfReactants>
          <speciesReference
species="mw40d0c8ab_6e98_45f5_a2d7_4f4167cfc589" stoichiometry="1"/>
        </listOfReactants>
        <listOfProducts>
          <speciesReference
species="mw507424c1_49c9_4a06_8493_4e61e9f65e52" stoichiometry="1"/>
        </listOfProducts>
        <kineticLaw>
          <math xmlns="http://www.w3.org/1998/Math/MathML">
            <apply>
              <times/>
              <ci> mwa25696c4_7c1f_493f_9fcf_dfe77a664fda </ci>
              <ci> mw40d0c8ab_6e98_45f5_a2d7_4f4167cfc589 </ci>
            </apply>
```

```
          </math>
        </kineticLaw>
      </reaction>
      <reaction id="mw43c08d34_fa77_45c0_9b4c_98121e24f3d1"
reversible="false" fast="false">
        <notes>
          <body xmlns="http://www.w3.org/1999/xhtml">mAPC death in the
tumour</body>
        </notes>
        <listOfReactants>
          <speciesReference
species="mw40d0c8ab_6e98_45f5_a2d7_4f4167cfc589" stoichiometry="1"/>
        </listOfReactants>
        <kineticLaw>
          <math xmlns="http://www.w3.org/1998/Math/MathML">
            <apply>
              <times/>
              <ci> mwb5663a72_7385_40b3_b101_1a11d4351ffb </ci>
              <ci> mw40d0c8ab_6e98_45f5_a2d7_4f4167cfc589 </ci>
            </apply>
          </math>
        </kineticLaw>
      </reaction>
      <reaction id="mw5db525f2_fa5d_469f_814c_8fd1400f1f23"
reversible="false" fast="false">
        <notes>
          <body xmlns="http://www.w3.org/1999/xhtml">mAPC death in the
lymph node</body>
        </notes>
        <listOfReactants>
          <speciesReference
species="mw507424c1_49c9_4a06_8493_4e61e9f65e52" stoichiometry="1"/>
        </listOfReactants>
        <kineticLaw>
          <math xmlns="http://www.w3.org/1998/Math/MathML">
            <apply>
              <times/>
              <ci> mwb5663a72_7385_40b3_b101_1a11d4351ffb </ci>
              <ci> mw507424c1_49c9_4a06_8493_4e61e9f65e52 </ci>
            </apply>
          </math>
        </kineticLaw>
      </reaction>
      <reaction id="mw933d1b85_3660_4556_8121_eeebc1cde973"
reversible="false" fast="false">
        <notes>
          <body xmlns="http://www.w3.org/1999/xhtml">Baseline cytokine
secretion/degradation</body>
        </notes>
        <listOfProducts>
          <speciesReference
species="mw1a0b99a8_2ed5_48b7_9014_a1041ffeebb3" stoichiometry="1"/>
```

```
        </listOfProducts>
        <kineticLaw>
          <math xmlns="http://www.w3.org/1998/Math/MathML">
            <apply>
              <times/>
              <ci> mw7d571cef_3602_4299_9ec8_f9d570e17881 </ci>
              <apply>
                <minus/>
                <ci> mw5ccd755d_506a_494a_8e09_94b0a330e418 </ci>
                <ci> mw1a0b99a8_2ed5_48b7_9014_a1041ffeebb3 </ci>
              </apply>
              <ci> mw175e8610_7578_45f5_80bb_7b61e6e241ab </ci>
            </apply>
          </math>
        </kineticLaw>
      </reaction>
      <reaction id="mw0dc39380_3116_45b6_92a5_86c67b1648fa"
reversible="false" fast="false">
        <notes>
          <body xmlns="http://www.w3.org/1999/xhtml">Cytokine release
in response to the tumour</body>
        </notes>
        <listOfProducts>
          <speciesReference
species="mw1a0b99a8_2ed5_48b7_9014_a1041ffeebb3" stoichiometry="1"/>
        </listOfProducts>
        <kineticLaw>
          <math xmlns="http://www.w3.org/1998/Math/MathML">
            <apply>
              <times/>
              <ci> mwcc034ae1_f0c4_4edf_b337_d36183873773 </ci>
              <ci> mwcb716e87_b63f_40df_a44f_1002c7d61986 </ci>
            </apply>
          </math>
        </kineticLaw>
      </reaction>
      <reaction id="mw7ef4bd68_973a_4765_a42e_673553cccc2a"
reversible="false" fast="false">
        <notes>
          <body xmlns="http://www.w3.org/1999/xhtml">MHC
translocation</body>
        </notes>
        <listOfReactants>
          <speciesReference
species="mw942caa29_eb06_4495_9672_6335f8011e75" stoichiometry="1"/>
        </listOfReactants>
        <listOfProducts>
          <speciesReference
species="mw832e95af_83ea_46bd_9427_ce8eae60b699" stoichiometry="1"/>
        </listOfProducts>
        <kineticLaw>
          <math xmlns="http://www.w3.org/1998/Math/MathML">
```

```
                <apply>
                  <minus/>
                  <apply>
                    <times/>
                    <ci> mw188d8839_a857_4645_a216_9e090aa6baad </ci>
                    <ci> mw942caa29_eb06_4495_9672_6335f8011e75 </ci>
                    <ci> mwc9c2eea0_d90b_4a89_901f_6f5f9de09f35 </ci>
                  </apply>
                  <apply>
                    <times/>
                    <ci> mw3852f618_204c_448a_b245_9df43b665635 </ci>
                    <ci> mw832e95af_83ea_46bd_9427_ce8eae60b699 </ci>
                    <ci> mwa56ed94a_0a50_45aa_b713_70c4c2665228 </ci>
                  </apply>
                </apply>
              </math>
            </kineticLaw>
          </reaction>
          <reaction id="mw6cfe0870_5c2b_4964_aa18_3331f3a30e03"
reversible="false" fast="false">
            <notes>
              <body xmlns="http://www.w3.org/1999/xhtml">Antigen
deposition from dying cancer cells</body>
            </notes>
            <listOfProducts>
              <speciesReference
species="mwfd9cdc6d_ef96_441b_9ef2_89e3ce96bf71" stoichiometry="1"/>
            </listOfProducts>
            <kineticLaw>
              <math xmlns="http://www.w3.org/1998/Math/MathML">
                <apply>
                  <times/>
                  <ci> mwcdf3bb71_6afa_48e7_b780_2d7ee5f9b5db </ci>
                  <ci> mw62203837_dd40_4baa_8b94_89d3fc6cc634 </ci>
                  <apply>
                    <plus/>
                    <ci> mw92cb2786_b098_48c2_a8dd_18d26e65a0d6 </ci>
                    <ci> mw47b25c11_9847_4587_8215_ed3f3ef66f20 </ci>
                    <apply>
                      <times/>
                      <apply>
                        <divide/>
                        <apply>
                          <times/>
                          <ci> mw4b836c57_546e_445b_b43d_95ce959cc91a
</ci>
                          <ci> mw66344d11_136c_4af3_9f9a_cc5c3035331c
</ci>
                        </apply>
                        <apply>
                          <plus/>
```

```
                      &lt;ci&gt; mw1e7cee5a_6fd5_4375_9ff9_96509d2f0af7
&lt;/ci&gt;
                      &lt;ci&gt; mw0782fa64_c077_445f_9358_fbb0e8cc0a7e
&lt;/ci&gt;
                      &lt;ci&gt; mw5c6752bd_6029_486c_b439_45f7ca1b3111
&lt;/ci&gt;
                    &lt;/apply&gt;
                  &lt;/apply&gt;
                  &lt;apply&gt;
                    &lt;minus/&gt;
                    &lt;cn type="integer"&gt; 1 &lt;/cn&gt;
                    &lt;ci&gt; mwffa3495a_26ce_48b7_bee5_50af90fed75f &lt;/ci&gt;
                  &lt;/apply&gt;
                &lt;/apply&gt;
              &lt;/apply&gt;
              &lt;ci&gt; mw2aafd721_12db_4909_b572_82abf04c578d &lt;/ci&gt;
              &lt;ci&gt; mw175e8610_7578_45f5_80bb_7b61e6e241ab &lt;/ci&gt;
            &lt;/apply&gt;
          &lt;/math&gt;
        &lt;/kineticLaw&gt;
      &lt;/reaction&gt;
      <reaction id="mw1effc7bc_5459_442f_8e24_9afa7b85906f"
reversible="false" fast="false">
        &lt;notes&gt;
          &lt;body xmlns="http://www.w3.org/1999/xhtml"&gt;Free antigen
degradation&lt;/body&gt;
        &lt;/notes&gt;
        &lt;listOfReactants&gt;
          <speciesReference
species="mwfd9cdc6d_ef96_441b_9ef2_89e3ce96bf71" stoichiometry="1"/>
        &lt;/listOfReactants&gt;
        &lt;kineticLaw&gt;
          &lt;math xmlns="http://www.w3.org/1998/Math/MathML"&gt;
            &lt;apply&gt;
              &lt;times/&gt;
              &lt;ci&gt; mwc17972bb_efbb_4c99_ab02_61201c886f8d &lt;/ci&gt;
              &lt;ci&gt; mwfd9cdc6d_ef96_441b_9ef2_89e3ce96bf71 &lt;/ci&gt;
              &lt;ci&gt; mwb4901521_e24a_4f39_acff_107e3522faa9 &lt;/ci&gt;
            &lt;/apply&gt;
          &lt;/math&gt;
        &lt;/kineticLaw&gt;
      &lt;/reaction&gt;
      <reaction id="mw82320584_cbf3_464b_9da0_2c2b8acd6b5c"
reversible="false" fast="false">
        &lt;notes&gt;
          &lt;body xmlns="http://www.w3.org/1999/xhtml"&gt;Antigen uptake by
mature antigen presenting cells&lt;/body&gt;
        &lt;/notes&gt;
        &lt;listOfReactants&gt;
          <speciesReference
species="mwfd9cdc6d_ef96_441b_9ef2_89e3ce96bf71" stoichiometry="1"/>
        &lt;/listOfReactants&gt;
```

```
        <kineticLaw>
          <math xmlns="http://www.w3.org/1998/Math/MathML">
            <apply>
              <times/>
              <ci> mw6b11c414_a69b_4efd_8a07_d1173fa59a87 </ci>
              <ci> mw507424c1_49c9_4a06_8493_4e61e9f65e52 </ci>
              <ci> mwfd9cdc6d_ef96_441b_9ef2_89e3ce96bf71 </ci>
              <ci> mwb4901521_e24a_4f39_acff_107e3522faa9 </ci>
            </apply>
          </math>
        </kineticLaw>
      </reaction>
      <reaction id="mw7d113a5b_429e_40ef_a427_3ff2f3baae28"
reversible="false" fast="false">
        <notes>
          <body xmlns="http://www.w3.org/1999/xhtml">Antigen uptake by
mature antigen presenting cells</body>
        </notes>
        <listOfProducts>
          <speciesReference
species="mw3cfa9342_80e4_4ad5_a763_147d1db83eef" stoichiometry="1"/>
        </listOfProducts>
        <kineticLaw>
          <math xmlns="http://www.w3.org/1998/Math/MathML">
            <apply>
              <times/>
              <ci> mw6b11c414_a69b_4efd_8a07_d1173fa59a87 </ci>
              <ci> mw5c6752bd_6029_486c_b439_45f7ca1b3111 </ci>
              <ci> mwfd9cdc6d_ef96_441b_9ef2_89e3ce96bf71 </ci>
              <ci> mwf681189d_0fc1_4538_a15f_815606c049a6 </ci>
            </apply>
          </math>
        </kineticLaw>
      </reaction>
      <reaction id="mw218d2a84_9d0c_46d1_acab_a4f6c62a20c0"
reversible="false" fast="false">
        <notes>
          <body xmlns="http://www.w3.org/1999/xhtml">Antigen
degradation in APC endosomes</body>
        </notes>
        <listOfReactants>
          <speciesReference
species="mw3cfa9342_80e4_4ad5_a763_147d1db83eef" stoichiometry="1"/>
        </listOfReactants>
        <listOfProducts>
          <speciesReference
species="mw833b4186_5415_4464_ba45_e286d775edb5" stoichiometry="1"/>
        </listOfProducts>
        <kineticLaw>
          <math xmlns="http://www.w3.org/1998/Math/MathML">
            <apply>
              <times/>
```

```
              <ci> mw3cf8aee1_8eb9_4ee4_93a7_61eb9f20252f </ci>
              <ci> mw3cfa9342_80e4_4ad5_a763_147d1db83eef </ci>
              <ci> mwf681189d_0fc1_4538_a15f_815606c049a6 </ci>
            </apply>
          </math>
        </kineticLaw>
      </reaction>
      <reaction id="mwa0a0f18d_72de_4b7d_bcfe_6a74c23289da"
reversible="false" fast="false">
        <notes>
          <body xmlns="http://www.w3.org/1999/xhtml">Epitope
degradation in APC endosomes</body>
        </notes>
        <listOfReactants>
          <speciesReference
species="mw833b4186_5415_4464_ba45_e286d775edb5" stoichiometry="1"/>
        </listOfReactants>
        <kineticLaw>
          <math xmlns="http://www.w3.org/1998/Math/MathML">
            <apply>
              <times/>
              <ci> mw08e80428_100e_4181_8dff_866054ccd158 </ci>
              <ci> mw833b4186_5415_4464_ba45_e286d775edb5 </ci>
              <ci> mwf681189d_0fc1_4538_a15f_815606c049a6 </ci>
            </apply>
          </math>
        </kineticLaw>
      </reaction>
      <reaction id="mwdebb54ed_e945_42e7_b648_333b65acaabc"
reversible="false" fast="false">
        <notes>
          <body xmlns="http://www.w3.org/1999/xhtml">Antigen-MHC
binding in endosome</body>
        </notes>
        <listOfReactants>
          <speciesReference
species="mw833b4186_5415_4464_ba45_e286d775edb5" stoichiometry="1"/>
          <speciesReference
species="mw942caa29_eb06_4495_9672_6335f8011e75" stoichiometry="1"/>
        </listOfReactants>
        <listOfProducts>
          <speciesReference
species="mwae431b93_56a6_4720_bd61_fb993f5e5c47" stoichiometry="1"/>
        </listOfProducts>
        <kineticLaw>
          <math xmlns="http://www.w3.org/1998/Math/MathML">
            <apply>
              <times/>
              <ci> mw33d69d64_f4f9_4384_9195_c2233607df55 </ci>
              <ci> mw833b4186_5415_4464_ba45_e286d775edb5 </ci>
              <ci> mw942caa29_eb06_4495_9672_6335f8011e75 </ci>
              <ci> mwc9c2eea0_d90b_4a89_901f_6f5f9de09f35 </ci>
```

```
            </apply>
          </math>
        </kineticLaw>
      </reaction>
      <reaction id="mw135d4987_359c_4250_a7d7_92cc9f028105"
reversible="false" fast="false">
        <notes>
          <body xmlns="http://www.w3.org/1999/xhtml">Antigen-MHC
unbinding in endosome</body>
        </notes>
        <listOfReactants>
          <speciesReference
species="mwae431b93_56a6_4720_bd61_fb993f5e5c47" stoichiometry="1"/>
        </listOfReactants>
        <listOfProducts>
          <speciesReference
species="mw833b4186_5415_4464_ba45_e286d775edb5" stoichiometry="1"/>
          <speciesReference
species="mw942caa29_eb06_4495_9672_6335f8011e75" stoichiometry="1"/>
        </listOfProducts>
        <kineticLaw>
          <math xmlns="http://www.w3.org/1998/Math/MathML">
            <apply>
              <times/>
              <ci> mw8cb23925_0616_4ba0_b05f_b7c310055464 </ci>
              <ci> mw33d69d64_f4f9_4384_9195_c2233607df55 </ci>
              <ci> mwae431b93_56a6_4720_bd61_fb993f5e5c47 </ci>
              <ci> mwc9c2eea0_d90b_4a89_901f_6f5f9de09f35 </ci>
            </apply>
          </math>
        </kineticLaw>
      </reaction>
      <reaction id="mw4f4f6120_919e_4b2c_904f_399150fd9a02"
reversible="false" fast="false">
        <notes>
          <body xmlns="http://www.w3.org/1999/xhtml">Antigen-MHC
unbinding on APC surface</body>
        </notes>
        <listOfReactants>
          <speciesReference
species="mwd1db5e41_695c_4769_b3d8_4151bf3a44d0" stoichiometry="1"/>
        </listOfReactants>
        <listOfProducts>
          <speciesReference
species="mw832e95af_83ea_46bd_9427_ce8eae60b699" stoichiometry="1"/>
        </listOfProducts>
        <kineticLaw>
          <math xmlns="http://www.w3.org/1998/Math/MathML">
            <apply>
              <times/>
              <ci> mw8cb23925_0616_4ba0_b05f_b7c310055464 </ci>
              <ci> mw33d69d64_f4f9_4384_9195_c2233607df55 </ci>
```

```
            <ci> mwd1db5e41_695c_4769_b3d8_4151bf3a44d0 </ci>
            <ci> mwa56ed94a_0a50_45aa_b713_70c4c2665228 </ci>
          </apply>
        </math>
      </kineticLaw>
    </reaction>
    <reaction id="mwd973a300_58e9_4414_9589_4526581a2984"
reversible="false" fast="false">
      <notes>
        <body xmlns="http://www.w3.org/1999/xhtml">Antigen-MHC
translocation</body>
      </notes>
      <listOfReactants>
        <speciesReference
species="mwae431b93_56a6_4720_bd61_fb993f5e5c47" stoichiometry="1"/>
      </listOfReactants>
      <listOfProducts>
        <speciesReference
species="mwd1db5e41_695c_4769_b3d8_4151bf3a44d0" stoichiometry="1"/>
      </listOfProducts>
      <kineticLaw>
        <math xmlns="http://www.w3.org/1998/Math/MathML">
          <apply>
            <times/>
            <ci> mw188d8839_a857_4645_a216_9e090aa6baad </ci>
            <ci> mwae431b93_56a6_4720_bd61_fb993f5e5c47 </ci>
            <ci> mwc9c2eea0_d90b_4a89_901f_6f5f9de09f35 </ci>
          </apply>
        </math>
      </kineticLaw>
    </reaction>
    <reaction id="mw4e4bb097_f332_4149_996f_c081e63acf08"
reversible="false" fast="false">
      <notes>
        <body xmlns="http://www.w3.org/1999/xhtml">Antigen
deposition from dying cancer cells</body>
      </notes>
      <listOfProducts>
        <speciesReference
species="mw38def0d7_03a6_42e4_898f_2b834be9e4a0" stoichiometry="1"/>
      </listOfProducts>
      <kineticLaw>
        <math xmlns="http://www.w3.org/1998/Math/MathML">
          <apply>
            <times/>
            <ci> mw98c1f539_f992_42f9_b09c_d98aa315dd63 </ci>
            <ci> mw09a78ea1_f99a_49fb_8f57_f79df3425689 </ci>
            <apply>
              <plus/>
              <ci> mw92cb2786_b098_48c2_a8dd_18d26e65a0d6 </ci>
              <ci> mw47b25c11_9847_4587_8215_ed3f3ef66f20 </ci>
              <apply>
```

```
                    <times/>
                    <apply>
                      <divide/>
                      <apply>
                        <times/>
                        <ci> mw4b836c57_546e_445b_b43d_95ce959cc91a
</ci>
                        <ci> mw66344d11_136c_4af3_9f9a_cc5c3035331c
</ci>
                      </apply>
                      <apply>
                        <plus/>
                        <ci> mw1e7cee5a_6fd5_4375_9ff9_96509d2f0af7
</ci>
                        <ci> mw0782fa64_c077_445f_9358_fbb0e8cc0a7e
</ci>
                        <ci> mw5c6752bd_6029_486c_b439_45f7ca1b3111
</ci>
                      </apply>
                    </apply>
                    <apply>
                      <minus/>
                      <cn type="integer"> 1 </cn>
                      <ci> mwffa3495a_26ce_48b7_bee5_50af90fed75f </ci>
                    </apply>
                  </apply>
                </apply>
                <ci> mw2aafd721_12db_4909_b572_82abf04c578d </ci>
                <ci> mw175e8610_7578_45f5_80bb_7b61e6e241ab </ci>
              </apply>
            </math>
          </kineticLaw>
        </reaction>
        <reaction id="mw9d63393a_44c0_4b44_ac21_77dbf48f8152"
reversible="false" fast="false">
          <notes>
            <body xmlns="http://www.w3.org/1999/xhtml">Free antigen
degradation</body>
          </notes>
          <listOfReactants>
            <speciesReference
species="mw38def0d7_03a6_42e4_898f_2b834be9e4a0" stoichiometry="1"/>
          </listOfReactants>
          <kineticLaw>
            <math xmlns="http://www.w3.org/1998/Math/MathML">
              <apply>
                <times/>
                <ci> mw853e9708_350e_4ea7_8907_ea58cfa95380 </ci>
                <ci> mw38def0d7_03a6_42e4_898f_2b834be9e4a0 </ci>
                <ci> mwb4901521_e24a_4f39_acff_107e3522faa9 </ci>
              </apply>
            </math>
```

```
        </kineticLaw>
      </reaction>
      <reaction id="mw26b92976_616f_4d6f_811c_2760a6213a92"
reversible="false" fast="false">
        <notes>
          <body xmlns="http://www.w3.org/1999/xhtml">Antigen uptake by
mature antigen presenting cells</body>
        </notes>
        <listOfReactants>
          <speciesReference
species="mw38def0d7_03a6_42e4_898f_2b834be9e4a0" stoichiometry="1"/>
        </listOfReactants>
        <kineticLaw>
          <math xmlns="http://www.w3.org/1998/Math/MathML">
            <apply>
              <times/>
              <ci> mw96f9fe99_e334_402d_b28a_00dbdf7c575f </ci>
              <ci> mw507424c1_49c9_4a06_8493_4e61e9f65e52 </ci>
              <ci> mw38def0d7_03a6_42e4_898f_2b834be9e4a0 </ci>
              <ci> mwb4901521_e24a_4f39_acff_107e3522faa9 </ci>
            </apply>
          </math>
        </kineticLaw>
      </reaction>
      <reaction id="mw26e424ca_705c_4a72_a257_9b92a005e0c7"
reversible="false" fast="false">
        <notes>
          <body xmlns="http://www.w3.org/1999/xhtml">Antigen uptake by
mature antigen presenting cells</body>
        </notes>
        <listOfProducts>
          <speciesReference
species="mw53b8f0a1_9dae_4ac6_be5b_0b3e78208e34" stoichiometry="1"/>
        </listOfProducts>
        <kineticLaw>
          <math xmlns="http://www.w3.org/1998/Math/MathML">
            <apply>
              <times/>
              <ci> mw96f9fe99_e334_402d_b28a_00dbdf7c575f </ci>
              <ci> mw5c6752bd_6029_486c_b439_45f7ca1b3111 </ci>
              <ci> mw38def0d7_03a6_42e4_898f_2b834be9e4a0 </ci>
              <ci> mwf681189d_0fc1_4538_a15f_815606c049a6 </ci>
            </apply>
          </math>
        </kineticLaw>
      </reaction>
      <reaction id="mw957c386a_fadc_4f81_a267_fbb17e5be995"
reversible="false" fast="false">
        <notes>
          <body xmlns="http://www.w3.org/1999/xhtml">Antigen
degradation in APC endosomes</body>
        </notes>
```

BMJ Publishing Group Limited (BMJ) disclaims all liability and responsibility arising from any reliance placed on this supplemental material which has been supplied by the author(s)


```
        <listOfReactants>
          <speciesReference
species="mw53b8f0a1_9dae_4ac6_be5b_0b3e78208e34" stoichiometry="1"/>
        </listOfReactants>
        <listOfProducts>
          <speciesReference
species="mwdd1d892a_1451_4309_955b_e886b7583ba5" stoichiometry="1"/>
        </listOfProducts>
        <kineticLaw>
          <math xmlns="http://www.w3.org/1998/Math/MathML">
            <apply>
              <times/>
              <ci> mwf862cc85_c33f_4654_a561_ae76fa81bb97 </ci>
              <ci> mw53b8f0a1_9dae_4ac6_be5b_0b3e78208e34 </ci>
              <ci> mwf681189d_0fc1_4538_a15f_815606c049a6 </ci>
            </apply>
          </math>
        </kineticLaw>
      </reaction>
      <reaction id="mw69d07d0d_5a81_472b_95b3_e5e1294d5779"
reversible="false" fast="false">
        <notes>
          <body xmlns="http://www.w3.org/1999/xhtml">Epitope
degradation in APC endosomes</body>
        </notes>
        <listOfReactants>
          <speciesReference
species="mwdd1d892a_1451_4309_955b_e886b7583ba5" stoichiometry="1"/>
        </listOfReactants>
        <kineticLaw>
          <math xmlns="http://www.w3.org/1998/Math/MathML">
            <apply>
              <times/>
              <ci> mwfc10a527_910a_4ef1_8947_e37acf0cf148 </ci>
              <ci> mwdd1d892a_1451_4309_955b_e886b7583ba5 </ci>
              <ci> mwf681189d_0fc1_4538_a15f_815606c049a6 </ci>
            </apply>
          </math>
        </kineticLaw>
      </reaction>
      <reaction id="mw06ba488d_27e5_4be7_b0d3_d6b16db0eb96"
reversible="false" fast="false">
        <notes>
          <body xmlns="http://www.w3.org/1999/xhtml">Antigen-MHC
binding in endosome</body>
        </notes>
        <listOfReactants>
          <speciesReference
species="mwdd1d892a_1451_4309_955b_e886b7583ba5" stoichiometry="1"/>
          <speciesReference
species="mw942caa29_eb06_4495_9672_6335f8011e75" stoichiometry="1"/>
        </listOfReactants>
```

```
        <listOfProducts>
          <speciesReference
species="mw0e63cb09_149e_4a8e_b596_9e21ce9234cd" stoichiometry="1"/>
        </listOfProducts>
        <kineticLaw>
          <math xmlns="http://www.w3.org/1998/Math/MathML">
            <apply>
              <times/>
              <ci> mwddabbddc_09d9_4688_97e3_0ec72d15abaf </ci>
              <ci> mwdd1d892a_1451_4309_955b_e886b7583ba5 </ci>
              <ci> mw942caa29_eb06_4495_9672_6335f8011e75 </ci>
              <ci> mwc9c2eea0_d90b_4a89_901f_6f5f9de09f35 </ci>
            </apply>
          </math>
        </kineticLaw>
      </reaction>
      <reaction id="mwa9581c1b_f8b1_4047_a782_63f6867a7d2c"
reversible="false" fast="false">
        <notes>
          <body xmlns="http://www.w3.org/1999/xhtml">Antigen-MHC
unbinding in endosome</body>
        </notes>
        <listOfReactants>
          <speciesReference
species="mw0e63cb09_149e_4a8e_b596_9e21ce9234cd" stoichiometry="1"/>
        </listOfReactants>
        <listOfProducts>
          <speciesReference
species="mwdd1d892a_1451_4309_955b_e886b7583ba5" stoichiometry="1"/>
          <speciesReference
species="mw942caa29_eb06_4495_9672_6335f8011e75" stoichiometry="1"/>
        </listOfProducts>
        <kineticLaw>
          <math xmlns="http://www.w3.org/1998/Math/MathML">
            <apply>
              <times/>
              <ci> mw0c9670f6_ecd6_4631_90cc_30463073a545 </ci>
              <ci> mwddabbddc_09d9_4688_97e3_0ec72d15abaf </ci>
              <ci> mw0e63cb09_149e_4a8e_b596_9e21ce9234cd </ci>
              <ci> mwc9c2eea0_d90b_4a89_901f_6f5f9de09f35 </ci>
            </apply>
          </math>
        </kineticLaw>
      </reaction>
      <reaction id="mw4e06014d_16b7_4c47_87eb_ed786b18181a"
reversible="false" fast="false">
        <notes>
          <body xmlns="http://www.w3.org/1999/xhtml">Antigen-MHC
unbinding on APC surface</body>
        </notes>
        <listOfReactants>
```

```
          <speciesReference
species="mwf7546543_1576_4ff3_a1d2_09f9d9d8f508" stoichiometry="1"/>
        </listOfReactants>
        <listOfProducts>
          <speciesReference
species="mw832e95af_83ea_46bd_9427_ce8eae60b699" stoichiometry="1"/>
        </listOfProducts>
        <kineticLaw>
          <math xmlns="http://www.w3.org/1998/Math/MathML">
            <apply>
              <times/>
              <ci> mw0c9670f6_ecd6_4631_90cc_30463073a545 </ci>
              <ci> mwddabbddc_09d9_4688_97e3_0ec72d15abaf </ci>
              <ci> mwf7546543_1576_4ff3_a1d2_09f9d9d8f508 </ci>
              <ci> mwa56ed94a_0a50_45aa_b713_70c4c2665228 </ci>
            </apply>
          </math>
        </kineticLaw>
      </reaction>
      <reaction id="mwe3d43daf_8e90_431e_86db_52343306cfe4"
reversible="false" fast="false">
        <notes>
          <body xmlns="http://www.w3.org/1999/xhtml">Antigen-MHC
translocation</body>
        </notes>
        <listOfReactants>
          <speciesReference
species="mw0e63cb09_149e_4a8e_b596_9e21ce9234cd" stoichiometry="1"/>
        </listOfReactants>
        <listOfProducts>
          <speciesReference
species="mwf7546543_1576_4ff3_a1d2_09f9d9d8f508" stoichiometry="1"/>
        </listOfProducts>
        <kineticLaw>
          <math xmlns="http://www.w3.org/1998/Math/MathML">
            <apply>
              <times/>
              <ci> mw188d8839_a857_4645_a216_9e090aa6baad </ci>
              <ci> mw0e63cb09_149e_4a8e_b596_9e21ce9234cd </ci>
              <ci> mwc9c2eea0_d90b_4a89_901f_6f5f9de09f35 </ci>
            </apply>
          </math>
        </kineticLaw>
      </reaction>
      <reaction id="mw5ec14e41_bfdb_432e_805b_b8a8d332c306"
reversible="true" fast="false">
        <notes>
          <body xmlns="http://www.w3.org/1999/xhtml">atezolizumab
diffusive transport to peripheral compartment</body>
        </notes>
        <listOfReactants>
```

```
          <speciesReference
species="mw700cf3ed_1735_4bbc_870e_8572cc73d50a" stoichiometry="1"/>
        &lt;/listOfReactants&gt;
        &lt;listOfProducts&gt;
          <speciesReference
species="mw5581393f_3eaf_48a1_9e8b_e42b2557f0d9" stoichiometry="1"/>
        &lt;/listOfProducts&gt;
        &lt;kineticLaw&gt;
          &lt;math xmlns="http://www.w3.org/1998/Math/MathML"&gt;
            &lt;apply&gt;
              &lt;times/&gt;
              &lt;ci&gt; mw01026970_e68e_4663_a8f4_3c89fbb89a28 &lt;/ci&gt;
              &lt;apply&gt;
                &lt;minus/&gt;
                &lt;apply&gt;
                  &lt;divide/&gt;
                  &lt;ci&gt; mw700cf3ed_1735_4bbc_870e_8572cc73d50a &lt;/ci&gt;
                  &lt;ci&gt; mwa5c3d9de_dbc4_4f10_a373_24389f61938a &lt;/ci&gt;
                &lt;/apply&gt;
                &lt;apply&gt;
                  &lt;divide/&gt;
                  &lt;ci&gt; mw5581393f_3eaf_48a1_9e8b_e42b2557f0d9 &lt;/ci&gt;
                  &lt;ci&gt; mw81e3f6ab_7c90_45d2_828d_5d9e8d54f20c &lt;/ci&gt;
                &lt;/apply&gt;
              &lt;/apply&gt;
            &lt;/apply&gt;
          &lt;/math&gt;
        &lt;/kineticLaw&gt;
      &lt;/reaction&gt;
      <reaction id="mw671efa21_f20b_4a85_b1eb_d2fe9a33714c"
reversible="true" fast="false">
        &lt;notes&gt;
          &lt;body xmlns="http://www.w3.org/1999/xhtml"&gt;atezolizumab
diffusive transport to tumor compartment&lt;/body&gt;
        &lt;/notes&gt;
        &lt;listOfReactants&gt;
          <speciesReference
species="mw700cf3ed_1735_4bbc_870e_8572cc73d50a" stoichiometry="1"/>
        &lt;/listOfReactants&gt;
        &lt;listOfProducts&gt;
          <speciesReference
species="mw59118247_974d_4558_88d8_f2e55f289d1a" stoichiometry="1"/>
        &lt;/listOfProducts&gt;
        &lt;kineticLaw&gt;
          &lt;math xmlns="http://www.w3.org/1998/Math/MathML"&gt;
            &lt;apply&gt;
              &lt;times/&gt;
              &lt;ci&gt; mwb04099fe_184d_408f_b64a_5211b48295fa &lt;/ci&gt;
              &lt;apply&gt;
                &lt;minus/&gt;
                &lt;apply&gt;
                  &lt;divide/&gt;
```

```
                  <ci> mw700cf3ed_1735_4bbc_870e_8572cc73d50a </ci>
                  <ci> mwa5c3d9de_dbc4_4f10_a373_24389f61938a </ci>
                </apply>
                <apply>
                  <divide/>
                  <ci> mw59118247_974d_4558_88d8_f2e55f289d1a </ci>
                  <ci> mwe8121890_8596_4287_b3ee_50b05edf5540 </ci>
                </apply>
              </apply>
              <ci> mw8cfbefd5_8b10_411d_9d84_300692817ff2 </ci>
            </apply>
          </math>
        </kineticLaw>
      </reaction>
      <reaction id="mwd2c3df72_e55b_439f_85d5_ad72a5baecd4"
reversible="true" fast="false">
        <notes>
          <body xmlns="http://www.w3.org/1999/xhtml">atezolizumab
diffusive transport to lymph node compartment</body>
        </notes>
        <listOfReactants>
          <speciesReference
species="mw700cf3ed_1735_4bbc_870e_8572cc73d50a" stoichiometry="1"/>
        </listOfReactants>
        <listOfProducts>
          <speciesReference
species="mw2a165e3b_dfa5_4d73_9af6_2fd6099e47e3" stoichiometry="1"/>
        </listOfProducts>
        <kineticLaw>
          <math xmlns="http://www.w3.org/1998/Math/MathML">
            <apply>
              <times/>
              <ci> mwbfae92a6_4484_4c8d_954b_821e213acb8f </ci>
              <apply>
                <minus/>
                <apply>
                  <divide/>
                  <ci> mw700cf3ed_1735_4bbc_870e_8572cc73d50a </ci>
                  <ci> mwa5c3d9de_dbc4_4f10_a373_24389f61938a </ci>
                </apply>
                <apply>
                  <divide/>
                  <ci> mw2a165e3b_dfa5_4d73_9af6_2fd6099e47e3 </ci>
                  <ci> mw3901f164_3d95_4e77_ad0b_2d4452a9d976 </ci>
                </apply>
              </apply>
            </apply>
          </math>
        </kineticLaw>
      </reaction>
      <reaction id="mwe23bf6c6_ff0d_4488_b907_1d68bfa9274e"
reversible="false" fast="false">
```

```
        <notes>
          <body xmlns="http://www.w3.org/1999/xhtml">atezolizumab
convective transport from tumor to lymph node</body>
        </notes>
        <listOfReactants>
          <speciesReference
species="mw59118247_974d_4558_88d8_f2e55f289d1a" stoichiometry="1"/>
        </listOfReactants>
        <listOfProducts>
          <speciesReference
species="mw2a165e3b_dfa5_4d73_9af6_2fd6099e47e3" stoichiometry="1"/>
        </listOfProducts>
        <kineticLaw>
          <math xmlns="http://www.w3.org/1998/Math/MathML">
            <apply>
              <times/>
              <apply>
                <divide/>
                <apply>
                  <times/>
                  <ci> mw4478efbf_cc05_4bfb_a0f1_51d7236455dd </ci>
                  <ci> mw59118247_974d_4558_88d8_f2e55f289d1a </ci>
                </apply>
                <ci> mwe8121890_8596_4287_b3ee_50b05edf5540 </ci>
              </apply>
              <ci> mw175e8610_7578_45f5_80bb_7b61e6e241ab </ci>
            </apply>
          </math>
        </kineticLaw>
      </reaction>
      <reaction id="mw1c1c77a7_6056_4ecd_a0cd_88182b7babc3"
reversible="false" fast="false">
        <notes>
          <body xmlns="http://www.w3.org/1999/xhtml">atezolizumab
convective transport from lymph node to central</body>
        </notes>
        <listOfReactants>
          <speciesReference
species="mw2a165e3b_dfa5_4d73_9af6_2fd6099e47e3" stoichiometry="1"/>
        </listOfReactants>
        <listOfProducts>
          <speciesReference
species="mw700cf3ed_1735_4bbc_870e_8572cc73d50a" stoichiometry="1"/>
        </listOfProducts>
        <kineticLaw>
          <math xmlns="http://www.w3.org/1998/Math/MathML">
            <apply>
              <times/>
              <apply>
                <divide/>
                <apply>
                  <times/>
```

```
                <ci> mw4478efbf_cc05_4bfb_a0f1_51d7236455dd </ci>
                <ci> mw2a165e3b_dfa5_4d73_9af6_2fd6099e47e3 </ci>
              </apply>
              <ci> mw3901f164_3d95_4e77_ad0b_2d4452a9d976 </ci>
            </apply>
            <ci> mwb4901521_e24a_4f39_acff_107e3522faa9 </ci>
          </apply>
        </math>
      </kineticLaw>
    </reaction>
    <reaction id="mw7a629d37_ef4b_464f_8a70_11aaeb8f1f2f"
reversible="false" fast="false">
      <notes>
        <body xmlns="http://www.w3.org/1999/xhtml">Drug clearance
from central compartment</body>
      </notes>
      <listOfReactants>
        <speciesReference
species="mw700cf3ed_1735_4bbc_870e_8572cc73d50a" stoichiometry="1"/>
      </listOfReactants>
      <kineticLaw>
        <math xmlns="http://www.w3.org/1998/Math/MathML">
          <apply>
            <times/>
            <ci> mwb815e7ef_6822_4c31_ad87_3283f2e76ec4 </ci>
            <ci> mw700cf3ed_1735_4bbc_870e_8572cc73d50a </ci>
            <ci> mw8cfbefd5_8b10_411d_9d84_300692817ff2 </ci>
          </apply>
        </math>
      </kineticLaw>
    </reaction>
    <reaction id="mw3f2e91b0_19df_49d2_b15c_2f99e413c8d3"
reversible="true" fast="false">
      <notes>
        <body xmlns="http://www.w3.org/1999/xhtml">binding and
unbinding of PD1 PDL1 in synapse</body>
      </notes>
      <listOfReactants>
        <speciesReference
species="mw895d97df_cb0d_44c8_9785_11b1d6282d01" stoichiometry="1"/>
        <speciesReference
species="mw48d27efc_8ace_4319_b906_4465d77a46fa" stoichiometry="1"/>
      </listOfReactants>
      <listOfProducts>
        <speciesReference
species="mw538e3c56_061a_417d_9d7f_128d0cd23afa" stoichiometry="1"/>
      </listOfProducts>
      <kineticLaw>
        <math xmlns="http://www.w3.org/1998/Math/MathML">
          <apply>
            <times/>
            <apply>
```

```
                <minus/>
                <apply>
                  <times/>
                  <ci> mwe8d5d721_005f_49f7_ada6_e82cd8ca00c6 </ci>
                  <ci> mw895d97df_cb0d_44c8_9785_11b1d6282d01 </ci>
                  <ci> mw48d27efc_8ace_4319_b906_4465d77a46fa </ci>
                </apply>
                <apply>
                  <times/>
                  <ci> mw75aefaf3_1856_43cf_8511_2fa3e0451e0b </ci>
                  <ci> mw538e3c56_061a_417d_9d7f_128d0cd23afa </ci>
                </apply>
              </apply>
              <ci> mw9a8fe99b_34f3_48b8_9432_7de459c65052 </ci>
            </apply>
          </math>
        </kineticLaw>
      </reaction>
      <reaction id="mw7d979b44_ca87_4804_be4c_b23c05349f8e"
reversible="true" fast="false">
        <notes>
          <body xmlns="http://www.w3.org/1999/xhtml">binding and
unbinding of PD1 PDL2 in synapse</body>
        </notes>
        <listOfReactants>
          <speciesReference
species="mw895d97df_cb0d_44c8_9785_11b1d6282d01" stoichiometry="1"/>
          <speciesReference
species="mw46e10e70_4f89_41d2_98d3_04c055feaf2d" stoichiometry="1"/>
        </listOfReactants>
        <listOfProducts>
          <speciesReference
species="mw80d2fce8_7bcd_47a0_8857_df9df2037ea4" stoichiometry="1"/>
        </listOfProducts>
        <kineticLaw>
          <math xmlns="http://www.w3.org/1998/Math/MathML">
            <apply>
              <times/>
              <apply>
                <minus/>
                <apply>
                  <times/>
                  <ci> mw4cfa47a0_69d4_4eec_aa6c_e4470eeeb266 </ci>
                  <ci> mw895d97df_cb0d_44c8_9785_11b1d6282d01 </ci>
                  <ci> mw46e10e70_4f89_41d2_98d3_04c055feaf2d </ci>
                </apply>
                <apply>
                  <times/>
                  <ci> mw93bf4558_6b97_4f12_bc08_c258a1086db9 </ci>
                  <ci> mw80d2fce8_7bcd_47a0_8857_df9df2037ea4 </ci>
                </apply>
              </apply>
```

```
              <ci> mw9a8fe99b_34f3_48b8_9432_7de459c65052 </ci>
            </apply>
          </math>
        </kineticLaw>
      </reaction>
      <reaction id="mwe584413e_12a1_4f88_9e11_05d55538dd3c"
reversible="true" fast="false">
        <notes>
          <body xmlns="http://www.w3.org/1999/xhtml">binding and
unbinding of PD1 to anti-PD1 on T1 surface in synapse</body>
        </notes>
        <listOfReactants>
          <speciesReference
species="mw895d97df_cb0d_44c8_9785_11b1d6282d01" stoichiometry="1"/>
        </listOfReactants>
        <listOfProducts>
          <speciesReference
species="mw0aa710e6_b780_4cb3_a8dc_464b70d319d9" stoichiometry="1"/>
        </listOfProducts>
        <kineticLaw>
          <math xmlns="http://www.w3.org/1998/Math/MathML">
            <apply>
              <times/>
              <apply>
                <minus/>
                <apply>
                  <times/>
                  <cn type="integer"> 2 </cn>
                  <ci> mw8db9064f_6e42_49c7_8a0b_a47da71a3758 </ci>
                  <apply>
                    <divide/>
                    <apply>
                      <times/>
                      <ci> mw895d97df_cb0d_44c8_9785_11b1d6282d01
</ci>
                      <ci> mw75ebeaf4_1dd4_4b61_bdf7_0611af968ca8
</ci>
                    </apply>
                    <cn type="integer"> 1 </cn>
                  </apply>
                </apply>
                <apply>
                  <times/>
                  <ci> mw991ab93b_f5ef_47e4_8661_9588c62249e4 </ci>
                  <ci> mw0aa710e6_b780_4cb3_a8dc_464b70d319d9 </ci>
                </apply>
              </apply>
              <ci> mw9a8fe99b_34f3_48b8_9432_7de459c65052 </ci>
            </apply>
          </math>
        </kineticLaw>
      </reaction>
```

```
      <reaction id="mw66a8f1c1_2417_47fb_8290_43e0c63dba81"
reversible="true" fast="false">
        <notes>
          <body xmlns="http://www.w3.org/1999/xhtml">binding and
unbinding of PD1 to PD1-Nivo on T1 surface in synapse</body>
        </notes>
        <listOfReactants>
          <speciesReference
species="mw0aa710e6_b780_4cb3_a8dc_464b70d319d9" stoichiometry="1"/>
          <speciesReference
species="mw895d97df_cb0d_44c8_9785_11b1d6282d01" stoichiometry="1"/>
        </listOfReactants>
        <listOfProducts>
          <speciesReference
species="mw0e147b6c_09cf_484f_9819_87470663749d" stoichiometry="1"/>
        </listOfProducts>
        <kineticLaw>
          <math xmlns="http://www.w3.org/1998/Math/MathML">
            <apply>
              <times/>
              <apply>
                <minus/>
                <apply>
                  <times/>
                  <ci> mw614c8d32_2578_4c5c_ba98_2300e829174f </ci>
                  <ci> mw8db9064f_6e42_49c7_8a0b_a47da71a3758 </ci>
                  <ci> mw895d97df_cb0d_44c8_9785_11b1d6282d01 </ci>
                  <ci> mw0aa710e6_b780_4cb3_a8dc_464b70d319d9 </ci>
                </apply>
                <apply>
                  <times/>
                  <cn type="integer"> 2 </cn>
                  <ci> mw991ab93b_f5ef_47e4_8661_9588c62249e4 </ci>
                  <ci> mw0e147b6c_09cf_484f_9819_87470663749d </ci>
                </apply>
              </apply>
              <ci> mw9a8fe99b_34f3_48b8_9432_7de459c65052 </ci>
            </apply>
          </math>
        </kineticLaw>
      </reaction>
      <reaction id="mw3ba0cf78_9d83_4f4f_be71_84e26f3ae7b0"
reversible="true" fast="false">
        <notes>
          <body xmlns="http://www.w3.org/1999/xhtml">binding and
unbinding of PDL1 to anit-PDL1 on C1 surface in synapse</body>
        </notes>
        <listOfReactants>
          <speciesReference
species="mw48d27efc_8ace_4319_b906_4465d77a46fa" stoichiometry="1"/>
        </listOfReactants>
        <listOfProducts>
```

```
          <speciesReference
species="mw9c13852d_8802_4ef2_9d33_f72a5a747dea" stoichiometry="1"/>
        </listOfProducts>
        <kineticLaw>
          <math xmlns="http://www.w3.org/1998/Math/MathML">
            <apply>
              <times/>
              <apply>
                <minus/>
                <apply>
                  <times/>
                  <cn type="integer"> 2 </cn>
                  <ci> mwe0f00ed8_dcf4_4859_9755_bb24cd7f35c9 </ci>
                  <apply>
                    <divide/>
                    <apply>
                      <times/>
                      <ci> mw48d27efc_8ace_4319_b906_4465d77a46fa
</ci>
                      <ci> mw140e5cc4_b897_4719_aa3d_0b30f25bc4dd
</ci>
                    </apply>
                    <ci> mwa5c3d9de_dbc4_4f10_a373_24389f61938a </ci>
                  </apply>
                </apply>
                <apply>
                  <times/>
                  <ci> mwe82cc259_fdc8_4239_a6e1_1f0fea41ca6d </ci>
                  <ci> mw9c13852d_8802_4ef2_9d33_f72a5a747dea </ci>
                </apply>
              </apply>
              <ci> mw9a8fe99b_34f3_48b8_9432_7de459c65052 </ci>
            </apply>
          </math>
        </kineticLaw>
      </reaction>
      <reaction id="mwbc04b488_28a8_408d_920c_094aaf1ae4b2"
reversible="true" fast="false">
        <notes>
          <body xmlns="http://www.w3.org/1999/xhtml">binding and
unbinding of PDL1 to PDL1-anti-PDL1 on C1 surface in synapse</body>
        </notes>
        <listOfReactants>
          <speciesReference
species="mw9c13852d_8802_4ef2_9d33_f72a5a747dea" stoichiometry="1"/>
          <speciesReference
species="mw48d27efc_8ace_4319_b906_4465d77a46fa" stoichiometry="1"/>
        </listOfReactants>
        <listOfProducts>
          <speciesReference
species="mwfc64755e_b1aa_411d_a1f7_8bad9fe66a96" stoichiometry="1"/>
        </listOfProducts>
```

```
        <kineticLaw>
          <math xmlns="http://www.w3.org/1998/Math/MathML">
            <apply>
              <times/>
              <apply>
                <minus/>
                <apply>
                  <times/>
                  <ci> mw3e179651_289c_42d8_bfe9_a4412cee4d9c </ci>
                  <ci> mwe0f00ed8_dcf4_4859_9755_bb24cd7f35c9 </ci>
                  <ci> mw48d27efc_8ace_4319_b906_4465d77a46fa </ci>
                  <ci> mw9c13852d_8802_4ef2_9d33_f72a5a747dea </ci>
                </apply>
                <apply>
                  <times/>
                  <cn type="integer"> 2 </cn>
                  <ci> mwe82cc259_fdc8_4239_a6e1_1f0fea41ca6d </ci>
                  <ci> mwfc64755e_b1aa_411d_a1f7_8bad9fe66a96 </ci>
                </apply>
              </apply>
              <ci> mw9a8fe99b_34f3_48b8_9432_7de459c65052 </ci>
            </apply>
          </math>
        </kineticLaw>
      </reaction>
      <reaction id="mw767d07a4_3df2_4acd_8bb8_e85b020c0ffe"
reversible="true" fast="false">
        <notes>
          <body xmlns="http://www.w3.org/1999/xhtml">binding and
unbinding of PD1 PDL1 in synapse</body>
        </notes>
        <listOfReactants>
          <speciesReference
species="mw4d33db81_6628_421d_8c61_5ef4a4a647ad" stoichiometry="1"/>
          <speciesReference
species="mw70a631d4_9f40_4bbb_bb75_a900f1130935" stoichiometry="1"/>
        </listOfReactants>
        <listOfProducts>
          <speciesReference
species="mwc47a95ad_5c77_4a62_b9b7_cb2b8abe3260" stoichiometry="1"/>
        </listOfProducts>
        <kineticLaw>
          <math xmlns="http://www.w3.org/1998/Math/MathML">
            <apply>
              <times/>
              <apply>
                <minus/>
                <apply>
                  <times/>
                  <ci> mwe8d5d721_005f_49f7_ada6_e82cd8ca00c6 </ci>
                  <ci> mw4d33db81_6628_421d_8c61_5ef4a4a647ad </ci>
                  <ci> mw70a631d4_9f40_4bbb_bb75_a900f1130935 </ci>
```

```
              </apply>
              <apply>
                <times/>
                <ci> mw75aefaf3_1856_43cf_8511_2fa3e0451e0b </ci>
                <ci> mwc47a95ad_5c77_4a62_b9b7_cb2b8abe3260 </ci>
              </apply>
            </apply>
            <ci> mwaad74090_4b37_47eb_b845_2f28d96c0e82 </ci>
          </apply>
        </math>
      </kineticLaw>
    </reaction>
    <reaction id="mwe37fbb33_a739_4a66_ab96_73daefac9719"
reversible="true" fast="false">
      <notes>
        <body xmlns="http://www.w3.org/1999/xhtml">binding and
unbinding of PD1 PDL2 in synapse</body>
      </notes>
      <listOfReactants>
        <speciesReference
species="mw4d33db81_6628_421d_8c61_5ef4a4a647ad" stoichiometry="1"/>
        <speciesReference
species="mw5b51513e_0500_4cf8_a613_5a8041e539fd" stoichiometry="1"/>
      </listOfReactants>
      <listOfProducts>
        <speciesReference
species="mw345e02b4_624f_415f_af98_e55a9f555853" stoichiometry="1"/>
      </listOfProducts>
      <kineticLaw>
        <math xmlns="http://www.w3.org/1998/Math/MathML">
          <apply>
            <times/>
            <apply>
              <minus/>
              <apply>
                <times/>
                <ci> mw4cfa47a0_69d4_4eec_aa6c_e4470eeeb266 </ci>
                <ci> mw4d33db81_6628_421d_8c61_5ef4a4a647ad </ci>
                <ci> mw5b51513e_0500_4cf8_a613_5a8041e539fd </ci>
              </apply>
              <apply>
                <times/>
                <ci> mw93bf4558_6b97_4f12_bc08_c258a1086db9 </ci>
                <ci> mw345e02b4_624f_415f_af98_e55a9f555853 </ci>
              </apply>
            </apply>
            <ci> mwaad74090_4b37_47eb_b845_2f28d96c0e82 </ci>
          </apply>
        </math>
      </kineticLaw>
    </reaction>
```

```
      <reaction id="mwd4d109eb_dd6a_45ca_980c_212ee9bd45a0"
reversible="true" fast="false">
        <notes>
          <body xmlns="http://www.w3.org/1999/xhtml">binding and
unbinding of PD1 to anti-PD1 on T1 surface in synapse</body>
        </notes>
        <listOfReactants>
          <speciesReference
species="mw4d33db81_6628_421d_8c61_5ef4a4a647ad" stoichiometry="1"/>
        </listOfReactants>
        <listOfProducts>
          <speciesReference
species="mwb8a9b9f0_4e26_4579_acf2_67e8a150f182" stoichiometry="1"/>
        </listOfProducts>
        <kineticLaw>
          <math xmlns="http://www.w3.org/1998/Math/MathML">
            <apply>
              <times/>
              <apply>
                <minus/>
                <apply>
                  <times/>
                  <cn type="integer"> 2 </cn>
                  <ci> mw8db9064f_6e42_49c7_8a0b_a47da71a3758 </ci>
                  <apply>
                    <divide/>
                    <apply>
                      <times/>
                      <ci> mw4d33db81_6628_421d_8c61_5ef4a4a647ad
</ci>
                      <ci> mw75ebeaf4_1dd4_4b61_bdf7_0611af968ca8
</ci>
                    </apply>
                    <ci> mw035bd865_eaf6_4933_a20e_09f1ca241df0 </ci>
                  </apply>
                </apply>
                <apply>
                  <times/>
                  <ci> mw991ab93b_f5ef_47e4_8661_9588c62249e4 </ci>
                  <ci> mwb8a9b9f0_4e26_4579_acf2_67e8a150f182 </ci>
                </apply>
              </apply>
              <ci> mwaad74090_4b37_47eb_b845_2f28d96c0e82 </ci>
            </apply>
          </math>
        </kineticLaw>
      </reaction>
      <reaction id="mwfcee979a_7bc7_400c_9bbb_1ec1ccdf701f"
reversible="true" fast="false">
        <notes>
          <body xmlns="http://www.w3.org/1999/xhtml">binding and
unbinding of PD1 to PD1-Nivo on T1 surface in synapse</body>
```

```
        </notes>
        <listOfReactants>
          <speciesReference
species="mwb8a9b9f0_4e26_4579_acf2_67e8a150f182" stoichiometry="1"/>
          <speciesReference
species="mw4d33db81_6628_421d_8c61_5ef4a4a647ad" stoichiometry="1"/>
        </listOfReactants>
        <listOfProducts>
          <speciesReference
species="mw89f4badf_d6b8_45ff_9012_7780c0a611d2" stoichiometry="1"/>
        </listOfProducts>
        <kineticLaw>
          <math xmlns="http://www.w3.org/1998/Math/MathML">
            <apply>
              <times/>
              <apply>
                <minus/>
                <apply>
                  <times/>
                  <ci> mw614c8d32_2578_4c5c_ba98_2300e829174f </ci>
                  <ci> mw8db9064f_6e42_49c7_8a0b_a47da71a3758 </ci>
                  <ci> mw4d33db81_6628_421d_8c61_5ef4a4a647ad </ci>
                  <ci> mwb8a9b9f0_4e26_4579_acf2_67e8a150f182 </ci>
                </apply>
                <apply>
                  <times/>
                  <cn type="integer"> 2 </cn>
                  <ci> mw991ab93b_f5ef_47e4_8661_9588c62249e4 </ci>
                  <ci> mw89f4badf_d6b8_45ff_9012_7780c0a611d2 </ci>
                </apply>
              </apply>
              <ci> mwaad74090_4b37_47eb_b845_2f28d96c0e82 </ci>
            </apply>
          </math>
        </kineticLaw>
      </reaction>
      <reaction id="mwc2a93bd8_cc01_4a8b_90ee_e8f99f9d3920"
reversible="true" fast="false">
        <notes>
          <body xmlns="http://www.w3.org/1999/xhtml">binding and
unbinding of PDL1 to anit-PDL1 on APC surface in synapse</body>
        </notes>
        <listOfReactants>
          <speciesReference
species="mw70a631d4_9f40_4bbb_bb75_a900f1130935" stoichiometry="1"/>
        </listOfReactants>
        <listOfProducts>
          <speciesReference
species="mwf4dc2ee0_9bd9_4f51_ad22_d377e3e1668e" stoichiometry="1"/>
        </listOfProducts>
        <kineticLaw>
          <math xmlns="http://www.w3.org/1998/Math/MathML">
```

```
              <apply>
                <times/>
                <apply>
                  <minus/>
                  <apply>
                    <times/>
                    <cn type="integer"> 2 </cn>
                    <ci> mwe0f00ed8_dcf4_4859_9755_bb24cd7f35c9 </ci>
                    <apply>
                      <divide/>
                      <apply>
                        <times/>
                        <ci> mw70a631d4_9f40_4bbb_bb75_a900f1130935
</ci>
                        <ci> mw140e5cc4_b897_4719_aa3d_0b30f25bc4dd
</ci>
                      </apply>
                      <ci> mw035bd865_eaf6_4933_a20e_09f1ca241df0 </ci>
                    </apply>
                  </apply>
                  <apply>
                    <times/>
                    <ci> mwe82cc259_fdc8_4239_a6e1_1f0fea41ca6d </ci>
                    <ci> mwf4dc2ee0_9bd9_4f51_ad22_d377e3e1668e </ci>
                  </apply>
                </apply>
                <ci> mwaad74090_4b37_47eb_b845_2f28d96c0e82 </ci>
              </apply>
            </math>
          </kineticLaw>
        </reaction>
        <reaction id="mw3b31abb3_0830_496a_b4b1_41dcee850286"
reversible="true" fast="false">
          <notes>
            <body xmlns="http://www.w3.org/1999/xhtml">binding and
unbinding of PDL1 to PDL1-anti-PDL1 on APC surface in synapse</body>
          </notes>
          <listOfReactants>
            <speciesReference
species="mwf4dc2ee0_9bd9_4f51_ad22_d377e3e1668e" stoichiometry="1"/>
            <speciesReference
species="mw70a631d4_9f40_4bbb_bb75_a900f1130935" stoichiometry="1"/>
          </listOfReactants>
          <listOfProducts>
            <speciesReference
species="mw0c6e5a4d_1f16_46fd_b5b4_bb71908a3fa3" stoichiometry="1"/>
          </listOfProducts>
          <kineticLaw>
            <math xmlns="http://www.w3.org/1998/Math/MathML">
              <apply>
                <times/>
                <apply>
```

```
                <minus/>
                <apply>
                  <times/>
                  <ci> mw3e179651_289c_42d8_bfe9_a4412cee4d9c </ci>
                  <ci> mwe0f00ed8_dcf4_4859_9755_bb24cd7f35c9 </ci>
                  <ci> mw70a631d4_9f40_4bbb_bb75_a900f1130935 </ci>
                  <ci> mwf4dc2ee0_9bd9_4f51_ad22_d377e3e1668e </ci>
                </apply>
                <apply>
                  <times/>
                  <cn type="integer"> 2 </cn>
                  <ci> mwe82cc259_fdc8_4239_a6e1_1f0fea41ca6d </ci>
                  <ci> mw0c6e5a4d_1f16_46fd_b5b4_bb71908a3fa3 </ci>
                </apply>
              </apply>
              <ci> mwaad74090_4b37_47eb_b845_2f28d96c0e82 </ci>
            </apply>
          </math>
        </kineticLaw>
      </reaction>
      <reaction id="mw4c4715c0_6f9c_45e6_a2a7_f37769ac3177"
reversible="true" fast="false">
        <notes>
          <body xmlns="http://www.w3.org/1999/xhtml">cibis diffusive
transport to peripheral compartment</body>
        </notes>
        <listOfReactants>
          <speciesReference
species="mw979043c1_63df_42b5_be8a_697b7f2b0604" stoichiometry="1"/>
        </listOfReactants>
        <listOfProducts>
          <speciesReference
species="mwfa76d65a_154f_40ed_b9e1_0406e01c1120" stoichiometry="1"/>
        </listOfProducts>
        <kineticLaw>
          <math xmlns="http://www.w3.org/1998/Math/MathML">
            <apply>
              <times/>
              <ci> mw36366027_1d43_45e4_a407_edd118db612a </ci>
              <apply>
                <minus/>
                <apply>
                  <divide/>
                  <ci> mw979043c1_63df_42b5_be8a_697b7f2b0604 </ci>
                  <ci> mw8b890aa4_5a8d_494f_a9dc_94dd9102747d </ci>
                </apply>
                <apply>
                  <divide/>
                  <ci> mwfa76d65a_154f_40ed_b9e1_0406e01c1120 </ci>
                  <ci> mw0a99753d_641d_4691_8979_ff41ab8c42c4 </ci>
                </apply>
              </apply>
```

```
            </apply>
          </math>
        </kineticLaw>
      </reaction>
      <reaction id="mwb93962d5_a9d1_4982_8add_285fb45e794f"
reversible="true" fast="false">
        <notes>
          <body xmlns="http://www.w3.org/1999/xhtml">cibis diffusive
transport to tumor compartment</body>
        </notes>
        <listOfReactants>
          <speciesReference
species="mw979043c1_63df_42b5_be8a_697b7f2b0604" stoichiometry="1"/>
        </listOfReactants>
        <listOfProducts>
          <speciesReference
species="mw9c0222d8_0066_4f7c_8803_f18575631cfe" stoichiometry="1"/>
        </listOfProducts>
        <kineticLaw>
          <math xmlns="http://www.w3.org/1998/Math/MathML">
            <apply>
              <times/>
              <ci> mw7ca21d14_d8be_4151_a1ca_04e2bfeabb57 </ci>
              <apply>
                <minus/>
                <apply>
                  <divide/>
                  <ci> mw979043c1_63df_42b5_be8a_697b7f2b0604 </ci>
                  <ci> mw8b890aa4_5a8d_494f_a9dc_94dd9102747d </ci>
                </apply>
                <apply>
                  <divide/>
                  <ci> mw9c0222d8_0066_4f7c_8803_f18575631cfe </ci>
                  <ci> mw9d3532da_0dcb_480c_9ec6_05144794ed9e </ci>
                </apply>
              </apply>
              <ci> mw8cfbefd5_8b10_411d_9d84_300692817ff2 </ci>
            </apply>
          </math>
        </kineticLaw>
      </reaction>
      <reaction id="mw0610e89b_19c6_4de7_801d_df652069080d"
reversible="true" fast="false">
        <notes>
          <body xmlns="http://www.w3.org/1999/xhtml">cibis diffusive
transport to lymph node compartment</body>
        </notes>
        <listOfReactants>
          <speciesReference
species="mw979043c1_63df_42b5_be8a_697b7f2b0604" stoichiometry="1"/>
        </listOfReactants>
        <listOfProducts>
```

```
          <speciesReference
species="mw98d3c290_6a02_4140_b562_ca1a3cb39c99" stoichiometry="1"/>
        </listOfProducts>
        <kineticLaw>
          <math xmlns="http://www.w3.org/1998/Math/MathML">
            <apply>
              <times/>
              <ci> mwe6152cdd_350a_4c16_81ca_1e481e6c0a64 </ci>
              <apply>
                <minus/>
                <apply>
                  <divide/>
                  <ci> mw979043c1_63df_42b5_be8a_697b7f2b0604 </ci>
                  <ci> mw8b890aa4_5a8d_494f_a9dc_94dd9102747d </ci>
                </apply>
                <apply>
                  <divide/>
                  <ci> mw98d3c290_6a02_4140_b562_ca1a3cb39c99 </ci>
                  <ci> mw035bd865_eaf6_4933_a20e_09f1ca241df0 </ci>
                </apply>
              </apply>
            </apply>
          </math>
        </kineticLaw>
      </reaction>
      <reaction id="mw287456aa_0fe2_4148_a149_1b2e9d5e0e01"
reversible="false" fast="false">
        <notes>
          <body xmlns="http://www.w3.org/1999/xhtml">cibis convective
transport from tumor to lymph node</body>
        </notes>
        <listOfReactants>
          <speciesReference
species="mw9c0222d8_0066_4f7c_8803_f18575631cfe" stoichiometry="1"/>
        </listOfReactants>
        <listOfProducts>
          <speciesReference
species="mw98d3c290_6a02_4140_b562_ca1a3cb39c99" stoichiometry="1"/>
        </listOfProducts>
        <kineticLaw>
          <math xmlns="http://www.w3.org/1998/Math/MathML">
            <apply>
              <times/>
              <apply>
                <divide/>
                <apply>
                  <times/>
                  <ci> mw874a6840_c8e2_4455_888c_3f46f6b9f513 </ci>
                  <ci> mw9c0222d8_0066_4f7c_8803_f18575631cfe </ci>
                </apply>
                <ci> mw9d3532da_0dcb_480c_9ec6_05144794ed9e </ci>
              </apply>
```

```
              &lt;ci&gt; mw175e8610_7578_45f5_80bb_7b61e6e241ab &lt;/ci&gt;
            &lt;/apply&gt;
          &lt;/math&gt;
        &lt;/kineticLaw&gt;
      &lt;/reaction&gt;
      <reaction id="mw0b301444_9924_41b5_9ddb_009f57d5df3b"
reversible="false" fast="false">
        &lt;notes&gt;
          &lt;body xmlns="http://www.w3.org/1999/xhtml"&gt;cibis convective
transport from lymph node to central&lt;/body&gt;
        &lt;/notes&gt;
        &lt;listOfReactants&gt;
          <speciesReference
species="mw98d3c290_6a02_4140_b562_ca1a3cb39c99" stoichiometry="1"/>
        &lt;/listOfReactants&gt;
        &lt;listOfProducts&gt;
          <speciesReference
species="mw979043c1_63df_42b5_be8a_697b7f2b0604" stoichiometry="1"/>
        &lt;/listOfProducts&gt;
        &lt;kineticLaw&gt;
          &lt;math xmlns="http://www.w3.org/1998/Math/MathML"&gt;
            &lt;apply&gt;
              &lt;times/&gt;
              &lt;apply&gt;
                &lt;divide/&gt;
                &lt;apply&gt;
                  &lt;times/&gt;
                  &lt;ci&gt; mw874a6840_c8e2_4455_888c_3f46f6b9f513 &lt;/ci&gt;
                  &lt;ci&gt; mw98d3c290_6a02_4140_b562_ca1a3cb39c99 &lt;/ci&gt;
                &lt;/apply&gt;
                &lt;ci&gt; mw035bd865_eaf6_4933_a20e_09f1ca241df0 &lt;/ci&gt;
              &lt;/apply&gt;
              &lt;ci&gt; mwb4901521_e24a_4f39_acff_107e3522faa9 &lt;/ci&gt;
            &lt;/apply&gt;
          &lt;/math&gt;
        &lt;/kineticLaw&gt;
      &lt;/reaction&gt;
      <reaction id="mwe0441f95_7a4a_40d5_9700_eb184e1c6f44"
reversible="false" fast="false">
        &lt;notes&gt;
          &lt;body xmlns="http://www.w3.org/1999/xhtml"&gt;Drug clearance
from central compartment&lt;/body&gt;
        &lt;/notes&gt;
        &lt;listOfReactants&gt;
          <speciesReference
species="mw979043c1_63df_42b5_be8a_697b7f2b0604" stoichiometry="1"/>
        &lt;/listOfReactants&gt;
        &lt;kineticLaw&gt;
          &lt;math xmlns="http://www.w3.org/1998/Math/MathML"&gt;
            &lt;apply&gt;
              &lt;times/&gt;
              &lt;ci&gt; mw41dce75f_74ae_4cf2_8cf1_4733ddbc9d1a &lt;/ci&gt;
```

```
              <ci> mw979043c1_63df_42b5_be8a_697b7f2b0604 </ci>
              <ci> mw8cfbefd5_8b10_411d_9d84_300692817ff2 </ci>
            </apply>
          </math>
        </kineticLaw>
      </reaction>
      <reaction id="mwab2371df_afae_47bd_9d0c_b6038f7aeeb8"
reversible="true" fast="false">
        <notes>
          <body xmlns="http://www.w3.org/1999/xhtml">binding and
unbinding of CEA to cibis on surface in synapse</body>
        </notes>
        <listOfReactants>
          <speciesReference
species="mwa5aae57a_8cb6_4abc_8865_078cae23d61b" stoichiometry="1"/>
        </listOfReactants>
        <listOfProducts>
          <speciesReference
species="mw748d24b3_d226_4e4d_9970_2410b77ccfa1" stoichiometry="1"/>
        </listOfProducts>
        <kineticLaw>
          <math xmlns="http://www.w3.org/1998/Math/MathML">
            <apply>
              <times/>
              <apply>
                <minus/>
                <apply>
                  <times/>
                  <cn type="integer"> 2 </cn>
                  <ci> mw9cf96b22_1a33_4f87_afcd_68dba7e3c4a6 </ci>
                  <apply>
                    <divide/>
                    <apply>
                      <times/>
                      <ci> mwa5aae57a_8cb6_4abc_8865_078cae23d61b
</ci>
                      <ci> mw9c0222d8_0066_4f7c_8803_f18575631cfe
</ci>
                    </apply>
                    <ci> mw9d3532da_0dcb_480c_9ec6_05144794ed9e </ci>
                  </apply>
                </apply>
                <apply>
                  <times/>
                  <ci> mw254b6741_04ff_476a_86b0_e18f1f7448a6 </ci>
                  <ci> mw748d24b3_d226_4e4d_9970_2410b77ccfa1 </ci>
                </apply>
              </apply>
              <ci> mw0305c0ed_ac99_469b_8d32_3127d022edf9 </ci>
            </apply>
          </math>
        </kineticLaw>
```

BMJ Publishing Group Limited (BMJ) disclaims all liability and responsibility arising from any reliance placed on this supplemental material which has been supplied by the author(s)


```
      </reaction>
      <reaction id="mw8f586b3b_8847_4bec_97d7_8954b17055a7"
reversible="true" fast="false">
        <notes>
          <body xmlns="http://www.w3.org/1999/xhtml">binding and
unbinding of CEA to Cibis on surface in synapse</body>
        </notes>
        <listOfReactants>
          <speciesReference
species="mw748d24b3_d226_4e4d_9970_2410b77ccfa1" stoichiometry="1"/>
          <speciesReference
species="mwa5aae57a_8cb6_4abc_8865_078cae23d61b" stoichiometry="1"/>
        </listOfReactants>
        <listOfProducts>
          <speciesReference
species="mw4f3f7cec_48cd_46bc_827c_b7fe282b5a76" stoichiometry="1"/>
        </listOfProducts>
        <kineticLaw>
          <math xmlns="http://www.w3.org/1998/Math/MathML">
            <apply>
              <times/>
              <apply>
                <minus/>
                <apply>
                  <times/>
                  <ci> mwc205da12_9624_4676_ba4c_e1d6d6b18abd </ci>
                  <ci> mw9cf96b22_1a33_4f87_afcd_68dba7e3c4a6 </ci>
                  <ci> mwa5aae57a_8cb6_4abc_8865_078cae23d61b </ci>
                  <ci> mw748d24b3_d226_4e4d_9970_2410b77ccfa1 </ci>
                </apply>
                <apply>
                  <times/>
                  <cn type="integer"> 2 </cn>
                  <ci> mw254b6741_04ff_476a_86b0_e18f1f7448a6 </ci>
                  <ci> mw4f3f7cec_48cd_46bc_827c_b7fe282b5a76 </ci>
                </apply>
              </apply>
              <ci> mw0305c0ed_ac99_469b_8d32_3127d022edf9 </ci>
            </apply>
          </math>
        </kineticLaw>
      </reaction>
      <reaction id="mw4772715f_3370_4258_8dbe_d46088b614ae"
reversible="true" fast="false">
        <notes>
          <body xmlns="http://www.w3.org/1999/xhtml">binding and
unbinding of CEA to Cibis on surface in synapse</body>
        </notes>
        <listOfReactants>
          <speciesReference
species="mw748d24b3_d226_4e4d_9970_2410b77ccfa1" stoichiometry="1"/>
```

```
          <speciesReference
species="mw5f168fc1_14e9_4b01_916e_4962d6278d2e" stoichiometry="1"/>
        </listOfReactants>
        <listOfProducts>
          <speciesReference
species="mw0904c3c8_c9cd_43fd_82b9_e8a5772b6404" stoichiometry="1"/>
        </listOfProducts>
        <kineticLaw>
          <math xmlns="http://www.w3.org/1998/Math/MathML">
            <apply>
              <times/>
              <apply>
                <minus/>
                <apply>
                  <divide/>
                  <apply>
                    <times/>
                    <ci> mw15df1616_97ed_4ca4_b30e_ebb077cf4325 </ci>
                    <ci> mw5f168fc1_14e9_4b01_916e_4962d6278d2e </ci>
                    <ci> mw748d24b3_d226_4e4d_9970_2410b77ccfa1 </ci>
                  </apply>
                  <ci> mwfffe4beb_6c50_42ac_8f60_6131e3e8cc8b </ci>
                </apply>
                <apply>
                  <times/>
                  <ci> mwf8e07c6f_de10_40ed_8fed_c4b415ac88ff </ci>
                  <ci> mw0904c3c8_c9cd_43fd_82b9_e8a5772b6404 </ci>
                </apply>
              </apply>
              <ci> mw0305c0ed_ac99_469b_8d32_3127d022edf9 </ci>
            </apply>
          </math>
        </kineticLaw>
      </reaction>
      <reaction id="mw0c92b174_8aef_40ee_97a6_9acb43abea10"
reversible="true" fast="false">
        <notes>
          <body xmlns="http://www.w3.org/1999/xhtml">binding and
unbinding of CD3 to CEA_cibis on in synapse</body>
        </notes>
        <listOfReactants>
          <speciesReference
species="mw4f3f7cec_48cd_46bc_827c_b7fe282b5a76" stoichiometry="1"/>
          <speciesReference
species="mw5f168fc1_14e9_4b01_916e_4962d6278d2e" stoichiometry="1"/>
        </listOfReactants>
        <listOfProducts>
          <speciesReference
species="mwf1568170_3b99_448b_94b1_035796c05f2f" stoichiometry="1"/>
        </listOfProducts>
        <kineticLaw>
          <math xmlns="http://www.w3.org/1998/Math/MathML">
```

```
          <apply>
            <times/>
            <apply>
              <minus/>
              <apply>
                <divide/>
                <apply>
                  <times/>
                  <ci> mw15df1616_97ed_4ca4_b30e_ebb077cf4325 </ci>
                  <ci> mw5f168fc1_14e9_4b01_916e_4962d6278d2e </ci>
                  <ci> mw4f3f7cec_48cd_46bc_827c_b7fe282b5a76 </ci>
                </apply>
                <ci> mwfffe4beb_6c50_42ac_8f60_6131e3e8cc8b </ci>
              </apply>
              <apply>
                <times/>
                <ci> mwf8e07c6f_de10_40ed_8fed_c4b415ac88ff </ci>
                <ci> mwf1568170_3b99_448b_94b1_035796c05f2f </ci>
              </apply>
            </apply>
            <ci> mw0305c0ed_ac99_469b_8d32_3127d022edf9 </ci>
          </apply>
        </math>
      </kineticLaw>
    </reaction>
    <reaction id="mw54abc915_968c_46f4_8a8b_41d008db6132"
reversible="true" fast="false">
      <notes>
        <body xmlns="http://www.w3.org/1999/xhtml">binding and
unbinding of CD3 to cibis on surface in synapse</body>
      </notes>
      <listOfReactants>
        <speciesReference
species="mw5f168fc1_14e9_4b01_916e_4962d6278d2e" stoichiometry="1"/>
      </listOfReactants>
      <listOfProducts>
        <speciesReference
species="mwd684b838_9206_4f20_b576_f4313889c57f" stoichiometry="1"/>
      </listOfProducts>
      <kineticLaw>
        <math xmlns="http://www.w3.org/1998/Math/MathML">
          <apply>
            <times/>
            <apply>
              <minus/>
              <apply>
                <times/>
                <ci> mw15df1616_97ed_4ca4_b30e_ebb077cf4325 </ci>
                <apply>
                  <divide/>
                  <apply>
                    <times/>
```

```
                      &lt;ci&gt; mw5f168fc1_14e9_4b01_916e_4962d6278d2e
&lt;/ci&gt;
                      &lt;ci&gt; mw9c0222d8_0066_4f7c_8803_f18575631cfe
&lt;/ci&gt;
                    &lt;/apply&gt;
                    &lt;ci&gt; mw9d3532da_0dcb_480c_9ec6_05144794ed9e &lt;/ci&gt;
                  &lt;/apply&gt;
                &lt;/apply&gt;
                &lt;apply&gt;
                  &lt;times/&gt;
                  &lt;ci&gt; mwf8e07c6f_de10_40ed_8fed_c4b415ac88ff &lt;/ci&gt;
                  &lt;ci&gt; mwd684b838_9206_4f20_b576_f4313889c57f &lt;/ci&gt;
                &lt;/apply&gt;
              &lt;/apply&gt;
              &lt;ci&gt; mw0305c0ed_ac99_469b_8d32_3127d022edf9 &lt;/ci&gt;
            &lt;/apply&gt;
          &lt;/math&gt;
        &lt;/kineticLaw&gt;
      &lt;/reaction&gt;
      <reaction id="mw25cdcd1d_6e81_43c5_a90b_1264267ca78c"
reversible="true" fast="false">
        &lt;notes&gt;
          &lt;body xmlns="http://www.w3.org/1999/xhtml"&gt;binding and
unbinding of CEA to CD3_cibis on in synapse&lt;/body&gt;
        &lt;/notes&gt;
        &lt;listOfReactants&gt;
          <speciesReference
species="mwd684b838_9206_4f20_b576_f4313889c57f" stoichiometry="1"/>
          <speciesReference
species="mwa5aae57a_8cb6_4abc_8865_078cae23d61b" stoichiometry="1"/>
        &lt;/listOfReactants&gt;
        &lt;listOfProducts&gt;
          <speciesReference
species="mw0904c3c8_c9cd_43fd_82b9_e8a5772b6404" stoichiometry="1"/>
        &lt;/listOfProducts&gt;
        &lt;kineticLaw&gt;
          &lt;math xmlns="http://www.w3.org/1998/Math/MathML"&gt;
            &lt;apply&gt;
              &lt;times/&gt;
              &lt;apply&gt;
                &lt;minus/&gt;
                &lt;apply&gt;
                  &lt;divide/&gt;
                  &lt;apply&gt;
                    &lt;times/&gt;
                    &lt;cn type="integer"&gt; 2 &lt;/cn&gt;
                    &lt;ci&gt; mw9cf96b22_1a33_4f87_afcd_68dba7e3c4a6 &lt;/ci&gt;
                    &lt;ci&gt; mwd684b838_9206_4f20_b576_f4313889c57f &lt;/ci&gt;
                    &lt;ci&gt; mwa5aae57a_8cb6_4abc_8865_078cae23d61b &lt;/ci&gt;
                  &lt;/apply&gt;
                  &lt;ci&gt; mwfffe4beb_6c50_42ac_8f60_6131e3e8cc8b &lt;/ci&gt;
                &lt;/apply&gt;
```

```
                <apply>
                  <times/>
                  <ci> mw254b6741_04ff_476a_86b0_e18f1f7448a6 </ci>
                  <ci> mw0904c3c8_c9cd_43fd_82b9_e8a5772b6404 </ci>
                </apply>
              </apply>
              <ci> mw0305c0ed_ac99_469b_8d32_3127d022edf9 </ci>
            </apply>
          </math>
        </kineticLaw>
      </reaction>
      <reaction id="mwaebd1042_2a86_4fdf_8306_8fc137ff3d55"
reversible="true" fast="false">
        <notes>
          <body xmlns="http://www.w3.org/1999/xhtml">binding and
unbinding of CEA to Cibis on surface in synapse</body>
        </notes>
        <listOfReactants>
          <speciesReference
species="mw0904c3c8_c9cd_43fd_82b9_e8a5772b6404" stoichiometry="1"/>
          <speciesReference
species="mwa5aae57a_8cb6_4abc_8865_078cae23d61b" stoichiometry="1"/>
        </listOfReactants>
        <listOfProducts>
          <speciesReference
species="mwf1568170_3b99_448b_94b1_035796c05f2f" stoichiometry="1"/>
        </listOfProducts>
        <kineticLaw>
          <math xmlns="http://www.w3.org/1998/Math/MathML">
            <apply>
              <times/>
              <apply>
                <minus/>
                <apply>
                  <times/>
                  <ci> mwc205da12_9624_4676_ba4c_e1d6d6b18abd </ci>
                  <ci> mw9cf96b22_1a33_4f87_afcd_68dba7e3c4a6 </ci>
                  <ci> mwa5aae57a_8cb6_4abc_8865_078cae23d61b </ci>
                  <ci> mw0904c3c8_c9cd_43fd_82b9_e8a5772b6404 </ci>
                </apply>
                <apply>
                  <times/>
                  <cn type="integer"> 2 </cn>
                  <ci> mw254b6741_04ff_476a_86b0_e18f1f7448a6 </ci>
                  <ci> mwf1568170_3b99_448b_94b1_035796c05f2f </ci>
                </apply>
              </apply>
              <ci> mw0305c0ed_ac99_469b_8d32_3127d022edf9 </ci>
            </apply>
          </math>
        </kineticLaw>
      </reaction>
```

```
      <reaction id="mw4472f77f_4856_4ae7_808d_c09b10769e13"
reversible="true" fast="false">
        <notes>
          <body xmlns="http://www.w3.org/1999/xhtml">binding and
unbinding of CEA to cibis on surface in synapse</body>
        </notes>
        <listOfReactants>
          <speciesReference
species="mw8cd989f8_196a_44a9_a449_5f7d44e2c0cf" stoichiometry="1"/>
        </listOfReactants>
        <listOfProducts>
          <speciesReference
species="mwdc4ebba0_e2e2_420e_ab38_3dd8a8a9a4b0" stoichiometry="1"/>
        </listOfProducts>
        <kineticLaw>
          <math xmlns="http://www.w3.org/1998/Math/MathML">
            <apply>
              <times/>
              <apply>
                <minus/>
                <apply>
                  <times/>
                  <cn type="integer"> 2 </cn>
                  <ci> mw9cf96b22_1a33_4f87_afcd_68dba7e3c4a6 </ci>
                  <apply>
                    <divide/>
                    <apply>
                      <times/>
                      <ci> mw8cd989f8_196a_44a9_a449_5f7d44e2c0cf
</ci>
                      <ci> mw9c0222d8_0066_4f7c_8803_f18575631cfe
</ci>
                    </apply>
                    <ci> mw9d3532da_0dcb_480c_9ec6_05144794ed9e </ci>
                  </apply>
                </apply>
                <apply>
                  <times/>
                  <ci> mw254b6741_04ff_476a_86b0_e18f1f7448a6 </ci>
                  <ci> mwdc4ebba0_e2e2_420e_ab38_3dd8a8a9a4b0 </ci>
                </apply>
              </apply>
              <ci> mwfc416eeb_b169_4568_9e44_cab5996f5ee2 </ci>
            </apply>
          </math>
        </kineticLaw>
      </reaction>
      <reaction id="mw607981f9_e5e5_4d29_93ff_53dff65bc9de"
reversible="true" fast="false">
        <notes>
          <body xmlns="http://www.w3.org/1999/xhtml">binding and
unbinding of CEA to Cibis on surface in synapse</body>
```

```
        </notes>
        <listOfReactants>
          <speciesReference
species="mwdc4ebba0_e2e2_420e_ab38_3dd8a8a9a4b0" stoichiometry="1"/>
          <speciesReference
species="mw8cd989f8_196a_44a9_a449_5f7d44e2c0cf" stoichiometry="1"/>
        </listOfReactants>
        <listOfProducts>
          <speciesReference
species="mw31898027_19ab_4432_8d21_20da25633a64" stoichiometry="1"/>
        </listOfProducts>
        <kineticLaw>
          <math xmlns="http://www.w3.org/1998/Math/MathML">
            <apply>
              <times/>
              <apply>
                <minus/>
                <apply>
                  <times/>
                  <ci> mwc205da12_9624_4676_ba4c_e1d6d6b18abd </ci>
                  <ci> mw9cf96b22_1a33_4f87_afcd_68dba7e3c4a6 </ci>
                  <ci> mw8cd989f8_196a_44a9_a449_5f7d44e2c0cf </ci>
                  <ci> mwdc4ebba0_e2e2_420e_ab38_3dd8a8a9a4b0 </ci>
                </apply>
                <apply>
                  <times/>
                  <cn type="integer"> 2 </cn>
                  <ci> mw254b6741_04ff_476a_86b0_e18f1f7448a6 </ci>
                  <ci> mw31898027_19ab_4432_8d21_20da25633a64 </ci>
                </apply>
              </apply>
              <ci> mwfc416eeb_b169_4568_9e44_cab5996f5ee2 </ci>
            </apply>
          </math>
        </kineticLaw>
      </reaction>
      <reaction id="mwf15aa5d5_1cf5_4bd1_9bcd_359619812286"
reversible="true" fast="false">
        <notes>
          <body xmlns="http://www.w3.org/1999/xhtml">binding and
unbinding of CEA to Cibis on surface in synapse</body>
        </notes>
        <listOfReactants>
          <speciesReference
species="mwdc4ebba0_e2e2_420e_ab38_3dd8a8a9a4b0" stoichiometry="1"/>
          <speciesReference
species="mw16bdbdfb_62b5_45b6_987d_fae9a564f93e" stoichiometry="1"/>
        </listOfReactants>
        <listOfProducts>
          <speciesReference
species="mw7b471287_592e_46b7_8683_9006b4bed437" stoichiometry="1"/>
        </listOfProducts>
```

```
        <kineticLaw>
          <math xmlns="http://www.w3.org/1998/Math/MathML">
            <apply>
              <times/>
              <apply>
                <minus/>
                <apply>
                  <divide/>
                  <apply>
                    <times/>
                    <ci> mw15df1616_97ed_4ca4_b30e_ebb077cf4325 </ci>
                    <ci> mw16bdbdfb_62b5_45b6_987d_fae9a564f93e </ci>
                    <ci> mwdc4ebba0_e2e2_420e_ab38_3dd8a8a9a4b0 </ci>
                  </apply>
                  <ci> mwfffe4beb_6c50_42ac_8f60_6131e3e8cc8b </ci>
                </apply>
                <apply>
                  <times/>
                  <ci> mwf8e07c6f_de10_40ed_8fed_c4b415ac88ff </ci>
                  <ci> mw7b471287_592e_46b7_8683_9006b4bed437 </ci>
                </apply>
              </apply>
              <ci> mwfc416eeb_b169_4568_9e44_cab5996f5ee2 </ci>
            </apply>
          </math>
        </kineticLaw>
      </reaction>
      <reaction id="mw96b04f0e_99fd_42a5_ba3b_af7b08042c4e"
reversible="true" fast="false">
        <notes>
          <body xmlns="http://www.w3.org/1999/xhtml">binding and
unbinding of CD3 to CEA_cibis on in synapse</body>
        </notes>
        <listOfReactants>
          <speciesReference
species="mw31898027_19ab_4432_8d21_20da25633a64" stoichiometry="1"/>
          <speciesReference
species="mw16bdbdfb_62b5_45b6_987d_fae9a564f93e" stoichiometry="1"/>
        </listOfReactants>
        <listOfProducts>
          <speciesReference
species="mw5c8acb40_e149_4324_a7a2_eadf8fda3445" stoichiometry="1"/>
        </listOfProducts>
        <kineticLaw>
          <math xmlns="http://www.w3.org/1998/Math/MathML">
            <apply>
              <times/>
              <apply>
                <minus/>
                <apply>
                  <divide/>
                  <apply>
```

```
                <times/>
                <ci> mw15df1616_97ed_4ca4_b30e_ebb077cf4325 </ci>
                <ci> mw16bdbdfb_62b5_45b6_987d_fae9a564f93e </ci>
                <ci> mw31898027_19ab_4432_8d21_20da25633a64 </ci>
              </apply>
              <ci> mwfffe4beb_6c50_42ac_8f60_6131e3e8cc8b </ci>
            </apply>
            <apply>
              <times/>
              <ci> mwf8e07c6f_de10_40ed_8fed_c4b415ac88ff </ci>
              <ci> mw5c8acb40_e149_4324_a7a2_eadf8fda3445 </ci>
            </apply>
          </apply>
          <ci> mwfc416eeb_b169_4568_9e44_cab5996f5ee2 </ci>
        </apply>
      </math>
    </kineticLaw>
  </reaction>
  <reaction id="mwa4138c41_4ba0_47d7_b2ff_da3b9df699af"
reversible="true" fast="false">
    <notes>
      <body xmlns="http://www.w3.org/1999/xhtml">binding and
unbinding of CD3 to cibis on surface in synapse</body>
    </notes>
    <listOfReactants>
      <speciesReference
species="mw16bdbdfb_62b5_45b6_987d_fae9a564f93e" stoichiometry="1"/>
    </listOfReactants>
    <listOfProducts>
      <speciesReference
species="mw41e864d0_1566_4d72_ba8a_d6991da393ff" stoichiometry="1"/>
    </listOfProducts>
    <kineticLaw>
      <math xmlns="http://www.w3.org/1998/Math/MathML">
        <apply>
          <times/>
          <apply>
            <minus/>
            <apply>
              <times/>
              <ci> mw15df1616_97ed_4ca4_b30e_ebb077cf4325 </ci>
              <apply>
                <divide/>
                <apply>
                  <times/>
                  <ci> mw16bdbdfb_62b5_45b6_987d_fae9a564f93e
</ci>
                  <ci> mw9c0222d8_0066_4f7c_8803_f18575631cfe
</ci>
                </apply>
                <ci> mw9d3532da_0dcb_480c_9ec6_05144794ed9e </ci>
              </apply>
```

```
                </apply>
                <apply>
                  <times/>
                  <ci> mwf8e07c6f_de10_40ed_8fed_c4b415ac88ff </ci>
                  <ci> mw41e864d0_1566_4d72_ba8a_d6991da393ff </ci>
                </apply>
              </apply>
              <ci> mwfc416eeb_b169_4568_9e44_cab5996f5ee2 </ci>
            </apply>
          </math>
        </kineticLaw>
      </reaction>
      <reaction id="mwc17a83dc_ac16_43c6_9fbd_9175b37dc4e5"
reversible="true" fast="false">
        <notes>
          <body xmlns="http://www.w3.org/1999/xhtml">binding and
unbinding of CEA to CD3_cibis on in synapse</body>
        </notes>
        <listOfReactants>
          <speciesReference
species="mw41e864d0_1566_4d72_ba8a_d6991da393ff" stoichiometry="1"/>
          <speciesReference
species="mw8cd989f8_196a_44a9_a449_5f7d44e2c0cf" stoichiometry="1"/>
        </listOfReactants>
        <listOfProducts>
          <speciesReference
species="mw7b471287_592e_46b7_8683_9006b4bed437" stoichiometry="1"/>
        </listOfProducts>
        <kineticLaw>
          <math xmlns="http://www.w3.org/1998/Math/MathML">
            <apply>
              <times/>
              <apply>
                <minus/>
                <apply>
                  <divide/>
                  <apply>
                    <times/>
                    <cn type="integer"> 2 </cn>
                    <ci> mw9cf96b22_1a33_4f87_afcd_68dba7e3c4a6 </ci>
                    <ci> mw41e864d0_1566_4d72_ba8a_d6991da393ff </ci>
                    <ci> mw8cd989f8_196a_44a9_a449_5f7d44e2c0cf </ci>
                  </apply>
                  <ci> mwfffe4beb_6c50_42ac_8f60_6131e3e8cc8b </ci>
                </apply>
                <apply>
                  <times/>
                  <ci> mw254b6741_04ff_476a_86b0_e18f1f7448a6 </ci>
                  <ci> mw7b471287_592e_46b7_8683_9006b4bed437 </ci>
                </apply>
              </apply>
              <ci> mwfc416eeb_b169_4568_9e44_cab5996f5ee2 </ci>
```

```
            </apply>
          </math>
        </kineticLaw>
      </reaction>
      <reaction id="mw2dcc3ee3_d910_49a1_84cf_ca634e88845f"
reversible="true" fast="false">
        <notes>
          <body xmlns="http://www.w3.org/1999/xhtml">binding and
unbinding of CEA to Cibis on surface in synapse</body>
        </notes>
        <listOfReactants>
          <speciesReference
species="mw7b471287_592e_46b7_8683_9006b4bed437" stoichiometry="1"/>
          <speciesReference
species="mw8cd989f8_196a_44a9_a449_5f7d44e2c0cf" stoichiometry="1"/>
        </listOfReactants>
        <listOfProducts>
          <speciesReference
species="mw5c8acb40_e149_4324_a7a2_eadf8fda3445" stoichiometry="1"/>
        </listOfProducts>
        <kineticLaw>
          <math xmlns="http://www.w3.org/1998/Math/MathML">
            <apply>
              <times/>
              <apply>
                <minus/>
                <apply>
                  <times/>
                  <ci> mwc205da12_9624_4676_ba4c_e1d6d6b18abd </ci>
                  <ci> mw9cf96b22_1a33_4f87_afcd_68dba7e3c4a6 </ci>
                  <ci> mw8cd989f8_196a_44a9_a449_5f7d44e2c0cf </ci>
                  <ci> mw7b471287_592e_46b7_8683_9006b4bed437 </ci>
                </apply>
                <apply>
                  <times/>
                  <cn type="integer"> 2 </cn>
                  <ci> mw254b6741_04ff_476a_86b0_e18f1f7448a6 </ci>
                  <ci> mw5c8acb40_e149_4324_a7a2_eadf8fda3445 </ci>
                </apply>
              </apply>
              <ci> mwfc416eeb_b169_4568_9e44_cab5996f5ee2 </ci>
            </apply>
          </math>
        </kineticLaw>
      </reaction>
    </listOfReactions>
    <listOfEvents>
      <event id="mw4c148330_86c4_4af1_bc70_c9a6f81412b6">
        <trigger>
          <math xmlns="http://www.w3.org/1998/Math/MathML">
            <apply>
              <lt/>
```

```
              <ci> mw2aafd721_12db_4909_b572_82abf04c578d </ci>
              <apply>
                <times/>
                <cn> 0.9 </cn>
                <ci> mw5c6752bd_6029_486c_b439_45f7ca1b3111 </ci>
              </apply>
            </apply>
          </math>
        </trigger>
        <listOfEventAssignments>
          <eventAssignment
variable="mw2aafd721_12db_4909_b572_82abf04c578d">
            <math xmlns="http://www.w3.org/1998/Math/MathML">
              <apply>
                <times/>
                <cn type="integer"> 0 </cn>
                <ci> mw5c6752bd_6029_486c_b439_45f7ca1b3111 </ci>
              </apply>
            </math>
          </eventAssignment>
        </listOfEventAssignments>
      </event>
    </listOfEvents>
  </model>
</sbml>
```
